# The heterozygous pineapple genome demonstrates the importance of haplotype-resolved plant genomes

**DOI:** 10.1093/hr/uhag189

**Published:** 2026-05-08

**Authors:** Jingping Fang, Patrick J Mason, Garth Sanewski, Matthew Webb, Linwei Zhou, Jinbin Wang, Rhys G R Copeland, Ying Zhuang, Annapurna Chitikineni, Vanika Garg, Natalie Dillon, Parwinder Kaur, Rajeev K Varshney, Robert J Henry

**Affiliations:** College of Life Science, Fujian Normal University, Fuzhou 350117, China; Queensland Alliance of Agriculture and Food Innovation, The University of Queensland, St Lucia, Queensland 4072, Australia; Queensland Alliance of Agriculture and Food Innovation, The University of Queensland, St Lucia, Queensland 4072, Australia; ARC Centre of Excellence for Plant Success in Nature and Agriculture, The University of Queensland, St Lucia, Queensland 4072, Australia; Queensland Department of Primary Industries, EcoSciences Precinct, Dutton Park, Queensland 4102, Australia; Queensland Department of Primary Industries, EcoSciences Precinct, Dutton Park, Queensland 4102, Australia; College of Life Science, Fujian Normal University, Fuzhou 350117, China; College of Life Science, Fujian Normal University, Fuzhou 350117, China; WA State Agricultural Biotechnology Centre, Centre for Crop and Food Innovation, Food Futures Institute, Murdoch University, Murdoch, WA 6150, Australia; College of Life Science, Fujian Normal University, Fuzhou 350117, China; WA State Agricultural Biotechnology Centre, Centre for Crop and Food Innovation, Food Futures Institute, Murdoch University, Murdoch, WA 6150, Australia; WA State Agricultural Biotechnology Centre, Centre for Crop and Food Innovation, Food Futures Institute, Murdoch University, Murdoch, WA 6150, Australia; Queensland Department of Primary Industries, EcoSciences Precinct, Dutton Park, Queensland 4102, Australia; School of Agriculture and Environment, University of Western Australia, 35 Stirling Way, Crawley, Western Australia 6009, Australia; WA State Agricultural Biotechnology Centre, Centre for Crop and Food Innovation, Food Futures Institute, Murdoch University, Murdoch, WA 6150, Australia; Queensland Alliance of Agriculture and Food Innovation, The University of Queensland, St Lucia, Queensland 4072, Australia; ARC Centre of Excellence for Plant Success in Nature and Agriculture, The University of Queensland, St Lucia, Queensland 4072, Australia; VinUni Big Data Research Institute, VinUniversity, Hanoi, Vietnam

## Abstract

Collapsed (haploid) genome assemblies omit large portions of genetic information, especially in heterozygous, clonally propagated crops such as pineapple. Here, we assembled a telomere-to-telomere, haplotype-resolved genome for a key pre-Colombian cultivar of pineapple (*Ananas comosus*) ‘Smooth Cayenne’ (F180) using PacBio HiFi and Hi-C data. The two 25-chromosome haplotypes span 858 Mb (N50 ≈ 16.8 Mb) and are >99% complete, each resolving all centromeres and 22 of 25 telomeres. Comparison of the phased chromosomes reveals 1.5 million single nucleotide polymorphisms and 1953 large structural variants (74 inversions, 750 translocations, and 1129 segmental duplications). This assembly reveals that inversions have profoundly impacted the ‘Smooth Cayenne’ genome, reshaping ~3%–4% of the total sequence. Structural context dictates the genetic impact of these large inversions, as shown in recombination landscape analysis of 374 F_1_ seedlings, wherein a 1.3-Mb paracentric inversion on chromosome 20 forms a strict recombination coldspot, whereas a 6-Mb pericentric inversion on chromosome 24 still permits gene flow likely via short double crossovers, albeit at lower rates than the rest of the chromosome. Re-anchoring the 11 879 DArTseq markers from the F_1_ seedlings to the phased reference assembly removes the dense network of spurious interchromosomal linkage seen in the collapsed F153 ‘Smooth Cayenne’ genome, likely providing markedly cleaner baselines for genome-wide association studies and genomic prediction. These results establish the new F180 assembly as a very high-quality reference, illustrate how undetected inversions can silently constrain genetic gain, and demonstrate the broader value of phased genomes for dissecting heterozygosity, structural variation, and meiotic behaviour in perennial crops.

## Introduction

Collapsed assemblies of diploid or polyploid organisms provide only a partial representation of their genomes, as they capture only one version of each heterozygous region [1]. This limitation is particularly problematic in highly heterozygous genomes, where valuable genetic variation is lost. Compounding this issue, early genome assemblies based on short-read sequencing or low-accuracy long reads often produced fragmented, collapsed assemblies, especially in heterozygous species [2, 3]. The availability of highly accurate long-read and high-throughput chromosome conformation capture (Hi-C) [4], alongside new assemblers [5], has enabled the sequencing and assembly of very high-quality haplotype-resolved telomere-to-telomere (T2T) genomes. Genomic fragmentation and the collapsed nature of these earlier assemblies greatly limit their utility for fundamental genetic studies and breeding programmes. For instance, in blueberry, a phased genome assembly enabled more accurate single nucleotide polymorphism (SNP) identification and quantitative trait locus (QTL) mapping, resulting in improved genome-wide association study (GWAS) outcomes and selection of informative markers for genomic selection [6]. This demonstrates how haplotype-resolved assemblies outperform collapsed ones by providing more accurate identification of SNPs and QTLs for breeding and fundamental plant genetics.

Unlike SNPs and small InDels, large structural variants (SVs), particularly inversions, can profoundly impact genomic architecture by forming haploblocks that suppress recombination and alter the evolutionary and breeding trajectories of linked loci [7–11]. In heterozygotes, single crossovers (SCOs) within an inversion generate unbalanced chromatids, which in turn create unviable gametes, suppress recombination, and lead to large blocks of alleles within a chromosomal region being inherited as a block [12]. However, this suppression is not absolute. Analysis of insect [13, 14] and, more recently, plant genomes [8, 15] shows that recombination can occasionally leak through via double crossovers (DCOs), inversion toggling, or short gene conversion tracts, creating a spectrum from ‘tight’ to ‘leaky’ inversions. Differences in inversion size, position relative to the centromere, local sequence context, or age govern this spectrum, although the contribution of these remains poorly resolved in most crops [16, 17]. Resolving both haplotypes in a T2T assembly is therefore a prerequisite to discovering, genotyping, and ultimately exploiting polymorphic inversions in breeding programmes. Recent phased-assembly work in the clonally propagated perennial fruit, mango (*Mangifera indica*), demonstrated that both pericentric and paracentric inversions can harbour breeding-relevant loci and show heterogeneous patterns of recombination suppression [11]. However, equivalent high-resolution analyses are still lacking in many other clonally propagated fruit crops.

Several economically important crops, particularly those propagated vegetatively for centuries or millennia, exhibit elevated heterozygosity [18]. This diversity, preserved through clonal propagation, may contribute to traits such as hybrid vigour or resilience to environmental stresses, thereby sustaining certain genotypes despite minimal genetic improvement [19]. Such heterozygosity can be linked to a variety of factors, including self-incompatibility and vegetative propagation over thousands of years, leading to the accumulation of different somatic mutations in each haplotype. This high heterozygosity may also originate from wide hybridizations in their evolutionary past, i.e. crosses between genetically distinct lineages [20], maintained via vegetative reproduction. However, direct evidence for such hybrid origins in pineapple remains limited due to the ancient nature of its domestication history. Understanding haplotype-specific variation in these crops is essential for deciphering the genomic basis of their adaptability and enhancing modern breeding strategies [21].

Pineapple (*Ananas comosus* var. *comosus*), domesticated from its wild progenitor *A. comosus* var. *microstachys* [22], exemplifies this paradigm. Cultivated through a combination of sexual recombination, self-incompatibility, and clonal propagation since its ancient domestication, pineapple maintains a highly heterozygous genome [23]. As a globally important tropical fruit, pineapple production is constrained by a narrow genetic base [24], exacerbated by reliance on a limited number of cultivars, particularly ‘Smooth Cayenne’, making it vulnerable to pathogens and abiotic stresses [25, 26]. In this study, we report a haplotype-resolved T2T genome assembly of ‘Smooth Cayenne’ (F180 selection), a key pre-Columbian variety of pineapple. This haplotype-resolved genome assembly represents a substantial improvement over previous pineapple genome assemblies [23, 27] and provides a foundational reference for understanding heterozygosity, SVs, and recombination dynamics in clonally propagated crops.

## Results

### *De novo* haplotype-resolved genome assembly of Smooth Cayenne

**Figure 1:**
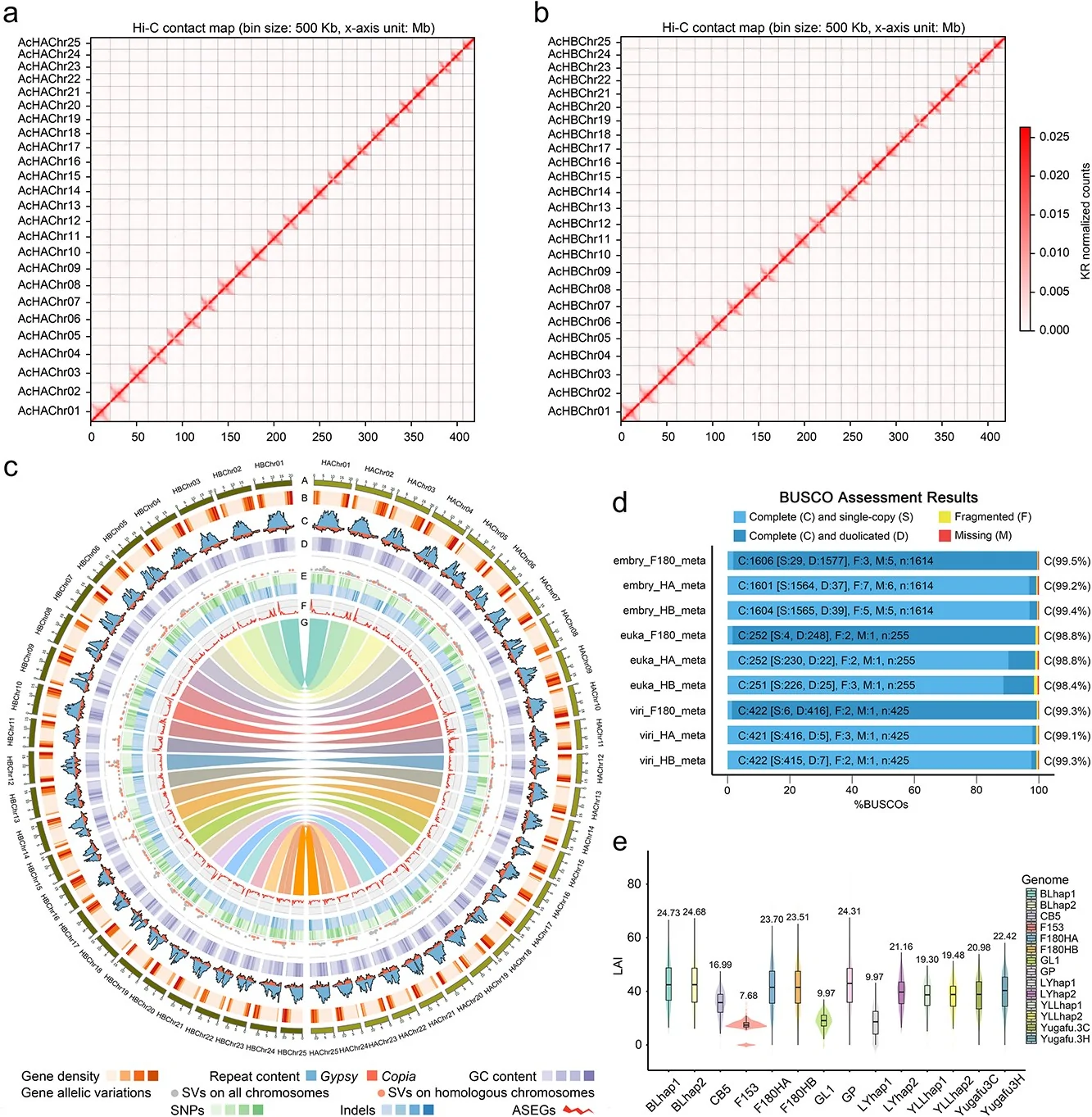
An overview of the ‘Smooth Cayenne’ F180 reference genome. (a and b) Hi-C interaction heatmaps represent contact matrices of 25 chromosomes (2*n* = 50) in F180; (a) HA genome and (b) HB genome at 500-kb resolution. (c) The circos plot of the F180 genome at 500 kb intervals. The tracks represent the following elements (from outer to inner): (A) chromosomes (in Mb), (B) gene density, (C) repeat content: LTR/*Gypsy* and *Copia*, (D) GC content, (E) gene allelic variations: SV on all and homologous chromosomes, SNPs, and InDels, (F) ASEGs. (d) The completeness of the F180 genome, including HA, HB, and the whole genome assessed in BUSCO (v5.8.0) with MetaEuk tool, based on the latest embryophyta_odb10, eukaryota_odb10, and viridiplantae_odb10 database. (e) The violin plot of the longest terminal repeat (LTR) index (LAI) among nine pineapple genome assemblies at the chromosome level. The horizontal axis represents different accessions, while the number in each box indicates the LAI value.

The ‘Smooth Cayenne’ (F180) genome was assembled using PacBio high-fidelity (HiFi) and Hi-C sequencing data. In total, we obtained 46.33 Gb (∼47×) of HiFi reads with an N50 of 27 kb and 39.86 Gb (∼40×) of Hi-C data (Fig. S1, Table S1). GenomeScope analysis estimated a heterozygosity of 1.57% based on a *k*-mer of 21 (Fig. S2). To achieve the T2T haplotype-resolved genome assembly, we adopted a haplotype-aware pipeline integrating HiFi and Hi-C reads to assemble the phased genome (Fig. S3). Chromosomes of both haplotypes were manually ordered by size with short arms forward, and their correspondence to F153 [23] is provided in Table S2. After iterative manual refinement, the Hi-C contact maps presented high consistency across all chromosomes, with strong diagonal signals and clear T2T interactions, supporting the accuracy of chromosome ordering and orientation (Fig. 1a and b). Following removal of organelle-derived and redundant contigs, a nearly complete phased reference genome was generated, consisting of 50 pseudo-chromosomes (25 per haplotype) totalling 858.47 Mb (433.27 and 425.20 Mb, respectively) (Fig. 1c, Table 1). The chromosome lengths for haplotype A (HA) ranged from 12.49 to 21.07 Mb (N50 = 16.52 Mb), while the lengths for haplotype B (HB) ranged from 12.09 to 20.48 Mb (N50 = 16.86 Mb) (Table S3). Telomeric repeat motifs were detected at both ends of 22 chromosomes in each haplotype, and all 50 centromeres were identified (Figs S4 and S5 and Tables S3 and S4). A completely collapsed genome, where two haplotypes were merged and parental alleles randomly switched within a contig, could not be assembled due to the formation of chimeric chromosomes (Fig. S6). Of the 25 chromosomes, 8 were assembled as chimeric, retaining large haplotype-specific blocks, rather than creating a coherent collapsed assembly. This issue stems from significant structural discrepancies and low-sequence collinearity between the haplotypes, which led the software to incorrectly merge the haplotypes instead of generating a collapsed genome.

**Table 1:** Summary statistics of genome assembly and annotation for *A. comosus* var. *comosus* cultivar ‘Smooth Cayenne’ F180.

Category	HA (25 chr)	HB (25 chr)	Whole genome (50 chr)
Assembly			
Cumulative size (bp)	414.01 Mb	412.80 Mb	826.81 Mb
N50 (bp)	16.52 Mb	16.86 Mb	16.83 Mb
GC content (%)	39.15	39.06	39.10
Hi-C scaffolds	25	25	50
Complete BUSCO (%) (embryophyta_odb10, *n* = 1614)	99.20	99.40	99.50
LAI	24.82	23.23	–
QV	65.21	64.94	65.07
Gaps (count)	65	77	142
Telomeres detected	47/50	47/50	94/100
Centromeres detected	25/25	25/25	50/50
Annotation			
PCGs	20 280	20 248	40 528
Mean gene length (bp)	4593	4598	–
Mean CDS length (bp)	1341	1349	–
Average exon number per gene	6.0	6.0	–
Complete BUSCO for proteins (%) (embryophyta_odb10, *n* = 1614)	99.10	99.00	–

Assembly statistics are reported for haplotypes A (HA) and B (HB) separately, and for the combined diploid genome (50 chromosomes). Values include cumulative assembly size, contiguity (N50), GC content, scaffold number, BUSCO completeness (embryophyta_odb10, *n* = 1614), LAI, quality value (QV), gap counts, and detection of telomeres and centromeres. Annotation statistics include the number of PCGs, gene length metrics, and BUSCO completeness for predicted proteins (embryophyta_odb10, *n* = 1614). Detailed mapping statistics, transcriptome alignment rates, and functional annotation breakdowns are provided in Supplementary Table S5.

Different approaches were used to evaluate the quality of the assembly and phasing of HA and HB. The overall mapping rates for HiFi, stLFR, and RNA-seq reads from various tissues were 99.62%, 97.86%, and 83.11%–95.29%, respectively (Table S5). We estimated the base-level accuracy using Merqury based on *k*-mer spectra, resulting in a quality value (QV) of 65.21 for HA and 64.94 for HB, respectively, with a base error rate of <10^−6^. BUSCO assessment revealed that HA, HB, and the whole genome of F180 contained 99.2%, 99.4%, and 99.5% complete core orthologous genes, respectively, based on the highly conserved core embryophyte gene set (*N* = 1614) and the default gene predictor MetaEuk (Fig. 1d, Fig. S7, Table S6), ranking F180 among the top 3 pineapple genomes (Fig. S8). Particularly, the BUSCO value for F180 HB improved from 98.3% to 99.4% following several rounds of manual curation. The long terminal repeats (LTR) assembly indices (LAI) were evaluated at 24.82 for HA and 23.23 for HB, both surpassing the gold quality assembly threshold of ‘LAI > 20’ [28] and superior to those of most other pineapple genomes (Fig. 1e). Together, these quality assessments validate that we have assembled a nearly complete, T2T haplotype-resolved genome for F180 with high accuracy, contiguity, and fully resolved centromeres.

### Repetitive sequence and gene annotation

Genome-wide repeat annotation revealed that interspersed elements dominate the F180 genome (Table S7), comprising 266.49 Mb (61.51%) in HA and 264.46 Mb (62.20%) in HB, with a large proportion remaining unclassified (39.38% and 40.54%, respectively). LTR retrotransposons (LTR-RTs), the major category of retroelements (Class I), were the prevalent interspersed repeats, contributing 20.27% of HA and 19.25% of HB. Within this category, Gypsy/DIRS1 were the most predominant type (15.43%–16.18%), followed by Ty1/Copia (3.66%–3.71%) (Fig. 1c, Table S7). In contrast, DNA transposons (Class II) represented only a minor fraction (1.05% in HA and 1.36% in HB). Aside from interspersed repeats, small RNA repeats, simple repeats, and low-complexity repeats constituted only 0.28%–1.89% of the genome. Overall, a cumulative length of 281.50 Mb (64.97%) in HA and 277.76 Mb (65.32%) in HB were masked by RepeatMasker prior to gene annotation.

We predicted 20 280 and 20 248 high-confidence protein-coding genes (PCGs), corresponding to 24 280 and 24 283 transcripts or coding sequences (CDS) for HA and HB, respectively (Table S5). The PCGs spanned 93.16 Mb for HA and 93.12 Mb for HB, with average lengths of 4593 and 4598 bp for PCGs, and 1341 bp and 1349 bp for CDSs. Each gene harboured an average of 6.0 exons, with exon lengths averaging 222 bp for HA and 223 bp for HB. Functional annotation against the Nr, Gene Ontology (GO), EggNOG, KEGG, and SwissProt databases assigned 97.86% of PCGs to at least one database, with ~26% annotated by all five databases (Table S5, Fig. S9). The complete BUSCO values for PCGs were 99.1%, 99.0%, and 99.5% for HA, HB, and the whole genome, respectively, indicating high gene set completeness. The F180 HA and HB assemblies showed strong gene-level collinearity with F153 and the gap-free pineapple reference genome GP [27] (Fig. S10).

A total of 2919 and 2962 ribosomal RNAs (rRNAs) were identified for HA and HB, with combined lengths of 3.49 and 2.51 Mb, respectively (Table S8). At the chromosome level, 5S rRNA was the most abundant, distributed across 13 chromosomes with a large cluster on chromosome 18 (Chr18), while 28S and 5.8S rRNAs were the least common, present only on Chr25 in HA and on Chr13 and Chr25 in HB. Additionally, 1032 and 870 transfer RNAs (tRNAs) were predicted in HA and HB, with total lengths of 75.82 and 63.71 kb, respectively (Table S9).

### Structural variations and three large inversions

**Figure 2:**
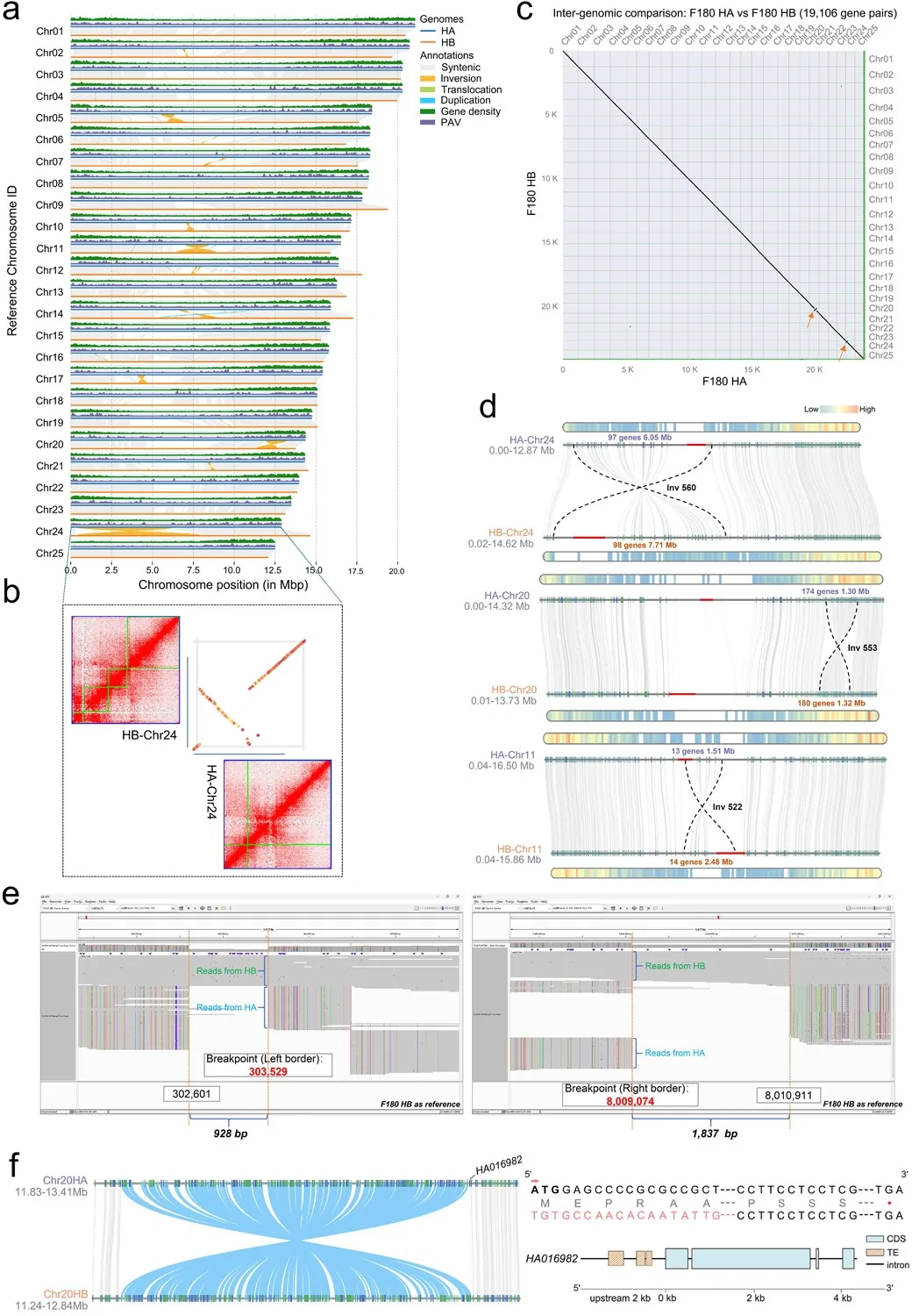
Whole-genome syntenic comparisons within ‘Smooth Cayenne’ F180. (a) PAV landscape and nucleotide-level collinearity between F180 HA and HB. The first and second tracks represent gene density and PAV distribution, calculated using a 50-kb sliding window. (b) The Hi-C interaction signal along Chr24 for HA and HB presents high consistency between neighbouring blocks. Each Hi-C interaction heatmap box represents a chromosome, and the small boxes within it represent supercontigs. (c) Synteny dot plot of whole-genome PCGs between HA and HB generated by MCScanX [29]. Two inversions located at Chr20 and Chr24 were visible. (d) Synteny of PCGs of Chr11, Chr20, and Chr24 between two subgenomes. The blue and green rectangle frames indicate genes transcribed in clockwise and counterclockwise directions, while the grey bars denote the intergenic regions. The centromere positions are marked by red bars on the axes. (e) PacBio read mapping validates the largest insertion (6.0~7.7 Mb) on Chr24. Reads from HB, but not HA, span the two breakpoints (at 303529 and 8 009 074 bp) of this Chr24 inversion on the F180 HB reference. Notably, no HA reads aligned to the 928 bp upstream of the left border or 1837 bp downstream of the right border in the HB reference, both of which were annotated as nonfunctional regions. (f) The hemizygous gene *HA016982.1* spans the inversion breakpoint on Chr20. The gene retains an intact coding sequence in HA, while its HB allele is truncated by an ATG deletion. Its 2-kb promoter region harbours three TE insertions.

Synteny analysis revealed 489 syntenic regions covering ~310 Mb of each haplotype, with a total of 8851 SVs detected between the two haplotypes, including 74 inversions (0.8%), 750 translocations (8.5%), and 1129 duplications (12.8%), and 6898 presence/absence variations (PAVs; 77.9%) (Fig. 2a, Table S10). The PAVs comprised 3391 large insertions (1.64 Mb) and 3507 large deletions (2.06 Mb), which represent a major source of sequence divergence between haplotypes. Most syntenic blocks exhibited high collinearity between haplotypes, though 1435 HA and 2092 HB regions were unaligned. Allelic sequence comparisons between haplotypes revealed a multitude of sequence variations, comprising 1 538 444 SNPs, 112 393 small insertions, 113 639 small deletions, 93 copy gains, 106 copy losses, 18 083 highly diverged sequences, and 20 tandem repeats.

Regarding inversions between the two haplotypes, the combined length of all 74 inversions was 12.27 Mb (2.96%) in HA and 15.66 Mb (3.79%) in HB, with average lengths of 165.8 and 211.7 kb, respectively. The inversion sizes varied among chromosomes, ranging from 219 bp to 6 Mb in HA and 219 bp to 7 Mb in HB (Tables S11 and S12, Fig. S11). The three largest inversions (>1 Mb), Inv560, Inv522, and Inv553, were located on Chr24, Chr11, and Chr20, respectively, with size ranges of 6.05–7.71, 1.51–2.48, and 1.30–1.32 Mb (HA vs HB). The Hi-C contact matrices confirmed the presence of genuine inversions between these homologous chromosomes (Fig. 2b, Figs S12 and S13). To assess how these structural rearrangements affected gene organization, we further examined synteny at the PCG level. Much higher syntenic relationships among genes between the two haplotypes were observed compared to the nucleotide level (Figs 2c and S14). Genes on most chromosomes except Chr11, Chr20, and Chr24 were largely conserved (Fig. 2d, Tables S12 and S13). Although the Chr24 inversion is the largest structurally, it contained fewer genes (97 in HA and 98 in HB) than the Chr20 inversion (174 in HA and 180 in HB), which spanned only one-fifth of its physical length. Likewise, the Chr11 inversion, despite being the second largest by nucleotide length, encompassed only 13–14 genes and was therefore barely visible at the gene-collinearity scale.

To further validate the authenticity of the largest inversion (Inv560) on Chr24, the entire dataset of F180 PacBio long reads was aligned to the F180 HB assembly (Fig. 2e). Two breakpoints (at 303 529 and 8 009 074 bp) of Inv560 in HB were identified. PacBio reads from the HB haplotype fully spanned both breakpoints, covering the entire inversion and its flanking sequences. In contrast, no HA reads spanned both breakpoints, with alignments exclusively detected up to 928 bp upstream of the left border and extending to 1837 bp downstream of the right border in the HB reference. These border regions were annotated as nonfunctional (gene desert) areas, indicating no gene disruption by the breakpoints on Chr24 (Table S13). Notably, Inv560 encapsulated the entire centromere adjacent to a breakpoint in both haplotypes, rendering Chr24 in HB the most acrocentric chromosome (Fig. S5). Similarly, the presence of Inv553 on Chr20 was confirmed by PacBio long-read alignments (Fig. S15).

Representatives of all available pineapple genomes (Table S14) were aligned against HA chromosomes 1 to 25 (Fig. S16). Dot plots of these alignments revealed that the 1.3-Mb inversion (Inv553) in Chr20 of HB was unique compared to the other genomes. However, haplotype 1 (H1) of the domesticated Queen variety ‘Ba Li’ (‘BL’) potentially contains this inversion nested inside another inversion (Fig. S17). Obvious counterparts of the 6-Mb inversion (Inv560) of Chr24 were not detected in any other pineapple genomes (Fig. S16). However, several rearrangements and smaller inversions were detected in its vicinity (Fig. S17). Notably, a large deletion in ‘BL’ H1 included the centromere (Fig. S18), thereby indicating incompleteness of its assembly. A counterpart of the second largest inversion in Chr11 HA was only found in ‘BL’ H2 (Fig. S16).

Compared to the F180 Chr17 haplotypes, ‘BL’ appeared to have a large chromosome arm deletion and duplications in haplotypes H1 and H2, respectively (Fig. S16). A large chromosomal arm inversion was also obvious in both haplotypes of the phased ‘MD-2’ genome compared to the F180 Chr25 haplotypes, among other notable differences that similarly occurred in the original collapsed F153 genome. Levels of collinearity between F180 and the haplotype-resolved chromosomes of ‘Yugafu’ were generally acceptable, with exception of Chr5 and Chr25 and several heterozygous inversions. The collinearity of F180 with the haplotype-resolved genomes of *erectifolius* (‘LY’) and *microstachys* (‘YLL’) varieties was generally high, with some obvious regions of low homology. In contrast, available *bracteatus* collapsed assemblies (‘CB5’, ‘GL1’) appeared to possess numerous structural rearrangements that did not have obvious counterparts in other genomes.

### Allelic gene variations and inversion-specific comparison

**Figure 3:**
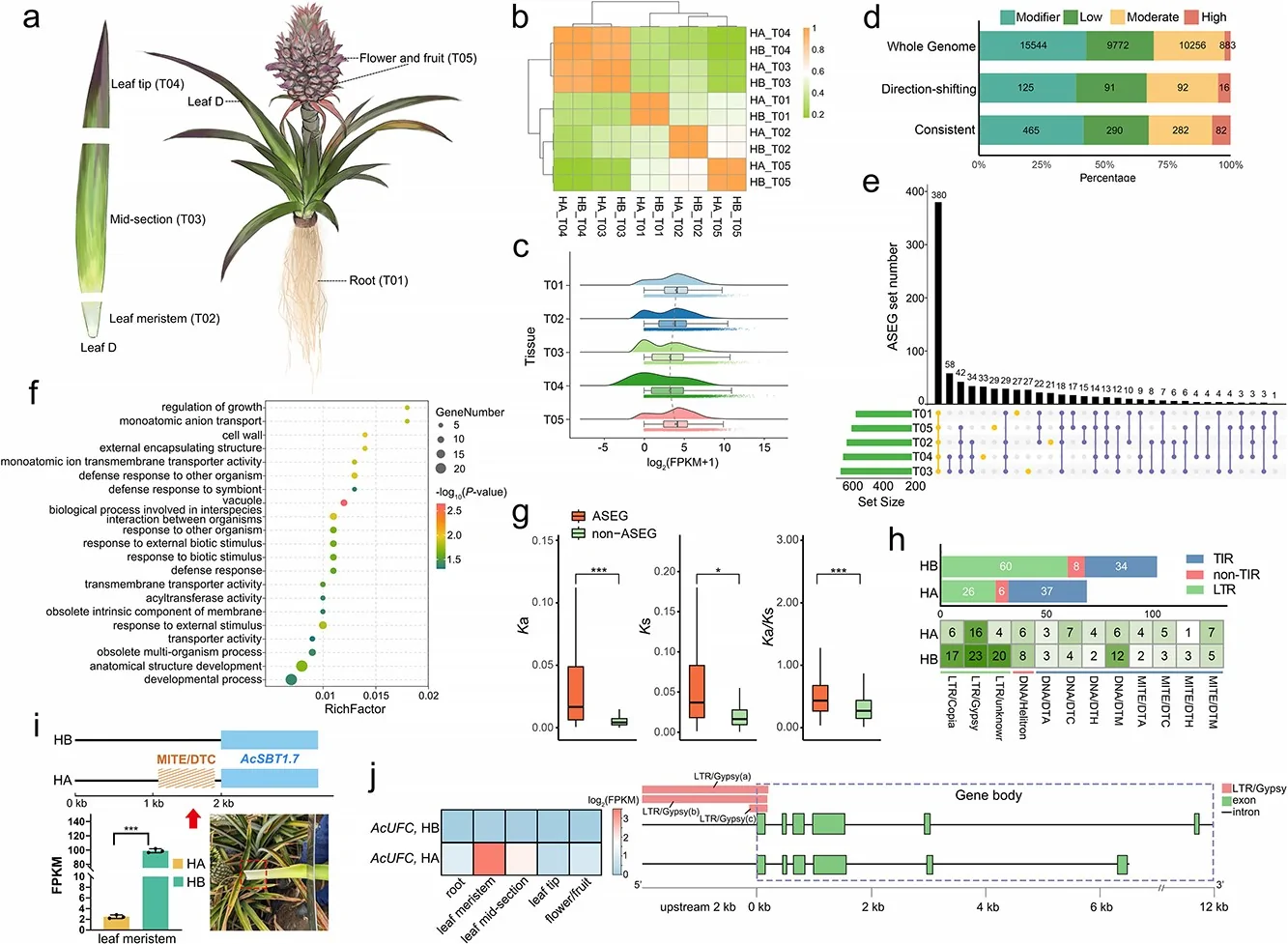
Functional and evolutionary dynamics of allele-specific expressed genes (ASEGs) in pineapple subgenomes. (a) Five tissues include roots (RNA-seq T01), meristem (T02), mid-range (T03), leaf tip of mature leaf (T04), and combined flower and fruit (T05). (b) Correlation analysis and clustering of the expression of alleles in five tissues. Each data point represents the mean of three biological replicates for HA or HB in a given tissue. (c) Raincloud plot of log_2_(FPKM + 1) values of genes across five tissues. The average expression level of each tissue is connected by a grey dashed line. Similar expression trends were observed for subgenomes. (d) The percentage stacked bar chart of pineapple ASEGs with high, moderate, low, and modifier impact variants. Numbers denote genes with ≥1 variant, and genes may occur in multiple categories. ‘Whole genome’ denotes the genome-wide background for comparison. ‘Direction-shifting’ ASEGs are defined as those showing a reversal of allelic imbalance between tissues. ‘Consistent’ ASEGs maintain stable imbalance towards the same allele across all five tissues. (e) The tissue-specific distribution of ASEGs detected in five tissues in the pineapple subgenomes. Yellow dots represent ASEGs that are allele specific in only one tissue or in all tissues. (f) GO enrichment analysis of tissue-specific ASEGs across the five sampled tissues. The *y*-axis shows significantly enriched GO terms, while the *x*-axis represents the RichFactor. Dot size indicates the number of ASEGs associated with each GO term, and colour corresponds to the statistical significance (−log_10_  *P* value). (g) Comparison of *Ka*, *Ks*, and *Ka*/*Ks* between ASEGs and non-ASEGs by independent *t* test. ^*^P < 0.5; ^***^P < 0.001. (h) The distribution of TE numbers and types between HA and HB. Darker green indicates higher TE abundance. (i) The ASEG (*AcSBT1.7*) with a specific intact MITE/DTC insertion in the upstream 2-kb region. The bar plot shows the FPKM of the gene in leaf meristem. (j) An example of an ASEG (*AcUFC*) with LTR/Gypsy insertions in promoter and gene regions. Left: expression heatmap of *AcUFC* in HA and HB across five tissues. Right: schematic showing the LTR/Gypsy insertions.

In total, 19 058 allelic gene pairs (19 153 one-to-one gene pairs) were identified between HA and HB, comprising 19 084 genes from HA and 19 065 from HB (Tables S15 and S16, Fig. S19). Most homologous alleles exhibited a one-to-one correspondence, while a few displayed one-to-multiple or multiple-to-multiple relationships. Additionally, 1196 and 1183 genes were identified as unique to HA and HB, respectively. The *Ka*/*Ks* ratios for allelic gene pairs were generally less than one (95.79%; mean = 0.32), whereas positively selected genes (*Ka*/*Ks* > 1) were significantly enriched in defence-related biological processes, particularly responses to fungi and other organisms (Fig. S20). RNA-seq analysis of HA-HB allelic genes revealed clear tissue-specific expression, characterized by strong within-tissue clustering (Fig. 3a and b). Overall, these genes showed higher expression in roots and flowers/fruits than in leaves, and among the three distinct leaf tissues, the meristem exhibited the highest average expression (Fig. 3c).

We examined allelic gene PAVs within and around inversions, as well as any resulting positional shifts. Overall, a total of 330 and 337 genes were located within HA and HB inversions, respectively (Table S13). Specifically, 303 allelic gene pairs were present within inversions between haplotypes, and 21 HA-specific and 29 HB-specific genes were found. Moreover, within the inversions of HA and HB, six and five genes, respectively, were identified with their allelic counterparts located outside the inversion regions.

Regarding inversion breakpoints, 11 and 10 genes were found to span the breakpoints in HA and HB, respectively. This set included eight allelic gene pairs and two haplotype-specific breakpoint-spanning genes whose allelic counterparts did not cross the breakpoints (Tables S13 and S17). No genes were detected near the breakpoints of the largest and second-largest inversions on Chr24 and Chr11, likely due to both inversions overlapping the centromeric regions (Fig. 2d). By contrast, a single gene in HA (*HA016982.1*), which spans a breakpoint on Chr20 and encodes a putative protein (1184 aa) with 97.5% identity to SMG8 (a nonsense-mediated mRNA decay factor) [30], was disrupted by the inversion breakpoint on Chr20 in HB (Fig. 2f). Further manual checks and read mapping indicated that the HA allele contains a complete coding sequence with intact ATG start and TGA stop codons, while the corresponding sequence in HB was incomplete due to the absence of the ATG start codon. This hemizygous gene exhibited reduced expression, with three transposable element (TE) insertions identified within its 2 kb upstream region.

### Allele-specific expression and inversion-region expression

We comprehensively identified allele-specific expression genes (ASEGs) from HA and HB alleles using RNA-seq data collected from five tissues per sample. A comparison of 19 153 allele-defined gene pairs (one-to-one) revealed that 863 pairs (4.28%) showed significant ASE (|log_2_FoldChange| > 1 and *P* < 0.05) in at least one tissue (Table S18). These ASEGs were further classified into 713 consistent and 150 direction-shifting ASEGs (Tables S19 and S20 and Figs S21–S22). The percentage of high-impact variants in consistent ASEGs was much larger than in direction-shifting ASEGs or the genome-wide baseline (Fig. 3d). Tissue-specific ASE analysis showed that the majority of ASEGs (44.03%) were expressed across all five tissues, while smaller proportions were specific to leaf tips (3.82%), flowers/fruits (3.36%), leaf mid-sections (3.12%), roots (3.12%), and leaf meristems (2.43%) (Fig. 3e). The ASEGs expressed in all tissues were mainly enriched in biological processes related to substance transport and energy metabolism (Fig. S23), while tissue-specific ASEGs were primarily associated with defence responses and tissue development (Fig. 3f). ASEGs also exhibited significantly higher *Ka* and *Ka*/*Ks* ratios than non-ASEGs, indicating their rapid evolutionary rate (Fig. 3g). Despite extensive inversions in the pineapple genome, ASEGs within inverted regions exhibited no significant expression divergence from collinear regions across tissues (*t* test, *P* > 0.05) (Fig. S24).

TE insertions were detected within the 2-kb upstream promoter regions of 86 out of the 863 ASEG pairs analysed (HA: *n* = 45; HB: *n* = 53; both: *n* = 12) (Table S21). The abundance and types of TEs differed between haplotypes (Fig. 3h). A potential association between haplotype-specific TE insertions and allelic expression bias was observed at several loci. A representative example involves the *AcSBT1.7* gene, which encodes a subtilisin-like protease known to regulate plant developmental processes, defence, and stress responses [31]. In HA, an intact MITE/DTC insertion within the 2-kb upstream promoter region coincided with a ~30-fold suppression of *AcSBT1.7* transcript levels in leaf meristem tissue relative to HB (Fig. 3i). Similarly, the *AcUFC* gene, a homologue of *Arabidopsis* UPSTREAM OF FLOWERING LOCUS C (*FLC*), exhibited HA-specific allelic dominance in leaf tissues (Fig. 3j). Notably, this regulatory locus interacts with *FLC* and *DFC* (DOWNSTREAM OF *FLC*) to mediate vernalization-related signalling pathways in *Arabidopsis* [32]. Three LTR/Gypsy retrotransposons were found exclusively in the promoter and coding regions of the HB allele.

### Impacts of reference quality and inversions on interchromosomal linkage and recombination patterns

**Figure 4:**
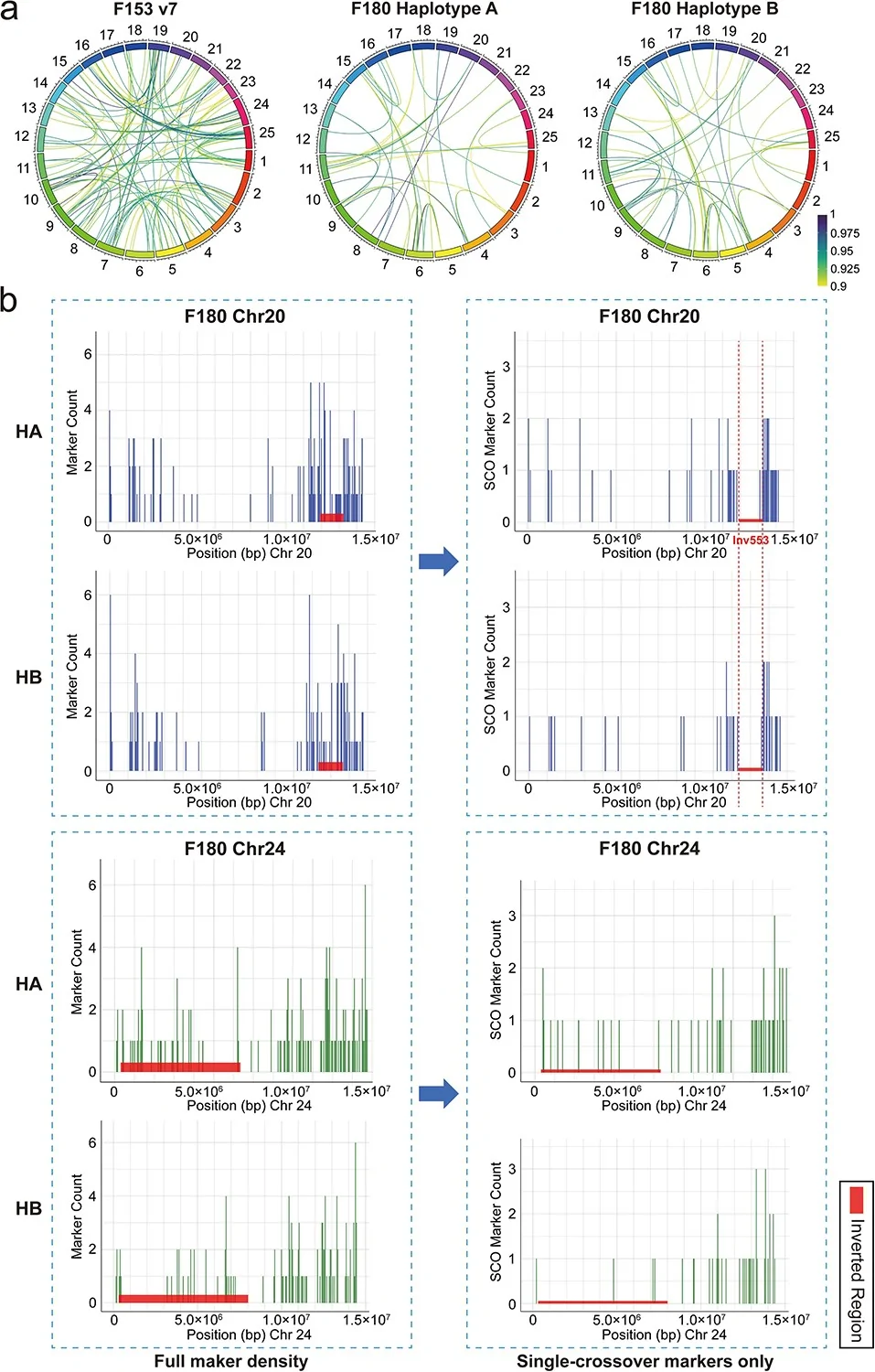
Genome assembly quality and inversion-associated recombination patterns in ‘Smooth Cayenne’ F180. (a) Interchromosomal linkage disequilibrium (LD; *R*^2^ **≥** 0.90) for 11 879 DArTseq™ loci mapped to three references: the collapsed ‘Smooth Cayenne’ F153 genome (left) and the two phased F180 haplotypes (HA and HB). Each line joins a marker pair located on different chromosomes; line thickness reflects the number of high-LD links. (b) Full (left panels) versus SCO (right panels) marker distributions in 50-kb bins across Chr20 and Chr24 for HA and HB. Horizontal bars indicate two large inversions: paracentric Inv553 on Chr20 and pericentric Inv560 on Chr24. Vertical bars represent marker densities on Chr20 and Chr24, respectively. All panels show data (full makers or SCO events) pooled from all 374 progeny. Near absence of SCO markers within Inv553 indicates strong recombination suppression, whereas the reduced SCO signals within Inv560 suggest partial suppression. A single SCO near the Inv553 boundary represents a minor positional outlier and does not alter the absence of crossovers within the inversion.

Pairs of DArTseq™ markers (*n* = 11 879) whose alleles are highly correlated (*R*^2^ ≥ 0.90) and lie on different chromosomes are plotted in Fig. 4a. When the markers were aligned to the short-read, collapsed ‘Smooth Cayenne’ F153 assembly [23], large numbers of interchromosomal linkages (241 unique marker pairs) were apparent. Realigning the identical marker set to the long-read, haplotype-resolved F180 assembly (HA and HB) significantly reduced the number of interchromosomal linkages to only 81 and 97 pairs, respectively. F153 contained ~1.94-fold more duplicated marker binding sites than F180, a discrepancy most likely attributable to assembly artefacts (Table S22). It is also noteworthy that whole-genome alignments with ‘MD-2’ required extensive filtering to remove spurious alignments and local alignments with DArT markers revealing levels of sequence duplication almost 6-fold higher in ‘MD-2’ than in the F180.

**Figure 5:**
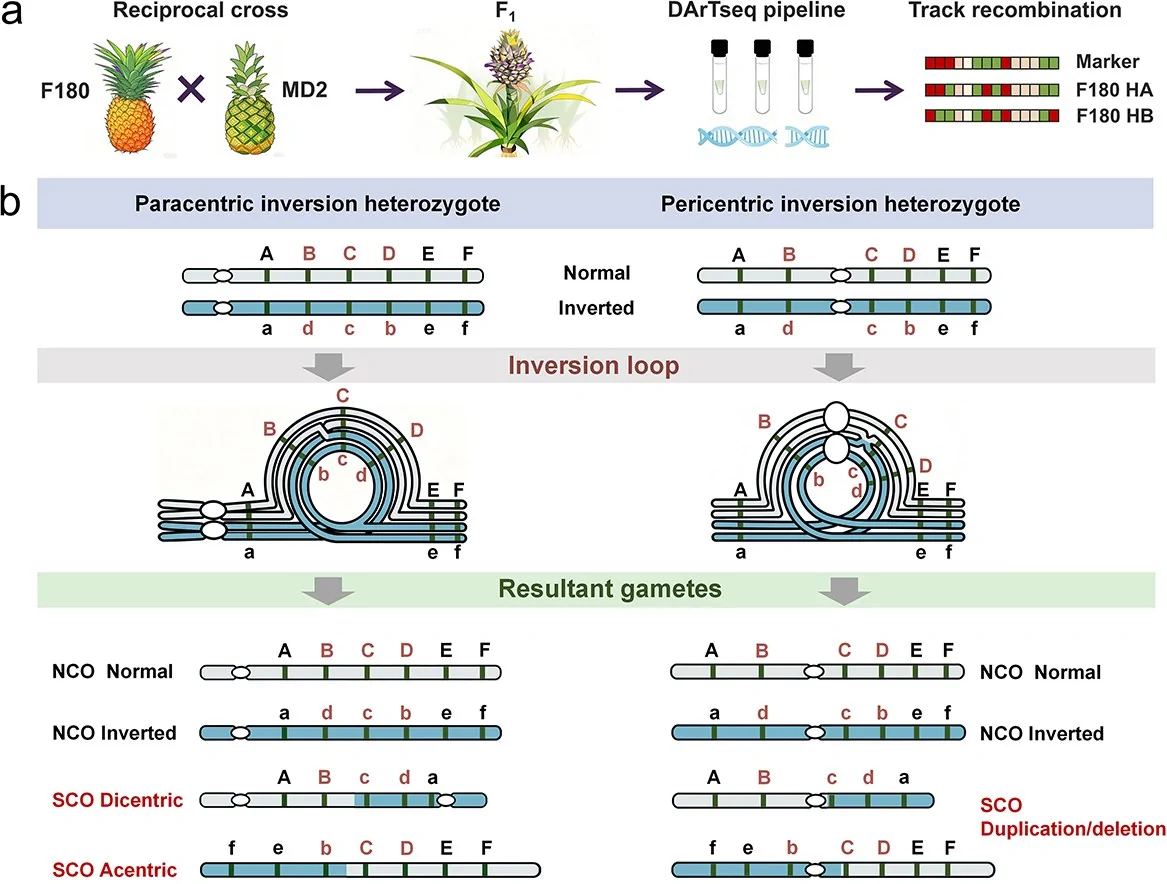
Mechanistic model of how inversions suppress recombination. (a) Strategy for tracking recombination with DArTseq genotyping in an F_1_ population derived from reciprocal cross between F180 and ‘MD-2’. (b) Meiotic consequences of paracentric (left) and pericentric (right) inversions in ‘Smooth Cayenne’ F180. In paracentric inversions (left, Chr20), SCOs produce dicentric bridges and acentric fragments, which cause gamete inviability and establish a strict recombination coldspot. In pericentric inversions (right, Chr24), although SCOs generate unbalanced gametes carrying segmental duplications or deletions, short-range DCOs (not depicted) can restore euploidy, permitting limited gene flow and attenuating the coldspot. NCO, noncrossover.

Using 669 high-quality DArTseq™ markers common to the F180 haplotypes HA and HB, recombination events in F180 were scored across 374 F₁ progeny. Across the 25 chromosomes, SCO frequencies averaged three events per chromosome in HA and HB, while DCO frequencies averaged one per chromosome for both haplotypes (Table 2). Chr3 and Chr4 exhibited the highest SCO activity in HA, whilst Chr3 had the highest activity in the HB haplotype. DCOs were most frequent on Chr3 and Chr13 (three DCOs each) in HA and HB. To further examine recombination patterns, we plotted marker density and SCO counts in 50-kb bins across chromosomes 20 and 24 for both haplotypes (Fig. 4b). In each case, the positions of inversions are marked by red bars along the *x*-axis. In HA, Inv553 on Chr20 displayed only one SCO, although it appears just within a boundary and is likely the result of minor positional inaccuracy. The size of the inversion and the lack of crossovers suggests strong recombination suppression (Table 3). A chi-squared pairwise comparison of proportions using SCO counts within the inverted region of the HA assembly and that of comparable length regions on either side of the inversion demonstrated a significant difference between the inverted region and the preceding region (Bonferroni-adjusted *P* = 0.012), and the inverted region and the following region (Bonferroni-adjusted *P* = 4.5e^−5^) (Table S23). The HB assembly showed a similar difference in SCO counts between the inversion and the preceding and following regions, with *P* values of 0.013 and 0.0023. By contrast, the Inv560 on Chr24 of HA contained 12 SCOs, suggesting that recombination can still proceed within this region, although the SCO density in the inverted region was markedly lower than the rest of the chromosome. There was no significant difference in SCO counts between the inversion and that of a similarly sized region at the opposite end of the chromosome. The HB assembly did, however, show a significant difference (Bonferroni-adjusted *P* = 0.0058) with only three SCOs within the Inv560. For comparison, full marker counts before recombination analysis demonstrated a moderate marker density across both chromosomes, including within inverted segments (Fig. 4b). Figure 5 models the meiotic consequences of an SCO event in inversion heterozygotes of F180, including inversion loop formation and the generation of nonviable or imbalanced gametes.

**Table 2:** Average numbers of SCO and DCO events per chromosome per individual, calculated from 374 F₁ progeny of reciprocal ‘Smooth Cayenne-F180’ × ‘MD-2’ crosses.

					Chromosome size			
Chromosome	Av SCO counts		Av DCO counts		HA		HB	
	HA	HB	HA	HB	cM	Mb	cM	Mb
1	3	4	1	1	74	21.1	71	20.5
2	2	2	1	0	69	20.7	65	19.7
3	5	6	3	3	65	20.3	64	20.2
4	5	4	2	2	67	20.3	66	20.0
5	4	4	2	2	62	18.4	57	17.6
6	3	2	1	0	62	18.3	56	16.8
7	2	2	0	0	58	18.3	56	17.6
8	3	3	1	1	65	18.2	64	18.2
9	4	4	2	1	60	17.8	66	19.4
10	3	3	1	1	57	17.2	56	17.1
11	3	3	1	1	53	16.5	51	15.9
12	3	3	1	1	51	16.4	56	17.8
13	4	4	3	3	53	16.3	55	16.9
14	3	2	1	0	52	15.9	57	17.3
15	3	2	1	0	53	15.8	50	15.3
16	3	4	1	2	52	15.8	50	15.5
17	2	2	1	0	50	15.4	49	15.0
18	2	2	1	0	50	15.1	54	15.1
19	2	2	0	0	48	14.8	49	15.1
20	2	2	0	0	48	14.4	50	13.8
21	2	2	1	0	47	14.3	48	14.5
22	2	2	0	0	47	14.0	48	13.9
23	1	2	0	0	41	13.5	40	13.1
24	3	2	1	1	42	12.9	48	14.6
25	1	1	0	0	17	12.5	14	12.1
*Av/Total*	*3*	*3*	*1*	*1*	*1344*	*414.0*	*1337*	*412.8*

Recombination was inferred independently for markers mapped to each phased F180 haplotype, yielding per-chromosome HA and HB estimates. Values are means per progeny per chromosome; haplotype-specific chromosome sizes (Mb) are provided for context.

**Table 3:** DCO events composed of SCOs detected within or near the large inversions on chromosomes 20 (Inv553) and 24 (Inv560) in haplotype A (HA) of ‘Smooth Cayenne’ F180.

Haplotype	Chr	Position 1		Position 2		Distance (cM)
*HA: Inv553: 20*		*11 928 447*		*13 232 671*		
HA	20	10 350 897	Out	13 296 283	Out	9.8
		10 350 897	Out	13 138 073	Out	9.3
		11 843 718	Out	13 846 945	Out	6.7
*HA: Inv560: 24*		*298 388*		*6 348 311*		
HA	24	323 091	In	6 935 708	Out	12.4
		450 224	In	3 488 511	In	10.1
		3 488 511	In	7 272 635	Out	12.6
		3 876 767	In	7 272 635	Out	11.4
		6 270 022	In	9 423 969	Out	10.5
		6 270 022	In	9 533 575	Out	10.9

Positions are given in base pairs, with genetic distances in centimorgans (cM). ‘In’ and ‘Out’ indicate whether markers fall inside or outside the inversion boundaries. Recombination in F180 was tracked using the haploid QTL2 model with F180 (A) versus ‘MD-2’ (B) allele coding.

### Haplotype-dynamic NLR repertoires in F180

**Figure 6:**
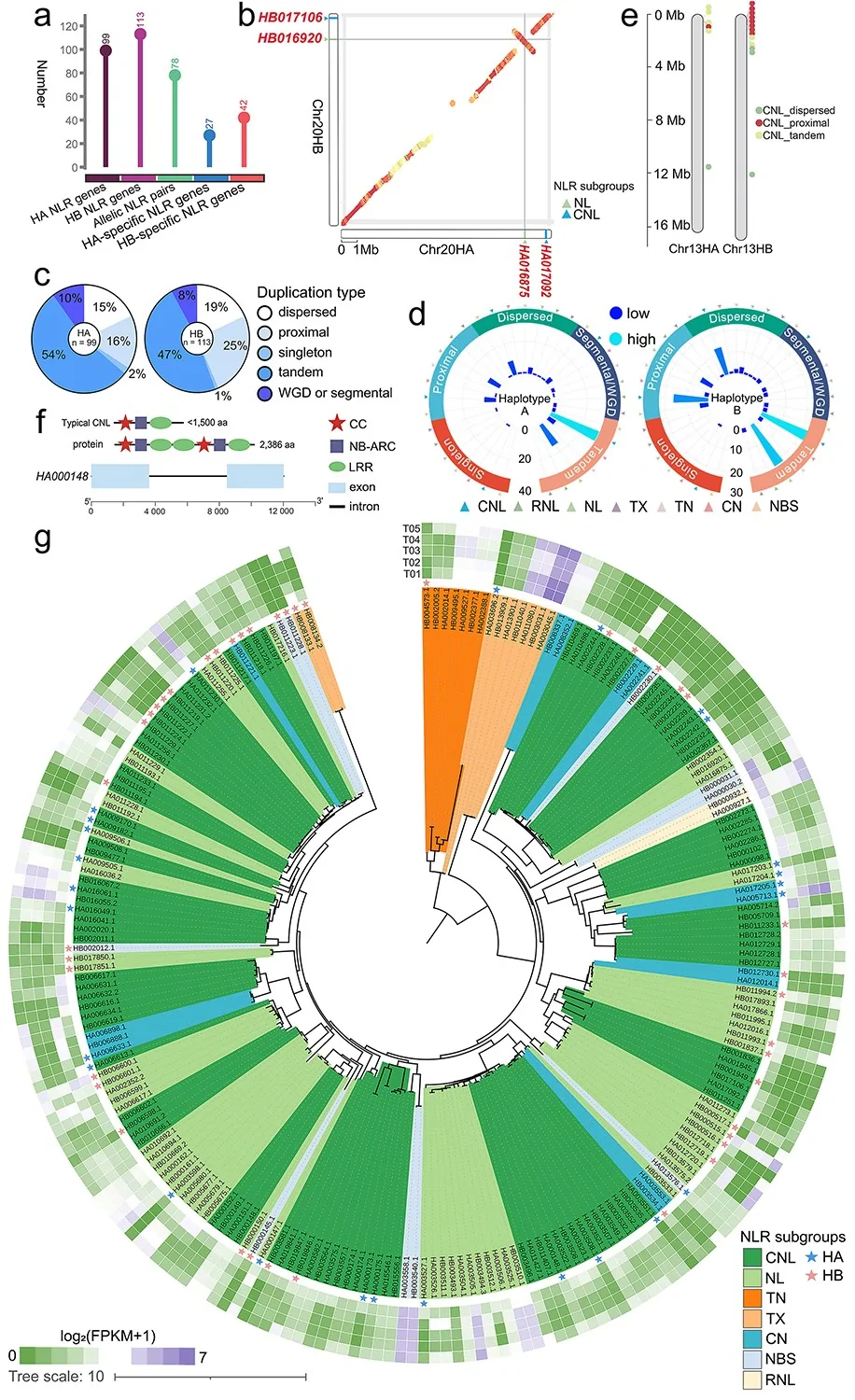
Genomic architecture and functional diversification of NLR genes in pineapple subgenomes. (a) The lollipop plot showing the distribution of NLR genes between two haplotypes. (b) Chromosomal locations of NLR genes on Chr20. (c) Pie charts of the proportions of different duplication types in subgenomes. (d) Distribution of NLR genes across duplication events. The outer ring represents five duplication types, while the inner ring indicates the abundance of NLR subgroups. (e) The haplotype-specific CNL expansion on Chr13HB. Three duplication events are displayed: dispersed, proximal, and tandem. (f) Schematic representation of a representative CNL protein and its gene structure. The scale bar indicates genomic positions in base pairs. CC: coiled-coil; NB-ARC: nucleotide-binding adaptor shared by APAF-1, R proteins, and CED-4; LRR: leucine-rich repeat. (g) Raincloud plot showing log_2_(FPKM + 1) values of NLR genes in HA (left) and HB (right) across five tissues. (h) Phylogenetic tree of 212 NLR protein sequences from the two subgenomes, constructed using IQ-TREE with 1000 bootstrap replicates. The heatmap shows relative expression levels across five tissues. Stars indicate HA- and HB-specific NLR genes.

To explore the potential disease resistance-related NLR genes in F180, we identified 99 and 113 NLR genes in HA and HB, respectively (Tables S24 and S25). These NLRs were classified into seven subgroups, which were distinguished based on the presence or absence of TIR, CC, NBS, and RPW8 domains. On average, CNL was the most prevalent NLR subgroup, followed by NL, while TX and RNL were the least common in both haplotypes (Fig. S25, Table S26). NLR genes were unevenly distributed across haplotypes, with Chr7, Chr17, Chr23, and Chr24 lacking NLRs (Fig. S26). The number of NLR genes on each chromosome varied by up to 12 genes (on Chr13) between haplotypes. HA uniquely harboured two NLRs on Chr10, while HB retained one on Chr5. Most of the pineapple NLR genes resided in clusters and were located near telomeric regions (Fig. S27). A total of 78 NLR allelic gene pairs were identified, with 27 and 42 NLR genes specifically detected in the HA and HB haplotypes, respectively (Fig. 6a, Tables S27 and S28), shedding light on the dynamic nature of R genes [33]. An NLR allelic gene pair (*HA016875–HB016920*) was exclusively identified within the Inv553 on Chr20 (Fig. 6b).

Analysis of genomic duplication dynamics delineated distinct evolutionary trajectories of NLR expansion mechanisms in both haplotypes, with tandem, proximal, and dispersed duplications serving as principal drivers (Fig. 6c). Furthermore, divergence in NLR subgroup expansion mechanisms was associated with distinct duplication modes (Fig. 6d). CNL and NL subgroups mainly expanded through tandem and proximal duplication events, respectively. Interestingly, NLR duplication patterns exhibited marked heterogeneity between haplotypes. For instance, proximal duplications in HB generated twice as many CNL subgroups as those in HA, while a parallel trend was observed for the NL subgroup amplified through tandem duplication. Specifically, proximal duplication on Chr13 HB accounted for the haplotype-specific CNL expansion (Fig. 6e). Diverging from canonical CNL architecture, the atypically large CNL gene *HA000148* (~12 kb) on Chr1 HA possessed a dual CC-NB-ARC domain (Fig. 6f), potentially enabling multipathogen effector surveillance [34]. No structural orthologues were identified in HB.

Consistent with the genome-wide expression pattern of HA-HB allelic gene pairs, NLR genes exhibited elevated transcriptional activity in roots and reproductive organs (flowers/fruits) relative to leaves across both subgenomes (Fig. S28). The phylogenetic analysis revealed complex evolutionary diversity and variation in subfamily expression levels (Fig. 6g). Allelic NLR gene pairs generally clustered together and displayed similar expression patterns. For example, TX-type genes (e.g. *HA003045–HB003031*) were highly expressed in all five tissues. No significant expression divergence was observed between HA- and HB-specific NLR genes (*P* > 0.05) (Fig. S29). Collectively, the majority of HA/HB-specific NLR genes exhibited low transcriptional activity (FPKM < 10; Fig. S30). However, notable exceptions included *HA005713* and *HA011230*, which exhibited exceptionally high expression in leaves, and *HB000517* and *HB012719*, with elevated expression in roots and leaf tips. These genes warrant further functional characterization to dissect their roles in tissue-specific immune responses.

## Discussion

The haplotype-resolved genome assembly of the pre-Columbian pineapple cultivar ‘Smooth Cayenne’ presented here marks a significant advancement over previous assemblies [23], addressing the limitations of haploid representations in highly heterozygous genomes [1]. The results from our *de novo* assembly (Section *De novo* haplotype-resolved genome assembly of Smooth Cayenne) demonstrate the power of integrating PacBio HiFi long-read sequencing (47× coverage) and Hi-C long-range data (40× coverage) [4], producing a high-quality phased genome consisting of 50 pseudo-chromosomes (433.3 Mb for HA, 425.2 Mb for HB) with exceptional contiguity (N50 of 16.38–16.86 Mb) and completeness (BUSCO >99%, LAI > 23). Here, we were able to construct complete haplotype-resolved assemblies for all 25 chromosomes, including the identification of all 50 centromeres and telomeric repeats on 22 out of 25 chromosomes per haplotype, further underscoring the robustness of this resource. Repetitive elements, predominantly LTR retrotransposons (20.27% in HA, 19.25% in HB), constitute ~62% of the genome, substantially more than the collapsed F153 assembly (44%) [23]. The prediction of ~20 248–20 280 (HA and HB, respectively) high-confidence PCGs, with 97.86% functionally annotated and BUSCO completeness of 99.0%–99.5%, provides a robust foundation for genetic studies and breeding applications. The high collinearity with the recently released gap-free pineapple reference genome composite [27] validates the accuracy of our gene annotations, enabling the precise identification of trait-associated loci to address challenges like flowering asynchrony and pathogen susceptibility [35–37]. The presence of 2919–2962 rRNAs and 870–1032 tRNAs, with distinct chromosomal distributions, offers additional targets for studying noncoding RNA functions in pineapple adaptation, fruit ripening, and development [38, 39].

Attempts to generate a collapsed genome failed due to significant haplotype divergence and low sequence collinearity, underscoring the necessity of phased assemblies for capturing the full complexity of clonally propagated, highly heterozygous crops like pineapple. Our phased assembly resolved critical structural variations, including the large inversions on chromosomes 20 and 24, which would otherwise remain obscured in collapsed genomes through chimeric contig formation [18]. Allelic variations and inversion-specific comparisons reveal the extent of haplotype-specific diversity in ‘Smooth Cayenne’, contributing to the high genome-wide heterozygosity (1.57%) observed in this accession. Allelic diversity between the two haplotypes was substantial. For example, 1.5 million SNPs were identified between haplotypes. However, it is important to note that despite the clonal nature of pineapple cultivation likely occurring for thousands of years, in turn preserving heterozygosity, the initial domestication process of these varieties has greatly reduced the relative diversity of common varieties, i.e. ‘Queen’ and ‘Smooth Cayenne’, in comparison to related *Ananas* cultivars and species [22]. The identification of 19 058 shared allelic gene pairs, alongside 1196 HA-specific and 1183 HB-specific genes, highlights the genetic divergence preserved by clonal propagation. *Ka*/*Ks* analysis of these haplotype-specific genes displays the predominance of purifying selection (*Ka*/*Ks* ≤ 1), ensuring genomic stability. Genes under positive selection (*Ka*/*Ks* > 1), i.e. genes that display heightened ratios of nonsynonymous mutations, show clear selective trends towards photosynthetic and organelle-related processes and defence-related genes. The presence of a variety of defence-related genes suggests adaptive evolution against pathogens, particularly fungi-related genes, as fungi are the major pathogen type of cultivated pineapple [26, 40, 41]. There were also ~45 additional genes associated with defence response and the regulation of biological activity in HA, suggesting genes key to both defence and the regulation of biological processes, indicating the importance of haplotype-specific contributions to the resilience and agronomic performance of ‘Smooth Cayenne’. Haplotype-specific NLR gene repertoires, comprising 99 and 113 genes in HA and HB, respectively, exemplify this diversity, offering a dynamic resource for combating pineapple diseases.

Linkage analysis of the ‘Smooth Cayenne’ × ‘MD-2’ progeny reveals a consistent pattern in which crossovers cluster near telomeres and are scarce in centromeric regions [11]. Across 374 F₁ progeny, we observed a genome-wide mean of crossover (CO) events that falls squarely within the one-to-five CO per chromosome range reported for many crops [42] and illustrates classical CO interference, whereby the formation of one exchange suppresses nearby events [43]. A total of 1953 SVs (including 74 inversions, 750 translocations, and 1129 duplications) and extensive sequence variations (e.g. 1 538 444 SNPs) underscore the haplotype-specific differences between HA and HB. The three largest inversions, found on Chr24 (6.05–7.71 Mb), Chr11 (1.51–2.48 Mb), and Chr20 (1.30–1.32 Mb), which were validated by Hi-C matrices and PacBio read mapping, are particularly significant. These inversions, encompassing large segments of the F180 genome, have the potential to suppress recombination, preserving haplotype-specific gene blocks as single inheritance units [44, 45]. This has profound implications for breeding strategies, as it complicates the introgression of valuable traits. The two largest inversions in ‘Smooth Cayenne’ display contrasting crossover behaviour that does not fully align with expectations from their structural classifications. Inversion 553 on Chr20, which is paracentric, shows an absence of recombination. Instead, a higher density of SCOs occurs at either end (Fig. 4b). This suggests that recombination suppression within the inversion may reduce crossover interference and promote exchanges in adjacent regions, as has been observed in *Drosophila* [46]. In contrast, inversion 560 on Chr24, which is pericentric, shows limited but detectable recombination, with several SCOs and DCOs occurring within one half of the inversion. The relatively large size of this pericentric inversion may permit partial alignment between homologous chromosomes, allowing occasional recombination to occur despite centromere involvement [47]. Strong suppression is expected in both para- and pericentric inversions, with additional centromere-associated suppression in the latter. However, the extent of suppression varies with inversion size, breakpoint locations, and genomic context [12, 48], highlighting the complexity of inversion-specific recombination dynamics. From a breeding perspective, these patterns suggest that Inv553 on Chr20 may act as a true recombination coldspot, locking ~170 genes into a single haploblock that can be inherited intact, whereas Inv560 on Chr24 may permit limited gene flux through recombination or gene conversion. Here, we present a mechanistic model of how inversions suppress recombination in F180 (Fig. 5). In the paracentric inversion heterozygotes on Chr20 (1.3 Mb), SCOs produce abnormal dicentric and acentric chromosomes, leading to inviable gametes, effectively suppressing recombination and creating a strict recombination coldspot in the region. In contrast, although SCOs in the pericentric inversions on Chr24 (6.7 Mb) can yield unbalanced gametes, DCOs may restore euploidy, allowing limited recombination and weakening the coldspot. Such differences are critical for understanding how inversions shape haplotype inheritance, mutational load, and the potential for linkage drag versus allele fixation in breeding populations. The absence of these two major inversions in key commercial pineapple genomes, including ‘MD-2’ and ‘Queen’, as seen in Fig. S16, may stem from true varietal differences in ‘Queen’. Whereas for ‘MD-2’, it may reflect a somaclonal origin or differing parental contributions. Other explanations could be linked to Smooth Cayenne’s domestication from *A. comosus* var. *microstachys* or ancient hybridization events with *A. comosus* var. *bracteatus* and others [22, 23]. The collapsed ‘Smooth Cayenne’ F153 assembly likely masks these inversions by failing to fully resolve haplotypes, highlighting a technical limitation rather than a true difference from F180 and further demonstrating the necessity of haplotype-resolved assembly. Evidence also suggests the improper assembly of the phased ‘MD-2’ genome, which contains numerous artificial duplications. Conserved ASE across inverted and collinear regions suggests large-scale inversions in the pineapple genome may preserve local regulatory landscapes, potentially through intact chromatin topology or compensatory epigenetic mechanisms [49], allowing ASEGs within inverted regions to maintain expression stability across tissues. By determining the orientation and allele content of such regions in diverse parents and progeny, strategies can be devised to manipulate genetic linkage for important agronomic traits. Generating improved assemblies of other commercial and wild varieties and systematically identifying their SVs will be critical for enabling such approaches.

When the same 11 879 DArTseq loci used in the linkage analysis were mapped to the earlier collapsed F153 assembly [23], over 240 high-LD links joined nonhomologous chromosomes, likely an artefact of misplaced or merged contigs. After realignment to the phased HA and HB genomes, those spurious links dropped to less than 100, leaving a much-reduced set of more reliable long-range associations. For breeders, this reduction in ‘false LD’ translates directly into cleaner GWAS signals and more accurate genomic prediction models, especially where LD decay and haploblock analysis is conducted on a chromosome-by-chromosome basis [50]. The linkage results reinforce the practical value of using the haplotype-resolved F180 reference for downstream genetics, instead of non-T2T references. Taken together with the larger gene set (~20 250 per haplotype) and the ability to trace haplotype-specific disruptions such as the inversion-breakpoint gene on chromosome 20, the phased F180 resource offers a markedly higher quality resource than previous ‘Smooth Cayenne’ assemblies. QTL mapping for targets such as flowering asynchrony [35, 36], pathogen susceptibility [25, 40], and climatic resilience [51] should proceed with higher resolution and lower false-positive rates, accelerating marker-assisted and genomic-selection pipelines in pineapple breeding programmes [52].

Allele-specific expression plays a critical role in heterosis and adaptive trait regulation [53], which has led to its extensive study in multiple fruit crops [33, 54–56]. Our study presents the first comprehensive analysis of ASE in pineapple across five distinct tissues (Section Allele-specific expression and inversion-region expression). The low prevalence of ASE in pineapple (4.28% of genes in at least one tissue) suggests that homologous alleles are predominantly expressed in a coordinated manner, with transcriptional balance maintained across most heterozygous loci. ASEGs can be grouped into two primary patterns: consistent ASEGs (stable allelic bias across tissues) and direction-shifting ASEGs (tissue-dependent reversal of allelic bias). These patterns align with the dominance and overdominance hypotheses in the genetic basis of heterosis, respectively [57–59]. Underlying this divergence, genomic variation annotation highlights that consistent ASEGs harboured a greater proportion of high-impact variants compared to direction-shifting ASEGs. The consistent allelic bias towards HA or HB probably reflects compensatory buffering against deleterious effects of high-impact variants (e.g. protein-truncating mutations) in the alternative allele [57, 60]. Additionally, the number of consistent ASEGs is much higher than that of direction-shifting ASEGs, mirroring dominance-biased allelic regulation, consistent with findings in tea plant [58] and bamboo [61]. Both conserved and tissue-specific ASEGs are involved in a range of physiological processes. These findings imply that ASE may orchestrate a trade-off between core metabolic maintenance and adaptive plasticity. This may confer evolutionary advantages by balancing growth-defence resource allocation in diploid pineapple. Heterozygous and homozygous polymorphic TE insertions were detected in the promoter regions of ASEGs across pineapple haplotypes. In this case, specific TE insertions (e.g. MITE/DTC in *AcSBT1.7* and LTR/Gypsy in *AcUFC*) in the upstream regulatory regions are associated with significantly reduced expression of the corresponding alleles. These insertions likely act as potent episodic epimutagens, disrupting transcription factor binding or chromatin accessibility via DNA hypermethylation, thereby enforcing allelic expression imbalance [62, 63]. Similar TE-mediated allelic suppression has been reported in carnation [64] and citrus [56]. Conversely, TE insertions may also enhance gene expression, as revealed in red-skinned apples, where a retrotransposon upregulated *MdMYB1* to drive anthocyanin accumulation [65].

NLRs play essential roles in plant immunity by detecting pathogen effectors and show extensive structural diversification among plant species [66, 67]. Our newly assembled T2T and fully phased pineapple genome enables high-resolution analysis of NLR polymorphisms between haplotypes (Section Haplotype-dynamic NLR repertoires in F180). NLR genes typically cluster near the telomeres, where they physically interact to optimize immune responses [68]. Proximal duplication likely drove the expansion of CNL genes specific to HB on Chr13. This expansion may be favoured by pathogen-driven selection, thereby increasing immune gene copy numbers in HB (HA: 99 vs HB: 113). We identified 78 allelic NLR pairs, with 27 and 42 NLR genes specifically found in HA and HB, respectively. This uneven interhaplotype gene distribution likely reflects an evolutionary trade-off between persistent pathogen pressure and the fitness costs of expanding NLR repertoires [69, 70]. HA- and HB-specific NLR genes (e.g. *HA005713* and *HB000517*) exhibited high expression levels (FPKM > 100) in certain tissues, suggesting localized immune functions. Their pathogen-response specificity warrants further investigation.

## Conclusion

Beyond pineapple, this study underscores the broader value of haplotype-resolved genomes for understanding heterozygous, clonally propagated crops. The high contiguity (N50 of 16.38–16.86 Mb), completeness (BUSCO >99%), and resolution of almost all 50 telomeres and centromeres position the F180 assembly as a high-quality reference, surpassing most existing pineapple genomes. A comparison of the two haplotypes displays the large differences inherent in the ‘Smooth Cayenne’ pineapple genome, wherein 1.5 million SNPs and 1953 large SVs, including 74 inversions, 750 translocations, and 1129 segmental duplications, were identified. Inversions were found to be a particularly important process, collectively reshaping 3%–4% of the genome. Among these, a 1.3-Mb paracentric inversion on chromosome 20 acts as a true recombination coldspot, whereas a 6-Mb pericentric inversion on chromosome 24 still supports gene flow, likely via short DCOs. This contrast demonstrates that chromosomal context, not size alone, dictates whether inversions hinder or permit allelic shuffling, a principle likely to hold in many perennial crops where undetected inversions can stall genetic gain. These insights extend to other perennial crops where large inversions may silently constrain genetic gain. The resource therefore enables fundamental studies of how heterozygosity, structural variation, and allele-specific expression interact in adaptation and domestication, while guiding both conventional and genome-editing strategies to broaden pineapple’s genetic base and mitigate vulnerability to biotic and abiotic stress.

## Materials and methods

### Sample collection, nucleic acid extraction and sequencing, and Hi-C library preparation and sequencing

For long-read DNA sequencing, meristematic tissue taken from the D leaf of a mature ‘Smooth Cayenne’ clone F180 pineapple plant was collected from a commercial farm, ‘Sandy Creek Pineapples’ located in the Glasshouse Mountains, QLD (−26.872209, 152.962088). The sample was immediately snap-frozen using liquid nitrogen, and then stored at −80°C. The Tissue Lyser II instrument under cryogenic conditions was used to homogenize the sample. The cetyltrimethylammonium bromide (CTAB) method, as described by Furtado [71], was used to extract whole genomic DNA. Long-read sequencing was completed at the Institute for Molecular Bioscience, University of Queensland, using two PacBio HiFi SMRT Cells on the Sequel II instrument. For the annotation, total RNA was extracted from five different tissues, including three leaves, one root, and a combined fruit and flower tissue (Fig. 3a), using the NucleoSpin RNA Plant Mini Kit from Macherey-Nagel. Total RNA was sequenced using the MGI T7 DNBSEQ platform. RNA-seq library construction followed standard protocols, beginning with RNA quality assessment using an Agilent 4200 Tape Station to ensure RNA integrity (RIN ≥ 6). rRNA was depleted, then the remaining RNA fragmented, followed by first- and second-strand cDNA synthesis, end repair, A-tailing, and adapter ligation using the MGIEasy RNA library prep kit. Following ligation, libraries were PCR amplified and size-selected (~300 bp). The final step involved circularization and rolling circle amplification to generate DNA nanoballs (DNBs), which were then loaded onto patterned flow cells for sequencing. Immature leaves attached to the shoot apex were collected from F180 for Hi-C library preparation and Illumina sequencing, which was carried out by DNA Zoo at the University of Western Australia. Library preparation and analysis were conducted following the *in situ* Hi-C protocol and data pipeline described by Rao *et al.* [72].

### Genome assembly

Prior to genome assembly, the *k*-mers (21-mers) of PacBio HiFi subreads were counted using Jellyfish (v2.2.10) [73], and then GenomeScope2 [74] was applied to estimate the genome sizes, heterozygosity, and repeat contents based on the 21-mers count histograms. We adopted a custom assembly workflow to assemble the haplotype-resolved genome (Fig. S3). First, based on HiFi and Hi-C reads, Hifiasm (v0.19.5-r587) [5] was applied to build a phased diploid assembly, generating primary contigs of 603.89 and 424.73 Mb, with N50 values of 6.83 and 6.90 Mb, respectively. Then, with the aid of Hi-C data, HapHiC (v1.0.5) [75] was used to *de novo* cluster, reassign, order, and orient the first (HA, 603.89 Mb) and second (HB, 424.73 Mb) haplotype contigs. Initially, the HA contigs were anchored into 25 groups with sizes ranging from 9.48 to 38.56 Mb, while the HB contigs were clustered into 25 groups with sizes ranging from 5.25 to 20.95 Mb. After constructing the final scaffolds, HapHiC automatically generated a shell script for visualization and curation in JuiceBox. Then, JuiceBox [76] was used for visualizing Hi-C interactions and performing manual modification to obtain 25 pseudo-chromosomes in each haplotype assembly (HA and HB). Telomere regions were identified using quarTeT (v1.2.1) [77] based on the telomeric seven-base repeat pattern (CCCTAAA/TTTAGGG at the 5′/3′ end) in the plant genome. In the manual modification process, contigs containing telomeres were manually examined to ensure their proper placement at the chromosome ends. Some unplaced contigs were also rescued according to the strong Hi-C interaction signals. Finally, the BAM file and agp file containing the contigs from both haplotype assemblies were regenerated and JuiceBox was used to visualize the combined Hi-C contact matrix. Some redundant or excess contigs incorrectly phased by Hifiasm were manually swapped between HA and HB according to Hi-C signals. In addition to the telomeres at the chromosomal level, we also identified seven small unanchored contigs (ranging from 21.5 to 96.6 kb) with telomeres at one end. The initial genome assembly was searched using BLASTN (2.15.0+) [78] against the chloroplast and mitochondrial genome sequences of *A. comosus* downloaded from the NCBI GenBank to remove the organelle-derived contigs. Next, genome self-alignment was performed using Minimap2 (v2.28) [79] to eliminate redundant sequences, yielding the final F180 genome assembly. Only three of the seven small unanchored contigs with telomeres remained in the final assembly. BLASTN was further used to determine whether the telomeres on these unanchored contigs match the telomere-lacking ends of the chromosomes.

### Assessment of genome assembly quality

Multiple approaches were involved to assess the quality of genome assembly and phasing. JuiceBox was used for visualizing the Hi-C interaction signals. QuarTeT [77] was used to perform telomere identification and centromere candidate prediction. The assembly contiguity was assessed using QUAST (v5.0.2) [80]. The completeness of the genome and gene model was assessed using the latest version of BUSCO (v5.8.0) [81] (Benchmarking Universal Single-Copy Orthologs) with the Miniprot [82] and MetaEuk [83] tool, based on 1614 highly conserved plant core genes from the ‘embryophyta_odb10’ dataset. The base-level accuracy and completeness of the genome assembly were evaluated with Merqury (v1.3) [84], using consensus quality value (QV) scores estimated from the *k*-mer spectrum of the PacBio HiFi read set with a 19-mer. The LAI, calculated using the LTR_retriever pipeline (v2.9.0), was also used to assess assembly quality. The PacBio HiFi reads, stLFR paired-end short reads, and RNA-seq reads were mapped to the genome assembly to assess assembly accuracy using Minimap2 [79], BWA (v0.7.17-r1188) [85], and HISAT2 (v2.2.1) [86], respectively. SAMtools (v1.9) [87] subcommands ‘depth’ and ‘coverage’ were used to obtain base depth and coverage metrics for each chromosome.

### Genome repeats and gene annotation

Before annotation, we manually curated the chromosome order and orientation for the two haplotypes based on the Hi-C interaction heatmap, ensuring chromosomes were arranged from largest to smallest, with the short arms in front and long arms at the rear. Genomic transposable and repeat elements (TEs) were *de novo* identified by RepeatModeler2 (v2.0.4) [88] with default parameters. The output file ‘genome-families.fa’ was subsequently used to mask the repeat regions in the genome with RepeatMasker (v4.1.5) [89] using the ‘-xsmall’ soft-masking option to enable prediction of PCGs containing repetitive sequences [90]. The quality-controlled and trimmed paired-end RNA-seq reads were aligned to the soft-masked genomes using HISAT2 (v2.2.1) [86]. PCGs were annotated using the BRAKER3 annotation pipeline [91], which combines GeneMark-ETP, AUGUSTUS, and the TSEBRA combiner, incorporating both transcriptome evidence and a large protein database, with statistical models that are iteratively trained and specifically tailored to the target genome. The RNA-seq transcripts were obtained from five pineapple tissue samples: root, leaf meristem, leaf mid-section, leaf tip, and a combined flower and fruit sample. The gene annotation completeness of predicted PCGs in HA and HB was evaluated using BUSCO (v5.8.0). Functional annotation of PCGs was performed through (i) BLASTP searches (E-value ≤1e−5) against NCBI nonredundant (Nr; *Viridiplantae* taxonomy) and Swiss-Prot protein databases; (ii) functional assignment using Gene Ontology (GO), Kyoto Encyclopedia of Genes and Genomes (KEGG), and InterProScan; and (iii) orthologous group classification by Clusters of Orthologous Groups (COGs) and EggNOG. Barrnap (v0.9) (https://github.com/tseemann/barrnap) was used to predict rRNAs, including 5S, 5.8S, 18S, and 28S rRNA genes, in the F180 genome. The tRNA genes were predicted by tRNAscan-SE (v2.0.12) [92] with default parameters.

### Identification of structural variations, allelic gene pairs, and allele-specific expression

Genome collinearity and structural variations, including presence/absence variants (PAVs; >50 bp insertions/deletions), between F180 subgenomes HA and HB were identified using Synteny and Rearrangement Identifier (SyRi v1.7.0) [93] and a custom Python script. SNPs were called using GATK (v4.2.6.1) [94] through (1) variant calling (SNPs and InDels) with HaplotypeCaller (--stand-call-conf 30); (2) filtering with VariantFiltration using the parameters: ‘DP < 1||DP > 400||QD < 2.0||FS > 60.0||MQ < 40.0||MQRankSum < −12.5||ReadPosRankSum < −8.0’; and (3) final selection of high-confidence SNPs with SelectVariants. The SnpEff [95] (v5.2) software was used to annotate the potential effects of variations within the pineapple ASEGs based on the gene annotations of the reference genome (HA) with default parameters. InDels were identified using Assemblytics (v1.2.1) [96] based on whole-genome alignments generated by NUCmer from MUMmer [97] (v4).

Whole-genome nucleotide collinearity was assessed using Minimap2 [79], NUCmer [97], and Mauve (v2.40) [98] under default parameters. Dot plots were generated from Minimap2 PAF files using a modified paf2dotplot script (https://github.com/zengxiaofei/paf2dotplot), and from NUCmer.nc.coords files using a custom Python script. Whole-genome dot plot alignments were performed to compare the two haplotypes and to assess F180 haplotypes against published assemblies of pineapple cultivars and related varieties, confirming the presence or absence of inversions 553 and 560 across lineages. Sequences used for the dot plots are detailed in Table S14. Synteny analysis at the PCG level was performed to assess the impact of structural rearrangements on gene organization. All annotated protein sequences from HA and HB were compared using all-against-all BLASTP searches (E-value ≤1e−5), and the resulting homologous pairs were fed into MCScanX [29] to identify collinear gene blocks. A synteny-based toolkit SynGap (v1.2.5) [99] *genepair* module was applied to profile integrative gene synteny between homologous chromosomes, using the HA and HB genome sequences and the GFF3 annotation files of the longest transcripts as input. The output file (‘*.final.genepair’), including the syntenic and the best two-way BLAST gene pairs, was considered as the allele table.

An RNA-seq analysis pipeline was employed to examine the differential expression of each allele pair in the allele table. In detail, for triplicate RNA-seq reads from five distinct tissues, roots, leaf meristems, leaf mid-sections, leaf tips, and flowers/fruits, we first used fastp (v0.23.4) to remove adapters and filter out low-quality bases/reads with parameters ‘-q 20 -l 75 -w 8’. The trimmed reads were then aligned to the combined F180 haplotype assembly using HISAT2 (v2.2.1). The output SAM (Sequence Alignment/Map) files were filtered to retain uniquely mapped reads (NH:i:1). The filtered SAM files were then converted to BAM (Binary Alignment/Map) format, sorted, and indexed by SAMtools, and subsequently fed into FeatureCounts (v1.6.2) for quantification of FPKM (fragments per kilobase of exon model per million mapped reads) values. The expression count matrix for all allele pairs was constructed, and differential expression was assessed using DESeq2 (v1.46.0) in R. The correlation analysis of the allelic expression was conducted using corrplot (v0.84) package in R software.

Allele-specific expression (ASE) was defined by an absolute log_2_FoldChange > 1 with *P* < .05. ASE patterns were classified into two categories: consistent ASE and direction-shifting ASE [57]. ASE patterns were classified as consistent if they exhibited stable allelic imbalance towards one allele across all five tissues (fixed log_2_FoldChange sign), or as direction-shifting if allelic imbalance direction showed intertissue reversal (sign flip). The log_2_FoldChange heatmaps were generated using the R packages ggplot2 (v3.5.1), tidyr (v1.3.1), and dplyr (v1.1.4). The upset plot was created using UpSetR package (v1.4.0). For the ASEG sets derived from different tissues, GO enrichment analysis was performed using the ‘GO enrichment’ function in TBtools (v1.113) [100]. The box plots of *Ka*, *Ks*, and *Ka*/*Ks* between ASEGs and non-ASEGs were visualized by ggplot2 and ggsignif (v0.6.4). Expression patterns of ASEGs were created using TBtools with the ‘Log_2_ Scale’ option. A range of genomic features, including chromosome length, gene density, repetitive elements, GC content, SVs, SNPs, Indels, and ASEGs were visualized on each pseudo-chromosome using Circos (v0.69.8) [101]. TEs were also independently identified in each haplotype assembly using EDTA [102] (v2.2.2) with the ‘-sensitive 1 -anno 1’ parameter settings. The TEanno.gff3 files were used to analyse the correlation with gene expression.

### Analysis of recombination modulation by haplotype-specific inversions

A total of 953 pineapple plants were generated for linkage analyses, including 512 seedlings spanning 17 biparental families plus 53 parents, and 374 seedlings from reciprocal crosses between ‘Smooth Cayenne’ × ‘MD-2’. Freeze-dried leaf bases (meristem) were sent to Diversity Arrays Pty Ltd for DArTseq™ genotyping. Genomic DNA was digested with PstI/MseI, ligated to barcoded adapters, and SNPs were called with the DArTsoft14 pipeline [103, 104]. A total of 32 141 marker sequences were aligned to the collapsed ‘Smooth Cayenne’ F153 v7 assembly and to both phased haplotypes of the F180 v4 T2T genome. Aligned genomic regions with an E-value <1e−7 were retained and the proportion of markers with >1 aligned region was calculated. After filtering for call rate (>0.75), minor allele frequency (>0.025), and unique chromosomal position, 11 879 high-quality SNPs were retained. Missing genotypes were imputed in TASSEL v5.2.44 with the LD-KNNi algorithm at 96% masking accuracy [105, 106], and these data were used to calculate interchromosomal linkage disequilibrium (LD) (*R*^2^ ≥ 0.90) and visualized as Circos plots with the GWLD package [107]. To track recombination derived from F180, a subset of this data comprising the parent cultivars F180 and ‘MD-2’ and 374 of their progeny was filtered to retain only markers that were heterozygous in F180 and homozygous in ‘MD-2’. This ensured that segregation patterns in the progeny reflected recombination events occurring exclusively in F180, while ‘MD-2’ contributed a constant allele background. Progeny SNP calls were recoded under the A/B system, where ‘A’ represented the first F180 allele together with the ‘MD-2’ allele, and ‘B’ represented the second F180 allele together with the ‘MD-2’ allele. The marker file was analysed in R using the QTL2 package with the ‘haploid’ model [108]. The Kosambi mapping function (scale factor 2^7^) was applied to convert physical distances into genetic distances, with recombination frequencies capped at 0.5, producing map lengths of up to ~130 cM for the largest chromosomes. The function locate_xo() in the Qtl2 package was used to identify crossover events. This function uses the genotype data and the genetic map to locate crossover positions represented in each individual. Genetic probabilities with an assumed error rate of 0.01 were used to account for genotyping errors. Additionally, the maxmarg() function was used to filter genotypes based on a minimum posterior probability (minprob = 0.8), ensuring that only high-confidence genotype calls were retained. SCOs were defined as intervals ≥0.25 cM. The individual seedling data were then collated into one dataset for F180 using a sliding window of 5 cM to avoid counting crossover events more than once during collation, and DCOs were identified when two consecutive SCOs were separated by ≤20 cM.

### Identification of putative pineapple NLR genes

The RGAugury [109] pipeline was used to predict NLR genes in each haplotype genome (HA and HB). The putative NBS domain-encoding genes were further classified into different subgroups based on their domain structures, including CN [coiled-coil (CC) and NB-ARC], CNL [CC, NB-ARC, and leucine-rich repeat (LRR)], NBS (NB-ARC), NL (NB-ARC and LRR), RNL (RPW8, NB-ARC and LRR), TN [Toll/interleukin-1 receptor (TIR) and NB-ARC], and TX (TIR and unknown domain). The distribution of NLR genes on chromosomes was drawn using RIdeogram (v0.2.2) [110] in R. NLR protein sequences were aligned using MUSCLE (v3.8.1) [111] with default settings and then subjected to IQ-TREE [112] (v2.0.3) for phylogenetic tree construction with 1000 bootstrap replicates. The ‘intersect’ function embedded in BEDTools (v2.30.0) [113] was used to assess the effects of inversion on NLR genes.

## Supplementary Material

Web_Material_uhag189

## Data Availability

All Pac-Bio CCS, Hi-C, and RNA-seq data have been deposited in the Genome Sequence Archive (GSA) in National Genomics Data Centre (NGDC, https://ngdc.cncb.ac.cn/) under project ID PRJCA041511. Whole-genome sequence data have been deposited in the Genome Warehouse (GWH) at NGDC under accession number GWHGEOA00000000.1 (Haplotype A) and GWHGEOB00000000.1 (haplotype B).
