# Supplementary material for: The heterozygous pineapple genome demonstrates the importance of haplotype-resolved plant genomes
Source: Hortic Res. 2026 May 8;13(10):uhag189. doi: 10.1093/hr/uhag189 (PMC13610578; doi:10.1093/hr/uhag189)
Supplement: Supplementary file 1 [file web_material_uhag189.zip › Supplementary Figure 1-clean version.docx]

**The heterozygous pineapple genome demonstrates the importance of haplotype-resolved plant genomes**

Jingping Fang^1,2^†, Patrick J. Mason^2,3^†, Garth Sanewski^4^, Matthew Webb^4^, Linwei Zhou^1^, Jinbin Wang^1^, Rhys G. R. Copeland^5^, Ying Zhuang^1^, Annapurna Chitikineni^5^, Vanika Garg^5^, Natalie Dillon^4^, Parwinder Kaur^6^, Rajeev K. Varshney^5^, Robert J. Henry^2,3,7^

**Supplementary Figures File 1**

**Supplementary Figure S1. The length distribution of PacBio reads for pineapple *F180*.** Histogram shows the distribution of read lengths (blue bars) with density on the y-axis and read length (bp) on the x-axis. A vertical dashed line marks 10 kb, highlighting the proportion of long reads above this threshold. Red tick marks along the x-axis indicate individual reads contributing to the distribution.

**Supplementary Figure S2. K-mer counting analysis based on PacBio subreads for pineapple *F180*.** GenomeScope profile showing the distribution of k-mer coverage (x-axis) versus frequency (y-axis). The observed k-mer spectrum (blue) is fitted with the full model (black line), separating unique sequences (yellow), sequencing errors (red), and k-mer peaks (dashed lines). Model estimates include a genome length of ~374 Mb, unique content (54.3%), heterozygosity (~1.6%), sequencing error rate (0.27%), and duplication level (1.36).

**Supplementary Figure S3. Workflow of the haplotype resolved genome assembly of pineapple *F180***

**Supplementary Figure S4. The prediction of telomeric repeat sequences in (a) HapA vs. (b) HapB.** Chromosomal ideograms show the positions of predicted telomeric repeat sequences (blue triangles) and assembly gaps (orange squares) in (a) haplotype A (HapA) and (b) haplotype B (HapB). Each chromosome is displayed to scale, ordered by length from left to right (Chr01–Chr25).

**Supplementary Figure S5. Prediction of centromeric repeat sequences and associated assembly gaps in (a) haplotype A (HapA) and (b) haplotype B (HapB).** Chromosomal ideograms display the positions of centromeres (constrictions) and assembly gaps (orange squares) across all chromosomes (Chr01–Chr25). This complements Figure S4, which highlights telomeric repeats and gaps, by showing the distribution of gaps in relation to centromeric regions.

**Supplementary Figure S6 Assembly of the *F180* collapsed genome, showing chimeric and ideal collapsed chromosomes.** (**a**) Examples of two chimeric chromosomes, with Hap A and Hap B nested within each other; (**b**) Examples of four ideal collapsed chromosomes, each with telomeres at both ends.

**Supplementary Figure S7. BUSCO completeness assessment of the haplotype-resolved *F180* pineapple genome (HA, HB, and merged assembly).** Analyses were performed using BUSCO v5.8.0 with the Miniprot tool against three lineage datasets: embryophyta_odb10, eukaryota_odb10, and viridiplantae_odb10. The results show high completeness across all assemblies, with the majority of genes classified as complete and single-copy.

**Supplementary Figure S8. BUSCO completeness assessment of the *F180* genome generated in this study compared with eight previously published pineapple genomes.** Analyses were performed using BUSCO v5.8.0 with the MetaEuk gene predictor against the embryophyta_odb10 dataset. Species abbreviations: GP (*‘Golden pineapple’, referred to as Ref*), LY (*‘Li Ye’*), BL (*‘Ba Li’*), YLL (*‘Yu Ling Long’*), GL1 (*‘Green Leaf 1’*), F153 (*Smooth Cayenne F153*), Yugafu (*Yugafu* 3C and 3H assemblies), and CB5 (*CB5*) [1-4].

**Supplementary Figure S9. Functional annotation of predicted protein-coding genes in the haplotype-resolved pineapple *F180* genome.** Venn diagrams show the overlap of annotated genes in haplotype (a) HA and (b) HB against five major public databases: NCBI non-redundant protein (NR), Gene Ontology (GO), Clusters of Orthologous Groups (COG), Kyoto Encyclopedia of Genes and Genomes (KEGG), and SwissProt. Numbers indicate the count of genes uniquely or jointly annotated by each database.

**Supplementary Figure S10. Synteny analysis of protein-coding genes between the haplotype-resolved *F180* genome and the published pineapple reference genome (GP) [2].** (a) Dot plot of collinear gene pairs between F180 haplotype A (HA) and GP; (b) Dot plot of collinear gene pairs between F180 haplotype B (HB) and GP; (c) Linear parallel comparison showing syntenic relationships among HA, HB, and GP. F180 chromosomes are arranged by size, with short arms oriented forward. The chromosome nomenclature and ordering of GP follow those of the F153 reference.

**Supplementary Figure S11. Distribution of inversion sizes across the 25 chromosomes for two haplotypes** **in the pineapple *F180* genome.** Each dot represents the length (bp) of an individual inversion for haplotype A (HA; blue) and haplotype B (HB; orange). The solid line connects the mean inversion length for each chromosome in HA and HB, respectively. The largest inversions occur on Chr24 (HA: 6.05 Mb; HB: 7.71 Mb), followed by large events on Chr11 and Chr20. Most other chromosomes contain comparatively smaller inversions.

**Supplementary Figure S12. Whole-genome synteny dot-plot at nucleotide level between F180 HA and HB.** The identity levels ranging from low to high are represented by colours from bule to red;

**Supplementary Figure S13. Hi-C interaction maps of chromosomes 11, 20, and 24 in HA and HB.**
Hi-C contact matrices for haplotypes A and B confirm high consistency of interaction signals along chromosomes 11 (a), 20 (b), and 24 (c). Each green box corresponds to one supercontig, and each royal blue box indicates a full chromosome. The diagonals highlight intra-chromosomal contacts, with interaction strength supporting the accuracy of chromosome-scale scaffolding in both haplotypes.

**Supplementary Figure S14. Synteny of protein-coding genes between the two F180 haplotypes.**
Genome-wide synteny analysis of all 25 chromosomes between haplotypes HA and HB was performed using MCScanX. Most chromosomes show high levels of gene collinearity, while large inverted regions are evident on chromosomes 11, 20, and 24, corresponding to the major structural variations between haplotypes. Blue and green rectangles represent genes transcribed in clockwise and counterclockwise orientations, respectively, with grey bars denoting intergenic regions.

**Supplementary Figure S15.** **Further confirmation of the third largest insertion (~1.3 Mb) on Chr20 using PacBio reads.** Two breakpoints (at 11,337,991 bp and 12,657,568 bp) of this inversion in HB-Chr20 were identified by mapping PacBio long reads against the F180 HB reference assembly and visualized in IGV. PacBio reads from HB successfully map across the breakpoints, while reads from HA do not.

**References**

1.	Yow, A.G., Bostan, H., Castanera, R. *et al.* Improved high-quality genome assembly and annotation of pineapple (*Ananas comosus*) cultivar MD2 revealed extensive haplotype diversity and diversified FRS/FRF gene family. *Genes*. 2021;**13**: 52

2.	Feng, J., Zhang, W., Chen, C. *et al.* The pineapple reference genome: Telomere-to-telomere assembly, manually curated annotation, and comparative analysis. *Journal of Integrative Plant Biology*. 2024;**66**: 2208-25

3.	Chen, L., VanBuren, R., Paris, M. *et al.* The bracteatus pineapple genome and domestication of clonally propagated crops. *Nature Genetics*. 2019;**51**: 1549-58

4.	Ming, R., VanBuren, R., Wai, C.M. *et al.* The pineapple genome and the evolution of CAM photosynthesis. *Nature Genetics*. 2015;**47**: 1435-42
