# Supplementary material for: The heterozygous pineapple genome demonstrates the importance of haplotype-resolved plant genomes
Source: Hortic Res. 2026 May 8;13(10):uhag189. doi: 10.1093/hr/uhag189 (PMC13610578; doi:10.1093/hr/uhag189)
Supplement: Supplementary file 1 [file web_material_uhag189.zip › Supplementary Figure 2-clean version.docx]

**Supplementary Figures File 2**

**Supplementary Figure S16. Dot plots of all major pineapple genomes against SC F180_HA.** Cultivar name codes are listed at the top with haplotype names underneath the black bar for each cultivar (* for collapsed genomes). Chromosome ID’s being compared are shown directly above each individual dot plot (Query genome vs Reference Genome). Rows are numbered 1 to 25 according to SC F180 reference chromosome numbering. X and Y axes are scaled to the size of the largest chromosome in the plot. Coordinates for the reference chromosome are plotted on the X axis and the query chromosome on the Y-axis. The best matching chromosome is plotted in blue whilst significant regions of other query chromosomes that also align are plotted in green. Variety abbreviations are detailed in **Table S12**.

**Supplementary Figure S17. Inversion conservation in haplotype-resolved homologues of SC F180 chromosomes 11, 20 and 24 as shown by dotplots.** Dot plots were generated using SC F180 haplotype A chromosomes as the reference and other haplotype resolved chromosomes as the query. The haplotype name and variety name are shown above each dot plot. Coordinates for the reference chromosome are plotted on the X axis and the query chromosome on the Y-axis. The inversions in chromosome 11, 20 and 24 of SC F180 are circled in red. Domesticated and wild varieties are indicated.

**Supplementary Figure S18. Inversion conservation in haplotype-resolved homologues of SC F180 chromosomes 11, 20 and 24 as shown by comparative synteny plots.** Variety and haplotype names are shown on the left and chromosome names (SC F180 numbering) on the right. Inversion and centromere locations (SC F180 haplotype A numbering) are shown above the SC F180 HA chromosomes. Homologous regions of each chromosome are colour coded. Inverted regions (relative to SC F180 haplotype A) are plotted below non-inverted regions for each chromosome. Inversions and deletions identified within BL haplotype 1 are marked with black and red lines respectively.

**Supplementary Figure S19. Venn diagram showing the distribution of genes between haplotypes HA and HB. A** total of 19,058 allele gene pairs are shared, while 1,196 genes are HA-specific and 1,183 genes are HB-specific.

**Supplementary Figure S20** ***Ka*/*Ks*-based selection patterns and functional enrichment of HA and HB allelic genes.** **a**. Distribution of Ka/Ks between allele gene pairs; **b-c**. GO enrichment analysis of potential genes under positive selection (Ka/Ks >1) in HA (b) and HB (c), respectively;

**Supplementary Figure S21.** **Consistent ASEG expression heatmap.** Genes located on chromosomes that exhibit a consistent allele-specific expression (ASE) pattern across five tissues. The color bar represents log_2_(FoldChange) values, where orange indicates significantly higher expression of allele A compared to allele B, and green denotes significantly higher expression of allele B compared to allele A.

**Supplementary Figure S22. Heatmap showing allele-specific expression genes (ASEGs) with inconsistent expression patterns (direction-shifting) across five tissues: root, leaf meristem, leaf mid-section, leaf tip, and flower/fruit.** The scale represents log₂ fold change (FC), with orange indicating higher expression bias toward one allele and green indicating bias toward the alternate allele.

**Supplementary Figure S23. Gene Ontology (GO) enrichment analysis of allele-specific expression genes (ASEGs) identified across five tissues.** The y-axis shows significantly enriched GO terms, grouped by molecular function, biological process, and cellular component categories. The x-axis represents the RichFactor (the ratio of ASEGs annotated to a given GO term relative to the total genes in that term). Dot size indicates the number of ASEGs associated with each GO term, while color corresponds to the statistical significance (−log₁₀ *P*-value).

**Supplementary Figure S24. Comparison of the differential expression of allele-specific expression genes (ASEGs) located within inversion regions versus collinear regions across five tissues.** Boxplots display the distribution of log₂ fold change values, with orange representing genes in inversion regions and blue representing collinear genes. Individual points correspond to ASEGs. Statistical significance between groups was assessed, with “ns” indicating no significant difference**.**

**Supplementary Figure S25.** **Distribution of NLR (Nucleotide-binding Leucine-rich Repeat) genes across haplotypes.** NLR genes were categorized into seven subgroups based on conserved domains: **CN** (CC and NB-ARC), **CNL** (CC, NB-ARC, and LRR), **NBS** (NB-ARC), **NL** (NB-ARC and LRR), **RNL** (RPW8, NB-ARC, and LRR), **TN** (TIR and NB-ARC), and **TX** (TIR and an unknown domain). Stacked bar plots show the number of NLRs per chromosome in **haplotype A (a)** and **haplotype B (b)**.

**Supplementary Figure S26. Distribution of predicted NLR genes across chromosomes in haplotypes HA and HB.** The bar plot shows the number of NLR genes on each chromosome, with HA represented in green and HB in orange. Differences in NLR counts between haplotypes are visible across several chromosomes, with notable enrichment on chromosomes 3, 4, and 13.

**Supplementary Figure S27. Chromosomal distribution of NLR genes in haplotypes HA (a) and HB (b).** NLR genes are shown as colored triangles, with different subgroups (CNL, RNL, NL, TX, TN, CN, NBS) indicated by color. The background heatmap represents gene density in 100 kb windows, with warmer colors (orange-red) showing regions of higher density and cooler colors (yellow-white) showing lower density.

**Supplementary Figure S28. Raincloud plots showing the distribution of log₂(FPKM+1) values for NLR genes in (a) HA and (b) HB across five tissues.** Each plot combines boxplots, density distributions, and individual data points. The grey dashed line connects the mean expression levels across tissues. Tissues: T01, root; T02, leaf meristem; T03, leaf mid-section; T04, leaf tip; T05, flower/fruit.

**Supplementary Figure S29. Boxplots of expression levels (log₂(FPKM+1)) for HA- and HB-specific NLR genes across five tissues.** Blue horizontal lines within boxes indicate median values, while white stars mark mean values. Tissues: T01, root; T02, leaf meristem; T03, leaf mid-section; T04, leaf tip; T05, flower/fruit.

**Supplementary Figure S30. Clustered heatmap of expression levels (log₂(FPKM+1)) for haplotype-specific NLR genes across five tissues.** (a) HA-specific NLR genes; (b) HB-specific NLR genes. Expression values are shown on a red gradient scale, with darker shades indicating higher expression. Tissues: T01, root; T02, leaf meristem; T03, leaf mid-section; T04, leaf tip; T05, flower/fruit.
