# Supplementary material for: The heterozygous pineapple genome demonstrates the importance of haplotype-resolved plant genomes
Source: Hortic Res. 2026 May 8;13(10):uhag189. doi: 10.1093/hr/uhag189 (PMC13610578; doi:10.1093/hr/uhag189)
Supplement: Supplementary file 1 [file web_material_uhag189.zip › Supplementary Tables 1-clean version.docx]

**The heterozygous pineapple genome demonstrates the importance of haplotype-resolved plant genomes**

Jingping Fang^1,2^†, Patrick J. Mason^2,3^†, Garth Sanewski^4^, Matthew Webb^4^, Linwei Zhou^1^, Jinbin Wang^1^, Rhys G. R. Copeland^5^, Ying Zhuang^1^, Annapurna Chitikineni^5^, Vanika Garg^5^, Natalie Dillon^4^, Parwinder Kaur^6^, Rajeev K. Varshney^5^, Robert J. Henry^2,3,7^

**Supplementary Tables File 1**

**Supplementary Table S1.** Summary of PacBio subreads and Hi-C data for pineapple *F180* genome.

| **Pineapple *F180*** | | |
| --- | --- | --- |
| **PacBio subread data** | Sequencing Tissue | Leaf |
|  | Sequencing Platform | Sequel II |
|  | SMRT Cells | 2 |
|  | Subread Ng50 (bp) | 26,979 |
|  | Largest (bp) | 48,324 |
|  | Average (bp) | 16,213 |
|  | Median (bp) | 15,586 |
|  | No. of Seq | 2,857,630 |
|  | Total size (Gb) | 46.33 |
|  | Coverage | 46.33 x |
| **HiC data** | Total size (Gb) | 39,859,198,200 |
|  | Coverage | 40 x |
| **StFLR** | Total size (Gb) | 89,552,289,600 |
|  | Coverage | 89.55 x |

**Supplementary Table S2. Correspondence of chromosome designations among the F180 haplotypes (HA and HB) and the previously published pineapple reference genomes F153** [1] **and GP** [2]**.** Chromosomes in F180 are ordered by descending physical length and oriented with the short arm first. This table provides the correspondence to the identifiers used in F153 and GP for cross-study comparison.

| **F180HA** | **Length (bp)** | **F180HB** | **F153** | **GP Ref** |
| --- | --- | --- | --- | --- |
| AcHAChr01 | 21,073,156 | AcHBChr01 | contig8 | contig08 |
| AcHAChr02 | 20,711,763 | AcHBChr02 | contig3 | contig03 |
| AcHAChr03 | 20,293,530 | AcHBChr03 | contig4 | contig04 |
| AcHAChr04 | 20,289,438 | AcHBChr04 | contig5 | contig05 |
| AcHAChr05 | 18,422,693 | AcHBChr05 | contig14 | contig14 |
| AcHAChr06 | 18,305,085 | AcHBChr06 | contig2 | contig02 |
| AcHAChr07 | 18,302,402 | AcHBChr07 | contig16 | contig16 |
| AcHAChr08 | 18,225,227 | AcHBChr08 | contig11 | contig11 |
| AcHAChr09 | 17,813,626 | AcHBChr09 | contig6 | contig06 |
| AcHAChr10 | 17,150,284 | AcHBChr10 | contig7 | contig07 |
| AcHAChr11 | 16,522,577 | AcHBChr11 | contig10 | contig10 |
| AcHAChr12 | 16,375,972 | AcHBChr12 | contig9 | contig09 |
| AcHAChr13 | 16,273,412 | AcHBChr13 | contig12 | contig12 |
| AcHAChr14 | 15,897,815 | AcHBChr14 | contig25 | contig25 |
| AcHAChr15 | 15,844,553 | AcHBChr15 | contig15 | contig15 |
| AcHAChr16 | 15,772,444 | AcHBChr16 | contig13 | contig13 |
| AcHAChr17 | 15,397,395 | AcHBChr17 | contig18 | contig18 |
| AcHAChr18 | 15,078,735 | AcHBChr18 | contig19 | contig19 |
| AcHAChr19 | 14,756,916 | AcHBChr19 | contig21 | contig21 |
| AcHAChr20 | 14,356,210 | AcHBChr20 | contig23 | contig23 |
| AcHAChr21 | 14,326,524 | AcHBChr21 | contig1 | contig01 |
| AcHAChr22 | 13,960,097 | AcHBChr22 | contig20 | contig20 |
| AcHAChr23 | 13,487,456 | AcHBChr23 | contig22 | contig22 |
| AcHAChr24 | 12,884,372 | AcHBChr24 | contig17 | contig17 |
| AcHAChr25 | 12,486,059 | AcHBChr25 | contig24 | contig24 |

**Supplementary Table S3.** Summary of chromosome lengths of the pineapple genome *F180.*

| **Hap1** | | | **Hap2** | | |
| --- | --- | --- | --- | --- | --- |
| **Chrom No.** | **Length (bp)** | **Telomeres** | **Chrom No.** | **Length (bp)** | **Telomeres** |
| AcHAChr01 | 21,073,156 | both | AcHBChr01 | 20,479,646 | left |
| AcHAChr02 | 20,711,763 | both | AcHBChr02 | 19,656,818 | both |
| AcHAChr03 | 20,293,530 | right | AcHBChr03 | 20,194,577 | both |
| AcHAChr04 | 20,289,438 | both | AcHBChr04 | 19,975,428 | both |
| AcHAChr05 | 18,422,693 | both | AcHBChr05 | 17,645,622 | both |
| AcHAChr06 | 18,305,085 | both | AcHBChr06 | 16,828,841 | both |
| AcHAChr07 | 18,302,402 | both | AcHBChr07 | 17,578,487 | both |
| AcHAChr08 | 18,225,227 | both | AcHBChr08 | 18,155,025 | both |
| AcHAChr09 | 17,813,626 | both | AcHBChr09 | 19,398,914 | both |
| AcHAChr10 | 17,150,284 | both | AcHBChr10 | 17,056,655 | both |
| AcHAChr11 | 16,522,577 | both | AcHBChr11 | 15,889,516 | both |
| AcHAChr12 | 16,375,972 | both | AcHBChr12 | 17,830,936 | both |
| AcHAChr13 | 16,273,412 | both | AcHBChr13 | 16,857,339 | both |
| AcHAChr14 | 15,897,815 | both | AcHBChr14 | 17,282,160 | right |
| AcHAChr15 | 15,844,553 | both | AcHBChr15 | 15,314,283 | both |
| AcHAChr16 | 15,772,444 | right | AcHBChr16 | 15,450,301 | both |
| AcHAChr17 | 15,397,395 | both | AcHBChr17 | 14,995,468 | both |
| AcHAChr18 | 15,078,735 | both | AcHBChr18 | 15,086,980 | both |
| AcHAChr19 | 14,756,916 | both | AcHBChr19 | 15,094,165 | both |
| AcHAChr20 | 14,356,210 | both | AcHBChr20 | 13,759,285 | both |
| AcHAChr21 | 14,326,524 | both | AcHBChr21 | 14,528,989 | both |
| AcHAChr22 | 13,960,097 | both | AcHBChr22 | 13,868,059 | both |
| AcHAChr23 | 13,487,456 | both | AcHBChr23 | 13,137,413 | both |
| AcHAChr24 | 12,884,372 | both | AcHBChr24 | 14,644,352 | both |
| AcHAChr25 | 12,486,059 | right | AcHBChr25 | 12,091,803 | right |
| N50 of 25 chromosomes | 16,522,577 |  | N50 of 25 chromosomes | 16,857,339 |  |
| GC% of 25 chromosomes | 39.15 |  | GC% of 25 chromosomes | 39.06 |  |
| **SubTotal Size** | **414.01 Mb** |  | **SubTotal Size** | **412.80 Mb** |  |
| No. of unanchored contigs | 278 |  | No. of unanchored contigs | 74 |  |
| Unanchored | 19.26 Mb |  | Unanchored | 12.4 Mb |  |
| No. of all contigs | 303 |  | No. of all contigs | 99 |  |
| N50 of all contigs | 16,375,972 |  | N50 of all contigs | 16,857,339 |  |
| GC% of all contigs | 40.09 |  | GC% of all contigs | 39.49 |  |
| **Total Size** | **433.27 Mb** |  | **Total Size** | **425.20 Mb** |  |

**Supplementary Table S4.** Prediction of the best candidates for centromeric repeat sequences and sizes in HapA and HapB.

| **Chrom.** | **Chrom. Length** | **Start Position of Centromere** | **End Position of Centromere** | **Size of Centromere** | **TR Length in Centromere** | **TR Coverage** | **Size of Short Arm** | **Size of Long Arm** |
| --- | --- | --- | --- | --- | --- | --- | --- | --- |
| AcHAChr01 | 21,073,156 | 9,757,324 | 11,176,671 | 1,419,348 | 372,300 | 26.23% | 9,757,324 | 9,896,486 |
| AcHAChr02 | 20,711,763 | 6,785,438 | 7,236,140 | 450,703 | 126,513 | 28.07% | 6,785,438 | 13,475,624 |
| AcHAChr03 | 20,293,530 | 6,950,180 | 8,705,304 | 1,755,125 | 946,704 | 53.94% | 6,950,180 | 11,588,227 |
| AcHAChr04 | 20,289,438 | 8,262,207 | 9,957,158 | 1,694,952 | 1,074,468 | 63.39% | 8,262,207 | 10,332,281 |
| AcHAChr05 | 18,422,693 | 5,243,941 | 5,523,737 | 279,797 | 181,479 | 64.86% | 5,243,941 | 12,898,957 |
| AcHAChr06 | 18,305,085 | 7,111,835 | 9,615,650 | 2,503,816 | 1,927,806 | 76.99% | 7,111,835 | 8,689,436 |
| AcHAChr07 | 18,302,402 | 6,041,841 | 9,041,667 | 2,999,827 | 1,842,143 | 61.41% | 6,041,841 | 9,260,736 |
| AcHAChr08 | 18,225,227 | 6,512,937 | 8,697,320 | 2,184,384 | 1,702,860 | 77.96% | 6,512,937 | 9,527,908 |
| AcHAChr09 | 17,813,626 | 6,378,880 | 7,951,008 | 1,572,129 | 613,825 | 39.04% | 6,378,880 | 9,862,619 |
| AcHAChr10 | 17,150,284 | 6,827,284 | 8,054,631 | 1,227,348 | 368,563 | 30.03% | 6,827,284 | 9,095,654 |
| AcHAChr11 | 16,522,577 | 6,709,999 | 7,396,910 | 686,912 | 522,762 | 76.10% | 6,709,999 | 9,125,668 |
| AcHAChr12 | 16,375,972 | 7,265,204 | 8,539,629 | 1,274,426 | 988,407 | 77.56% | 7,265,204 | 7,836,344 |
| AcHAChr13 | 16,273,412 | 5,516,251 | 6,687,590 | 1,171,340 | 623,754 | 53.25% | 5,516,251 | 9,585,823 |
| AcHAChr14 | 15,897,815 | 5,620,606 | 6,554,729 | 934,124 | 565,895 | 60.58% | 5,620,606 | 9,343,087 |
| AcHAChr15 | 15,844,553 | 4,503,146 | 7,308,791 | 2,805,646 | 2,379,199 | 84.80% | 4,503,146 | 8,535,763 |
| AcHAChr16 | 15,772,444 | 5,753,439 | 6,439,553 | 686,115 | 367,882 | 53.62% | 5,753,439 | 9,332,892 |
| AcHAChr17 | 15,397,395 | 4,533,564 | 6,868,888 | 2,335,325 | 1,760,172 | 75.37% | 4,533,564 | 8,528,508 |
| AcHAChr18 | 15,078,735 | 4,178,498 | 6,250,491 | 2,071,994 | 1,084,919 | 52.36% | 4,178,498 | 8,828,245 |
| AcHAChr19 | 14,756,916 | 4,330,745 | 6,368,839 | 2,038,095 | 856,471 | 42.02% | 4,330,745 | 8,388,078 |
| AcHAChr20 | 14,356,210 | 6,078,512 | 6,695,403 | 616,892 | 284,951 | 46.19% | 6,078,512 | 7,660,808 |
| AcHAChr21 | 14,326,524 | 4,789,430 | 5,523,847 | 734,418 | 563,096 | 76.67% | 4,789,430 | 8,802,678 |
| AcHAChr22 | 13,960,097 | 5,379,361 | 6,625,388 | 1,246,028 | 844,306 | 67.76% | 5,379,361 | 7,334,710 |
| AcHAChr23 | 13,487,456 | 5,808,225 | 7,610,405 | 1,802,181 | 1,367,413 | 75.88% | 5,808,225 | 5,877,052 |
| AcHAChr24 | 12,884,372 | **5,054,137** | **6,125,311** | 1,071,175 | 449,130 | 41.93% | 5,054,137 | 6,759,062 |
| AcHAChr25 | 12,486,059 | 2,883,737 | 3,035,430 | 151,694 | 89,503 | 59.00% | 2,883,737 | 9,450,630 |
| AcHBChr01 | 20,479,646 | 9,358,960 | 9,574,778 | 215,819 | 96,761 | 44.83% | 9,358,960 | 10,904,869 |
| AcHBChr02 | 19,656,818 | 6,727,202 | 7,249,118 | 521,917 | 190,524 | 36.50% | 6,727,202 | 12,407,701 |
| AcHBChr03 | 20,194,577 | 7,541,201 | 9,285,790 | 1,744,590 | 841,338 | 48.23% | 7,541,201 | 10,908,788 |
| AcHBChr04 | 19,975,428 | 8,335,210 | 10,028,066 | 1,692,857 | 630,239 | 37.23% | 8,335,210 | 9,947,363 |
| AcHBChr05 | 17,645,622 | 6,916,411 | 8,207,600 | 1,291,190 | 924,772 | 71.62% | 6,916,411 | 9,438,023 |
| AcHBChr06 | 16,828,841 | 7,016,260 | 8,400,406 | 1,384,147 | 949,522 | 68.60% | 7,016,260 | 8,428,436 |
| AcHBChr07 | 17,578,487 | 5,355,166 | 7,746,302 | 2,391,137 | 1,288,251 | 53.88% | 5,355,166 | 9,832,186 |
| AcHBChr08 | 18,155,025 | 6,493,759 | 8,706,017 | 2,212,259 | 1,700,244 | 76.86% | 6,493,759 | 9,449,009 |
| AcHBChr09 | 19,398,914 | 6,644,071 | 9,043,785 | 2,399,715 | 1,992,002 | 83.01% | 6,644,071 | 10,355,130 |
| AcHBChr10 | 17,056,655 | 6,807,118 | 8,444,822 | 1,637,705 | 450,553 | 27.51% | 6,807,118 | 8,611,834 |
| AcHBChr11 | 15,889,516 | 7,612,383 | 9,435,090 | 1,822,708 | 783,741 | 43.00% | 7,612,383 | 6,454,427 |
| AcHBChr12 | 17,830,936 | 7,156,159 | 8,255,734 | 1,099,576 | 884,455 | 80.44% | 7,156,159 | 9,575,203 |
| AcHBChr13 | 16,857,339 | 5,458,356 | 6,760,845 | 1,302,490 | 855,218 | 65.66% | 5,458,356 | 10,096,495 |
| AcHBChr14 | 17,282,160 | 4,986,505 | 7,719,946 | 2,733,442 | 1,997,057 | 73.06% | 4,986,505 | 9,562,215 |
| AcHBChr15 | 15,314,283 | 4,219,833 | 6,807,842 | 2,588,010 | 1,829,476 | 70.69% | 4,219,833 | 8,506,442 |
| AcHBChr16 | 15,450,301 | 5,290,845 | 6,185,960 | 895,116 | 484,709 | 54.15% | 5,290,845 | 9,264,342 |
| AcHBChr17 | 14,995,468 | 4,551,724 | 5,734,617 | 1,182,894 | 799,694 | 67.60% | 4,551,724 | 9,260,852 |
| AcHBChr18 | 15,086,980 | 3,357,511 | 6,053,091 | 2,695,581 | 2,169,986 | 80.50% | 3,357,511 | 9,033,890 |
| AcHBChr19 | 15,094,165 | 4,996,461 | 7,447,063 | 2,450,603 | 1,807,698 | 73.77% | 4,996,461 | 7,647,103 |
| AcHBChr20 | 13,759,285 | 5,956,931 | 7,724,258 | 1,767,328 | 1,230,677 | 69.63% | 5,956,931 | 6,035,028 |
| AcHBChr21 | 14,528,989 | 5,046,399 | 6,083,430 | 1,037,032 | 699,755 | 67.48% | 5,046,399 | 8,445,560 |
| AcHBChr22 | 13,868,059 | 5,324,006 | 6,871,127 | 1,547,122 | 1,047,770 | 67.72% | 5,324,006 | 6,996,933 |
| AcHBChr23 | 13,137,413 | 5,679,005 | 7,330,198 | 1,651,194 | 1,377,130 | 83.40% | 5,679,005 | 5,807,216 |
| AcHBChr24 | 14,644,352 | **918,499** | **2,893,773** | 1,975,275 | 1,024,779 | 51.88% | 918,499 | 11,750,580 |
| AcHBChr25 | 12,091,803 | 2,829,871 | 2,975,084 | 145,214 | 83,135 | 57.25% | 2,829,871 | 9,116,720 |

**Supplementary Table S5.** Detailed genome assembly, mapping, and annotation statistics for *Ananas comosus* var. Smooth Cayenne *F180.*

| **Category** | **HA** | **HB** | **Whole genome** |
| --- | --- | --- | --- |
| **Assembly** |  |  |  |
| Cumulative size (bp) | 414,007,741 | 412,801,062 | 826,808,803 |
| N50 (bp) | 16,522,577 | 16,857,339 | 16,828,841 |
| GC content (%) | 39.15 | 39.06 | 39.10 |
| Hi-C scaffolds | 25 | 25 | 50 |
| Complete BUSCO before adjustment (%) | 99.20 | 98.30 | 99.40 |
| Complete BUSCO after adjustment (%) | 99.20 | 99.40 | 99.50 |
| Complete and single-copy BUSCOs (S, %) | 97.30 | 97.40 | 97.50 |
| Complete and duplicated BUSCOs (D, %) | 1.90 | 1.90 | 1.90 |
| Fragmented BUSCOs (F, %) | 0.40 | 0.40 | 0.40 |
| Missing BUSCOs (M, %) | 0.40 | 0.30 | 0.20 |
| LAI | 24.82 | 23.23 | – |
| QV (p-value) | 65.21 (3.01×10⁻⁷) | 64.94 (3.21×10⁻⁷) | 65.07 (3.11×10⁻⁷) |
| Gaps | 65 | 77 | 142 |
| Telomeres detected | 47/50 | 47/50 | 94/100 |
| Centromeres detected | 25/25 | 25/25 | 50/50 |
| **Mapping rates** |  |  |  |
| Coverage of HiFi reads (%) | 99.96 | 99.96 | – |
| Mean depth of HiFi reads | 46.39 | 46.09 | – |
| Mapping rate of HiFi reads (%) | 99.62 | 99.62 | 99.62 |
| Coverage of stLFR paired-end reads (%) | 99.93 | 99.92 | – |
| Mean depth of stLFR paired-end reads | 78.28 | 78.24 | – |
| Mapping rate of stLFR paired-end reads (%) | 97.86 | 97.86 | 97.86 |
| **Transcriptome alignment rates** |  |  |  |
| Root (T01, %) | 83.11 | 83.24 | – |
| Leaf meristem (T02, %) | 92.95 | 92.98 | – |
| Mid-section (T03, %) | 93.12 | 92.99 | – |
| Leaf tip (T04, %) | 93.45 | 93.26 | – |
| Flower and fruit (T05, %) | 95.08 | 95.29 | – |
| **Annotation** |  |  |  |
| Number of genes | 20,280 | 20,248 | 40,528 |
| Number of mRNAs | 24,280 | 24,283 | – |
| Number of CDS | 24,280 | 24,283 | – |
| Number of exons | 146,189 | 146,452 | – |
| Number of introns | 121,909 | 122,169 | – |
| Single-exon genes | 3,957 | 3,993 | – |
| Total gene length (bp) | 93,160,210 | 93,117,281 | – |
| Total exon length (bp) | 32,562,721 | 32,774,115 | – |
| Total intron length (bp) | 86,586,822 | 87,071,487 | – |
| Mean gene length (bp) | 4,593 | 4,598 | – |
| Mean CDS length (bp) | 1,341 | 1,349 | – |
| Mean exon length (bp) | 222 | 223 | – |
| Mean intron length (bp) | 710 | 712 | – |
| Average exons per gene | 6.0 | 6.0 | – |
| Complete BUSCO for proteins (%) (embryophyta_odb10, n=1,614) | 99.10 | 99.00 | – |
| Complete BUSCO for proteins (%) (viridiplantae_odb10, n=425) | 99.80 | 99.60 | – |
| Complete BUSCO for proteins (%) (eukaryota_odb10, n=255) | 99.70 | 99.60 | – |
| **Functional annotation (mRNAs)** |  |  |  |
| NR annotated (%) | 23,755 (97.84) | 23,762 (97.85) | – |
| GO annotated (%) | 11,561 (47.62) | 11,485 (47.30) | – |
| eggNOG annotated (%) | 15,208 (62.64) | 15,217 (62.67) | – |
| KEGG annotated (%) | 11,932 (49.14) | 11,938 (49.16) | – |
| SWISSPORT annotated (%) | 18,330 (75.49) | 18,354 (75.58) | – |
| Annotated in all databases (%) | 6,414 (26.42) | 6,361 (26.20) | – |
| Annotated in ≥1 database (%) | 23,760 (97.86) | 23,764 (97.86) | – |
|  |  |  |  |

**Supplementary Table S6.** BUSCO assessments in pineapple genome *F180* in this study and eight published pineapple genomes with the gene predictor MetaEuk and Miniprot.

| **Miniprot** | **variety** | **Complete BUSCO(C)** | **Complete and single-copy BUSCOs(S)** | **Complete and duplicated BUSCOs(D)** | **Fragmented BUSCOs(F)** | **Missing BUSCOs(M)** | **lineage dataset** |
| --- | --- | --- | --- | --- | --- | --- | --- |
|  | F180 | 99.4 | 1.0 | 98.5 | 0.6 | 0.0 | embryophyta (n=1614) |
|  | HA | 99.3 | 97.3 | 1.9 | 0.7 | 0.0 |  |
|  | HB | 99.1 | 97.0 | 2.1 | 0.8 | 0.1 |  |
|  | F180_chr | 99.4 | 0.9 | 98.5 | 0.6 | 0.0 |  |
|  | HA_chr | 99.3 | 97.6 | 1.7 | 0.7 | 0.0 |  |
|  | HB_chr | 99.1 | 97.3 | 1.8 | 0.9 | 0.1 |  |
|  | F180 | 100.0 | 2.0 | 98.0 | 0.0 | 0.0 | eukaryota (n=255) |
|  | HA | 99.6 | 91.0 | 8.6 | 0.4 | 0.0 |  |
|  | HB | 99.6 | 89.4 | 10.2 | 0.4 | 0.0 |  |
|  | F180_chr | 100.0 | 2.4 | 97.6 | 0.0 | 0.0 |  |
|  | HA_chr | 99.6 | 91.4 | 8.2 | 0.4 | 0.0 |  |
|  | HB_chr | 99.6 | 91.0 | 8.6 | 0.4 | 0.0 |  |
|  | F180 | 99.8 | 0.2 | 99.5 | 0.2 | 0.0 | viridiplantae (n=425) |
|  | HA | 99.8 | 98.6 | 1.2 | 0.2 | 0.0 |  |
|  | HB | 99.8 | 98.1 | 1.6 | 0.2 | 0.0 |  |
|  | F180_chr | 99.8 | 0.2 | 99.5 | 0.2 | 0.0 |  |
|  | HA_chr | 99.8 | 98.6 | 1.2 | 0.2 | 0.0 |  |
|  | HB_chr | 99.8 | 98.8 | 0.9 | 0.2 | 0.0 |  |
| **Metaeuk** | **variety** | **Complete BUSCO(C)** | **Complete and single-copy BUSCOs(S)** | **Complete and duplicated BUSCOs(D)** | **Fragmented BUSCOs(F)** | **Missing BUSCOs(M)** | **lineage dataset** |
|  | F180 | 99.5 | 1.8 | 97.7 | 0.2 | 0.3 | embryophyta (n=1614) |
|  | HA | 99.2 | 96.9 | 2.3 | 0.4 | 0.4 |  |
|  | HB | 99.4 | 97.0 | 2.4 | 0.3 | 0.3 |  |
|  | F180_chr | 99.4 | 1.7 | 97.7 | 0.2 | 0.3 |  |
|  | HA_chr | 99.2 | 97.1 | 2.0 | 0.4 | 0.4 |  |
|  | HB_chr | 99.3 | 97.2 | 2.1 | 0.4 | 0.3 |  |
|  | F180 | 98.8 | 1.6 | 97.3 | 0.8 | 0.4 | eukaryota (n=255) |
|  | HA | 98.8 | 90.2 | 8.6 | 0.8 | 0.4 |  |
|  | HB | 98.4 | 88.6 | 9.8 | 1.2 | 0.4 |  |
|  | F180_chr | 98.8 | 1.6 | 97.3 | 0.8 | 0.4 |  |
|  | HA_chr | 98.8 | 90.6 | 8.2 | 0.8 | 0.4 |  |
|  | HB_chr | 98.4 | 90.2 | 8.2 | 1.2 | 0.4 |  |
|  | F180 | 99.3 | 1.4 | 97.9 | 0.5 | 0.2 | viridiplantae (n=425) |
|  | HA | 99.1 | 97.9 | 1.2 | 0.7 | 0.2 |  |
|  | HB | 99.3 | 97.6 | 1.6 | 0.5 | 0.2 |  |
|  | F180_chr | 99.1 | 1.2 | 97.9 | 0.7 | 0.2 |  |
|  | HA_chr | 99.1 | 97.9 | 1.2 | 0.7 | 0.2 |  |
|  | HB_chr | 99.1 | 97.9 | 1.2 | 0.7 | 0.2 |  |
| **Miniprot** | **variety** | **Complete BUSCO(C)** | **Complete and single-copy BUSCOs(S)** | **Complete and duplicated BUSCOs(D)** | **Fragmented BUSCOs(F)** | **Missing BUSCOs(M)** | **lineage dataset** |
|  | CB5 | 99.3 | 15.9 | 83.3 | 0.7 | 0.1 | embryophyta (n=1614) |
|  | F153 | 98.5 | 94.0 | 4.5 | 1.4 | 0.1 |  |
|  | GP | 99.2 | 97.7 | 1.5 | 0.8 | 0.0 |  |
|  | Yugafu.3C | 96.3 | 79.2 | 17.1 | 1.2 | 2.5 |  |
|  | Yugafu.3H | 92.3 | 80.2 | 12.1 | 1.4 | 6.3 |  |
|  | GL1 | 98.6 | 92.4 | 6.3 | 1.1 | 0.3 |  |
|  | BL_hap1 | 98.3 | 96.2 | 2.1 | 0.9 | 0.9 |  |
|  | BL_hap2 | 98.8 | 95.8 | 3.0 | 1.1 | 0.1 |  |
|  | LY_hap1 | 99.0 | 97.3 | 1.7 | 0.9 | 0.1 |  |
|  | LY_hap2 | 99.1 | 97.5 | 1.6 | 0.9 | 0.1 |  |
|  | YLL_hap1 | 98.8 | 95.4 | 3.4 | 0.9 | 0.4 |  |
|  | YLL_hap2 | 97.6 | 95.4 | 2.2 | 0.9 | 1.5 |  |
|  | CB5 | 100.0 | 11.8 | 88.2 | 0.0 | 0.0 | eukaryota (n=255) |
|  | F153 | 98.8 | 86.7 | 12.2 | 0.8 | 0.4 |  |
|  | GP | 100.0 | 91.8 | 8.2 | 0.0 | 0.0 |  |
|  | Yugafu.3C | 96.9 | 73.3 | 23.5 | 0.4 | 2.7 |  |
|  | Yugafu.3H | 90.2 | 72.5 | 17.6 | 1.6 | 8.2 |  |
|  | GL1 | 99.2 | 87.5 | 11.8 | 0.8 | 0.0 |  |
|  | BL_hap1 | 98.4 | 90.2 | 8.2 | 0.8 | 0.8 |  |
|  | BL_hap2 | 100.0 | 90.2 | 9.8 | 0.0 | 0.0 |  |
|  | LY_hap1 | 99.6 | 90.6 | 9.0 | 0.4 | 0.0 |  |
|  | LY_hap2 | 99.6 | 91.0 | 8.6 | 0.4 | 0.0 |  |
|  | YLL_hap1 | 99.2 | 85.9 | 13.3 | 0.4 | 0.4 |  |
|  | YLL_hap2 | 98.0 | 88.2 | 9.8 | 0.0 | 2.0 |  |
|  | CB5 | 99.8 | 13.6 | 86.1 | 0.2 | 0.0 | viridiplantae (n=425) |
|  | F153 | 99.3 | 94.1 | 5.2 | 0.7 | 0.0 |  |
|  | GP | 99.3 | 98.4 | 0.9 | 0.7 | 0.0 |  |
|  | Yugafu.3C | 96.5 | 77.6 | 18.8 | 1.2 | 2.4 |  |
|  | Yugafu.3H | 91.1 | 80.0 | 11.1 | 1.6 | 7.3 |  |
|  | GL1 | 99.1 | 93.2 | 5.9 | 0.9 | 0.0 |  |
|  | BL_hap1 | 98.6 | 96.7 | 1.9 | 0.2 | 1.2 |  |
|  | BL_hap2 | 99.1 | 96.7 | 2.4 | 0.7 | 0.2 |  |
|  | LY_hap1 | 99.8 | 98.8 | 0.9 | 0.2 | 0.0 |  |
|  | LY_hap2 | 99.1 | 97.4 | 1.6 | 0.9 | 0.0 |  |
|  | YLL_hap1 | 99.1 | 95.5 | 3.5 | 0.7 | 0.2 |  |
|  | YLL_hap2 | 97.6 | 95.5 | 2.1 | 1.2 | 1.2 |  |
| **Metaeuk** | **variety** | **Complete BUSCO(C)** | **Complete and single-copy BUSCOs(S)** | **Complete and duplicated BUSCOs(D)** | **Fragmented BUSCOs(F)** | **Missing BUSCOs(M)** | **lineage dataset** |
|  | CB5 | 99.0 | 24.7 | 74.3 | 0.6 | 0.4 | embryophyta (n=1614) |
|  | F153 | 98.5 | 93.4 | 5.0 | 1.1 | 0.5 |  |
|  | GP | 99.5 | 97.6 | 1.9 | 0.2 | 0.2 |  |
|  | Yugafu.3C | 96.0 | 81.2 | 14.8 | 1.3 | 2.7 |  |
|  | Yugafu.3H | 91.4 | 81.2 | 10.2 | 1.9 | 6.7 |  |
|  | GL1 | 98.6 | 92.2 | 6.4 | 0.7 | 0.6 |  |
|  | BL_hap1 | 98.4 | 96.3 | 2.0 | 0.5 | 1.1 |  |
|  | BL_hap2 | 99.2 | 95.7 | 3.5 | 0.4 | 0.4 |  |
|  | LY_hap1 | 99.3 | 97.5 | 1.7 | 0.4 | 0.3 |  |
|  | LY_hap2 | 99.4 | 97.6 | 1.8 | 0.4 | 0.2 |  |
|  | YLL_hap1 | 98.9 | 95.5 | 3.4 | 0.4 | 0.6 |  |
|  | YLL_hap2 | 97.7 | 95.7 | 2.0 | 0.6 | 1.7 |  |
|  | CB5 | 98.4 | 19.2 | 79.2 | 1.6 | 0.0 | eukaryota (n=255) |
|  | F153 | 97.6 | 86.3 | 11.4 | 1.6 | 0.8 |  |
|  | GP | 98.8 | 91.0 | 7.8 | 1.2 | 0.0 |  |
|  | Yugafu.3C | 96.9 | 77.6 | 19.2 | 0.4 | 2.7 |  |
|  | Yugafu.3H | 89.4 | 73.7 | 15.7 | 2.4 | 8.2 |  |
|  | GL1 | 98.0 | 87.5 | 10.6 | 1.6 | 0.4 |  |
|  | BL_hap1 | 98.0 | 89.8 | 8.2 | 0.8 | 1.2 |  |
|  | BL_hap2 | 98.0 | 88.6 | 9.4 | 2.0 | 0.0 |  |
|  | LY_hap1 | 98.4 | 90.2 | 8.2 | 1.6 | 0.0 |  |
|  | LY_hap2 | 96.9 | 88.6 | 8.2 | 2.7 | 0.4 |  |
|  | YLL_hap1 | 96.1 | 83.1 | 12.9 | 3.1 | 0.8 |  |
|  | YLL_hap2 | 96.5 | 87.8 | 8.6 | 1.2 | 2.4 |  |
|  | CB5 | 99.3 | 22.8 | 76.5 | 0.5 | 0.2 | viridiplantae (n=425) |
|  | F153 | 98.6 | 93.4 | 5.2 | 1.2 | 0.2 |  |
|  | GP | 99.1 | 98.4 | 0.7 | 0.7 | 0.2 |  |
|  | Yugafu.3C | 95.5 | 79.3 | 16.2 | 1.9 | 2.6 |  |
|  | Yugafu.3H | 90.1 | 80.7 | 9.4 | 2.1 | 7.8 |  |
|  | GL1 | 98.6 | 93.4 | 5.2 | 1.2 | 0.2 |  |
|  | BL_hap1 | 97.9 | 96.7 | 1.2 | 0.7 | 1.4 |  |
|  | BL_hap2 | 98.8 | 96.5 | 2.4 | 0.7 | 0.5 |  |
|  | LY_hap1 | 99.1 | 98.1 | 0.9 | 0.7 | 0.2 |  |
|  | LY_hap2 | 99.1 | 98.1 | 0.9 | 0.7 | 0.2 |  |
|  | YLL_hap1 | 98.8 | 95.5 | 3.3 | 0.7 | 0.5 |  |
|  | YLL_hap2 | 97.9 | 96.5 | 1.4 | 0.7 | 1.4 |  |

**Supplementary Table S7. Repetitive sequence composition of the haplotype-resolved pineapple *F180* genome.** Counts (“Number”), total lengths in megabases (“Length (Mb)”), and proportions of each haplotype (“% of genome”) are reported separately for haplotype A (HA) and haplotype B (HB). Classes are grouped as Class I (retroelements: LTR retrotransposons—Ty1/Copia, Gypsy/DIRS1, retroviral-like, and other/uncertain; and non-LTR retrotransposons—LINEs, SINEs, and PLEs/Penelope), Class II (DNA transposons—hobo/Activator, MULE/MuDR, Tourist/Harbinger, rolling-circle/Helitrons), and “Unclassified” repeats. “Total interspersed repeats” is the sum of Class I, Class II, and Unclassified. Additional repeat categories include small RNAs, simple repeats (microsatellites), and low-complexity sequences. Genome totals (bottom rows) list haplotype assembly size (“Total length”), GC content, and the number and fraction of masked bases (“Bases masked”). Percentages are calculated relative to each haplotype’s total assembly length shown.

| **Class** | | | **HA** | | | | | | | **HB** | | |
| --- | --- | --- | --- | --- | --- | --- | --- | --- | --- | --- | --- | --- |
|  |  |  | **Number** | | **Length (Mb)** | | | **% of genome** | | **Number** | **Length (Mb)** | **% of genome** |
| **Total repeat fraction** | | | 719,269 | | 281.50 | | | 64.97 | | 710,097 | 277.76 | 65.32 |
| **Class I: Retroelements** | | | 57,702 | | 90.81 | | | 20.96 | | 59,238 | 85.72 | 20.16 |
| **LTR retrotransposons** | | | 53,558 | | 87.85 | | | 20.27 | | 51,124 | 81.86 | 19.25 |
| Ty1/Copia | | | 13,254 | | 15.86 | | | 3.66 | | 11,912 | 15.76 | 3.71 |
| Gypsy/DIRS1 | | | 37,768 | | 70.09 | | | 16.18 | | 38,587 | 65.63 | 15.43 |
| Retroviral | | | 1,944 | | 1.54 | | | 0.35 | | 340 | 0.26 | 0.06 |
| Other | | | 592 | | 0.37 | | | 0.08 | | 285 | 0.22 | 0.05 |
| **Non-LTR retrotransposons** | | | 4,144 | | 2.96 | | | 0.68 | | 8,114 | 3.85 | 0.91 |
| LINEs | | | 3,971 | | 2.91 | | | 0.67 | | 6,339 | 3.04 | 0.72 |
| SINEs | | | 173 | | 0.05 | | | 0.01 | | 1,775 | 0.81 | 0.19 |
| PLEs (Penelope) | | | 2,588 | | 0.52 | | | 0.12 | | 2,258 | 0.62 | 0.15 |
| **Class II: DNA transposons** | | | 10,370 | | 4.55 | | | 1.05 | | 12,419 | 5.76 | 1.36 |
| hobo-Activator | | | 1,400 | | 0.68 | | | 0.16 | | 1,403 | 0.71 | 0.17 |
| MULE-MuDR | | | 2,952 | | 2.06 | | | 0.48 | | 1,929 | 1.60 | 0.38 |
| Tourist/Harbinger | | | 1,007 | | 0.58 | | | 0.13 | | 1,100 | 0.68 | 0.16 |
| **Rolling-circles** | | | 1,386 | | 0.92 | | | 0.21 | | 866 | 0.89 | 0.21 |
| **Unclassified** | | | 457,705 | | 170.60 | | | 39.38 | | 449,790 | 172.36 | 40.54 |
| **Total interspersed repeats** | | | 527,163 | | 266.49 | | | **61.51** | | 522,313 | 264.46 | **62.20** |
| **Small RNA** | | | 817 | | 4.70 | | | 1.08 | | 517 | 3.21 | 0.76 |
| **Simple repeats** | | | 170,067 | | 8.21 | | | 1.89 | | 165,979 | 8.01 | 1.88 |
| **Low complexity** | | | 21,222 | | 1.23 | | | 0.28 | | 21288 | 1.18 | 0.28 |
| **Total length** | | 433,271,078 bp | | |  | | |  | 425,204,148 bp | |  |  |
| **GC content** | | 40.09% | | |  | | |  | 39.49% | |  |  |
| **Bases Masked** | | 281,500,679 bp (64.97%) | | |  | | |  | 277,761,655 bp (65.32 %) | |  |  |
|  |  | | |  | |  |  |  |  |  |  |  |

**Supplementary Table S8. Prediction and distribution of ribosomal RNA (rRNA) loci in the haplotype-resolved pineapple *F180* genome.** Counts (“Number”), summed lengths in base pairs (“Total length”), and average sequence lengths are reported for each rRNA type (5S, 18S, 28S, 5.8S) across the whole genome, chromosomes, and unplaced scaffolds of haplotype A (HA) and haplotype B (HB). Most rRNAs are located on scaffolds, with only a subset assigned to chromosomes. Chromosome-level predictions highlight dispersed copies of 5S rRNA as well as localized clusters of 18S, 28S, and 5.8S rRNAs, consistent with expected organization of ribosomal DNA arrays.

| **HA** | | | | | **HB** | | | | |
| --- | --- | --- | --- | --- | --- | --- | --- | --- | --- |
| **Chrom.** | **RNA type** | **Number** | **Total length (bp)** | **Average length (bp)** | **Chrom.** | **RNA type** | **Number** | **Total length (bp)** | **Average length (bp)** |
| **Whole genome** | 5S_rRNA | 1123 | 128,079 | 114.05 | **Whole genome** | 5S_rRNA | 1713 | 195,786 | 114.29 |
|  | 18S_rRNA | 614 | 1,052,838 | 1714.72 |  | 18S_rRNA | 428 | 729,047 | 1703.38 |
|  | 28S_rRNA | 602 | 2,216,721 | 3682.26 |  | 28S_rRNA | 421 | 1,527,131 | 3627.39 |
|  | 5_8S_rRNA | 580 | 89,306 | 153.98 |  | 5_8S_rRNA | 400 | 61,594 | 153.99 |
| AcHAChr01 | 5S_rRNA | 4 | 350 | 87.50 | AcHBChr01 | 5S_rRNA | 2 | 168 | 84.00 |
| AcHAChr02 | 5S_rRNA | 3 | 309 | 103.00 | AcHBChr02 | 5S_rRNA | 3 | 309 | 103.00 |
| AcHAChr03 | 5S_rRNA | 1 | 57 | 57.00 | - |  |  |  |  |
| - |  |  |  |  | AcHBChr04 | 5S_rRNA | 1 | 92 | 92.00 |
| AcHAChr05 | 5S_rRNA | 1 | 102 | 102.00 | AcHBChr05 | 5S_rRNA | 1 | 109 | 109.00 |
| AcHAChr06 | 5S_rRNA | 3 | 312 | 104.00 | AcHBChr06 | 5S_rRNA | 4 | 386 | 96.50 |
| AcHAChr06 | 18S_rRNA | 1 | 599 | 599.00 | AcHBChr06 | 18S_rRNA | 1 | 599 | 599.00 |
| AcHAChr07 | 5S_rRNA | 1 | 82 | 82.00 | AcHBChr07 | 5S_rRNA | 1 | 82 | 82.00 |
| AcHAChr08 | 5S_rRNA | 1 | 55 | 55.00 | AcHBChr08 | 5S_rRNA | 1 | 55 | 55.00 |
| AcHAChr09 | 18S_rRNA | 1 | 774 | 774.00 | AcHBChr09 | 18S_rRNA | 2 | 1,241 | 620.50 |
| AcHAChr09 | 5S_rRNA | 1 | 66 | 66.00 | - |  |  |  |  |
| AcHAChr10 | 5S_rRNA | 1 | 90 | 90.00 | AcHBChr10 | 5S_rRNA | 1 | 90 | 90.00 |
| AcHAChr11 | 18S_rRNA | 1 | 1,270 | 1270.00 | AcHBChr11 | 18S_rRNA | 1 | 1,270 | 1270.00 |
| AcHAChr12 | 5S_rRNA | 2 | 198 | 99.00 | AcHBChr12 | 5S_rRNA | 3 | 306 | 102.00 |
| AcHAChr13 | 18S_rRNA | 1 | 547 | 547.00 | AcHBChr13 | 5S_rRNA | 1 | 56 | 56.00 |
| - |  |  |  |  | AcHBChr13 | 28S_rRNA | 1 | 1,070 | 1070.00 |
| - |  |  |  |  | AcHBChr13 | 18S_rRNA | 1 | 547 | 547.00 |
| - |  |  |  |  | AcHBChr14 | 5S_rRNA | 1 | 90 | 90.00 |
| AcHAChr17 | 5S_rRNA | 1 | 109 | 109.00 | - |  |  |  |  |
| AcHAChr18 | 5S_rRNA | 1097 | 125,652 | 114.54 | AcHBChr18 | 5S_rRNA | 993 | 113,851 | 114.65 |
| AcHAChr21 | 5S_rRNA | 3 | 264 | 88.00 | AcHBChr21 | 5S_rRNA | 2 | 212 | 106.00 |
| AcHAChr25 | 28S_rRNA | 9 | 31,515 | 3501.67 | AcHBChr25 | 28S_rRNA | 3 | 8,654 | 2884.67 |
| AcHAChr25 | 18S_rRNA | 8 | 14,456 | 1807.00 | AcHBChr25 | 18S_rRNA | 2 | 3,614 | 1807.00 |
| AcHAChr25 | 5_8S_rRNA | 8 | 1,232 | 154.00 | AcHBChr25 | 5_8S_rRNA | 2 | 308 | 154.00 |
| Scaffolds | 5S_rRNA | 4 | 433 | 108.25 | Scaffolds | 5S_rRNA | 699 | 79,980 | 114.42 |
|  | 18S_rRNA | 602 | 1,035,192 | 1719.59 |  | 18S_rRNA | 421 | 721,776 | 1714.43 |
|  | 28S_rRNA | 593 | 2,185,206 | 3685.00 |  | 28S_rRNA | 417 | 1,517,407 | 3638.87 |
|  | 5_8S_rRNA | 572 | 88,074 | 153.98 |  | 5_8S_rRNA | 398 | 61,286 | 153.98 |

**Supplementary Table S9. Prediction of tRNA genes in the *F180* genome.** The number, total length, and average length of predicted tRNA genes across each chromosome and unplaced scaffolds are shown for both haplotypes (HA and HB). In total, 1,032 tRNAs were identified in HA and 870 in HB, with average lengths of ~73 bp.

|  | **HA** | | | **HB** | | |
| --- | --- | --- | --- | --- | --- | --- |
| **Chrom.** | **Number** | **Total length** | **Avg length** | **Number** | **Total length** | **Avg length** |
| Chr01 | 44 | 3,248 | 73.82 | 44 | 3,248 | 73.82 |
| Chr02 | 32 | 2,297 | 71.78 | 28 | 1,975 | 70.54 |
| Chr03 | 24 | 1,638 | 68.25 | 24 | 1,638 | 68.25 |
| Chr04 | 49 | 3,548 | 72.41 | 47 | 3,390 | 72.13 |
| Chr05 | 30 | 2,246 | 74.87 | 32 | 2,360 | 73.75 |
| Chr06 | 35 | 2,570 | 73.43 | 35 | 2,568 | 73.37 |
| Chr07 | 31 | 2,246 | 72.45 | 35 | 2,534 | 72.40 |
| Chr08 | 6 | 434 | 72.33 | 6 | 434 | 72.33 |
| Chr09 | 30 | 2,224 | 74.13 | 30 | 2,224 | 74.13 |
| Chr10 | 17 | 1,216 | 71.53 | 17 | 1,210 | 71.18 |
| Chr11 | 33 | 2,394 | 72.55 | 33 | 2,386 | 72.30 |
| Chr12 | 15 | 1,025 | 68.33 | 15 | 1,025 | 68.33 |
| Chr13 | 16 | 1,182 | 73.88 | 18 | 1,308 | 72.67 |
| Chr14 | 19 | 1,352 | 71.16 | 19 | 1,330 | 70.00 |
| Chr15 | 29 | 2,089 | 72.03 | 31 | 2,235 | 72.10 |
| Chr16 | 64 | 4,814 | 75.22 | 62 | 4,628 | 74.65 |
| Chr17 | 22 | 1,630 | 74.09 | 22 | 1,630 | 74.09 |
| Chr18 | 28 | 2,032 | 72.57 | 28 | 2,032 | 72.57 |
| Chr19 | 26 | 1,992 | 76.62 | 26 | 1,992 | 76.62 |
| Chr20 | 34 | 2,284 | 67.18 | 27 | 1,816 | 67.26 |
| Chr21 | 36 | 2,446 | 67.94 | 36 | 2,446 | 67.94 |
| Chr22 | 21 | 1,511 | 71.95 | 21 | 1,511 | 71.95 |
| Chr23 | 24 | 1,824 | 76.00 | 24 | 1,824 | 76.00 |
| Chr24 | 28 | 2,132 | 76.14 | 28 | 2,100 | 75.00 |
| Chr25 | 22 | 1,664 | 75.64 | 22 | 1,664 | 75.64 |
| Scaffolds | 317 | 23,777 | 75.01 | 160 | 12,198 | 76.24 |
| **Total** | **1,032** | **75,815** | **73.46** | **870** | **63,706** | **73.23** |

**Supplementary Table S10. Structural variation between haplotypes A (HA) and B (HB) of the pineapple *F180* genome.** Summary of large-scale structural differences and sequence-level variants identified between the two haplotypes. Structural annotations include syntenic regions, inversions, translocations, duplications, and unaligned sequences. Sequence annotations include SNPs, insertions, deletions, copy number variations, highly diverged regions, and tandem repeats, with counts and total lengths reported separately for HA and HB.

| **#Structural annotations** |  |  |  |
| --- | --- | --- | --- |
| **#Variation_type** | **Count** | **Length_HA** | **Length_HB** |
| Syntenic regions | 489 | 310,358,848 | 310,615,025 |
| Inversions | 74 | 12,269,171 | 15,663,462 |
| Translocations | 750 | 4,399,488 | 4,364,293 |
| Large Insertions (PAVs; >50 bp) | 3,391 | - | 1,638,111 |
| Large Deletions (PAVs; >50 bp) | 3,507 | 2,058,693 | - |
| Duplications (HA) | 254 | 1,138,994 | - |
| Duplications (HB) | 875 | - | 3,484,276 |
| Not aligned (HA) | 1,435 | 86,392,987 | - |
| Not aligned (HB) | 2,092 | - | 78,499,974 |
| **#Sequence annotations** |  |  |  |
| **#Variation_type** | **Count** | **Length_HA** | **Length_HB** |
| SNPs | 1,538,444 | 1,538,444 | 1,538,444 |
| Small Insertions (≤50bp) | 112,393 | - | 540,764 |
| Small Deletions (≤50bp) | 113,639 | 548,267 | - |
| Copygains | 93 | - | 205,567 |
| Copylosses | 106 | 133,074 | - |
| Highly diverged | 18,083 | 119,063,213 | 123,031,097 |
| Tandem repeats | 20 | 3,333 | 4,659 |

**Supplementary Table S11. Average lengths of inversions across haplotypes A (HA) and B (HB) in the pineapple *F180* genome.** The number and mean size of inversions identified per chromosome are shown for each haplotype. Average lengths (bp) were calculated separately for HA and HB, highlighting both conserved and haplotype-specific variation in inversion size across the 25 chromosomes.

| **Chr.** | **Inversion No.** | **Average Length in HA (bp)** | **Average Length in HB (bp)** |
| --- | --- | --- | --- |
| Chr01 | 2 | 2,183 | 2,183 |
| Chr02 | 2 | 75,260 | 64,515 |
| Chr03 | 1 | 602 | 607 |
| Chr04 | 6 | 18,324 | 14,518 |
| Chr05 | 4 | 224,468 | 263,166 |
| Chr06 | 5 | 25,103 | 22,557 |
| Chr07 | 4 | 69,104 | 78,878 |
| Chr08 | 1 | 799 | 803 |
| Chr09 | 3 | 1,232 | 1,230 |
| Chr10 | 4 | 50,957 | 113,128 |
| Chr11 | 4 | 377,616 | 621,488 |
| Chr12 | 6 | 46,268 | 38,536 |
| Chr13 | 5 | 6,230 | 6,922 |
| Chr14 | 3 | 167,616 | 284,279 |
| Chr15 | 3 | 10,387 | 10,203 |
| Chr16 | 1 | 522 | 522 |
| Chr17 | 1 | 464,588 | 524,419 |
| Chr18 | 3 | 6,150 | 6,203 |
| Chr19 | 3 | 14,645 | 17,337 |
| Chr20 | 3 | 435,008 | 440,127 |
| Chr21 | 3 | 80,384 | 76,296 |
| Chr23 | 3 | 5,558 | 5,558 |
| Chr24 | 2 | 3,025,434 | 3,853,247 |
| Chr25 | 2 | 547 | 548 |

**Supplementary Table S12.** **Inversions identified in the pineapple *F180* genome.**
Lengths, genomic positions, and gene content of 74 inversions detected between haplotypes HA and HB. For each inversion, the corresponding chromosome, start and end coordinates, total length, and any breakpoint-associated genes are listed. Genes located fully within the inverted regions are also provided where identified. The total and average inversion lengths are summarized for each haplotype (HA total: 12.43 Mb, HB total: 15.66 Mb). ***Table too big see table S12 in Supplementary Tables file 2.***

**Supplementary Table S13.** **Genes associated with inversions in the pineapple *F180* genome.**
Summary of genes disrupted by inversion breakpoints and those located fully within inverted regions across haplotypes HA and HB. The three largest inversions (Chr24, Chr11, and Chr20) are detailed separately, showing haplotype-specific and allele-paired gene counts. Genes uniquely located within HA or HB inversions but not mirrored in the opposite haplotype are also listed. ***Table too big see table S13 in Supplementary Tables file 2***

| **Supplementary Table S14. Reference genome assemblies of pineapple cultivars and related varieties used for dot-plot comparative analysis.** Each entry provides the cultivar or source, corresponding publication and year, and the type of genome assembly (collapsed, phased, or fully phased T2T). | | | |
| --- | --- | --- | --- |
| **Code in dot-plot** | **Cultivar / source** | **Publication (year)** | **Assembly type** |
| **F153(a)** | *Ananas comosus* ‘Smooth Cayenne’. Latest version (GCA_902162155.2). | Chen et al., Nat. Genet. 2019 [3] | Collapsed |
| **F153(b)** | *Ananas comosus* ‘Smooth Cayenne’. NCBI Refseq version (GCF_001540865.1). | Ming et al., Nat. Genet. 2015 [1] | Collapsed |
| **CB5** | *Ananas comosus* var. *bracteatus* | Chen et al., Nat. Genet. 2019 [3] | Collapsed |
| **GL1** | *Ananas comosus* var. *bracteatus* f. tricolor ‘GL1’ | Feng et al., G3 2022 [4] | Collapsed |
| **MD2** | *Ananas comosus* ‘MD-2’ | Yow et al., Genes 2021 [5] | Phased |
| **BL** | *Ananas comosus* var. *comosus cv. “Ba Li”* (Queen) | Feng et al., JIPB 2024 [2] | Fully phased, T2T |
| **LY** | *Ananas. comosus* var. *erectifolius “Li Ye”* | Feng et al., JIPB 2024 [2] | Fully phased, T2T |
| **YLL** | *Ananas. comosus* var. *microstachys “Yu Ling Long”* | Feng et al., JIPB 2024 [2] | Fully phased, T2T |
| **Yugafu** | *Ananas comosus* (L.) Merr. “Yugafu” (“Cream pineapple” x “HI101”)  (‘Smooth Cayenne’ descendant) | Nashima et al., The Plant Journal 2022 [6] | Fully phased |

**Supplementary Table 15. Allelic gene pairs between haplotypes HA and HB in ‘Smooth Cayenne’ (*F180*).** For each HA gene model, the corresponding HB allele and physical coordinates are listed (format: chromosome:start–end; 1-based, inclusive). Pairs represent 1:1 allelic matches inferred from whole-genome synteny (MCScanX) combined with reciprocal best protein alignments (see Methods). Gene IDs follow the HAxxxxxx.y / HBxxxxxx.y naming scheme. ***Table too big see table S15 in Supplementary Tables file 2***

**Supplementary Table S16. Allelic and haplotype-specific gene counts in the *F180* genome.**
Numbers of allele gene pairs, total genes, and haplotype-specific genes are shown for each chromosome of haplotype A (HA) and haplotype B (HB). Only the longest transcript per locus was considered. "Allele gene number" indicates the count of genes with a one-to-one allelic partner between haplotypes, while "specific gene number" refers to genes detected only in HA or HB. Unanchored scaffolds are listed separately. In total, 19,058 allele gene pairs were identified, alongside 1,196 HA-specific and 1,183 HB-specific genes.

| **HA Chr.** | **HA allele gene No.** | **HA total gene No.*** | **HA specific gene No.** | **HB Chr.** | **HB Allele gene No.** | **HB Total gene No.*** | **HB specific gene No.** |
| --- | --- | --- | --- | --- | --- | --- | --- |
| AcHAChr01 | 977 | 1037 | 60 | AcHBChr01 | 971 | 1045 | 74 |
| AcHAChr02 | 996 | 1091 | 95 | AcHBChr02 | 996 | 1067 | 71 |
| AcHAChr03 | 1009 | 1060 | 51 | AcHBChr03 | 1008 | 1062 | 54 |
| AcHAChr04 | 1063 | 1125 | 62 | AcHBChr04 | 1062 | 1109 | 47 |
| AcHAChr05 | 619 | 644 | 25 | AcHBChr05 | 619 | 657 | 38 |
| AcHAChr06 | 806 | 833 | 27 | AcHBChr06 | 810 | 847 | 37 |
| AcHAChr07 | 760 | 795 | 35 | AcHBChr07 | 755 | 785 | 30 |
| AcHAChr08 | 697 | 742 | 45 | AcHBChr08 | 705 | 749 | 44 |
| AcHAChr09 | 989 | 1055 | 66 | AcHBChr09 | 991 | 1044 | 53 |
| AcHAChr10 | 847 | 900 | 53 | AcHBChr10 | 849 | 893 | 44 |
| AcHAChr11 | 880 | 945 | 65 | AcHBChr11 | 881 | 946 | 65 |
| AcHAChr12 | 913 | 976 | 63 | AcHBChr12 | 917 | 965 | 48 |
| AcHAChr13 | 683 | 723 | 40 | AcHBChr13 | 681 | 740 | 59 |
| AcHAChr14 | 672 | 722 | 50 | AcHBChr14 | 671 | 738 | 67 |
| AcHAChr15 | 747 | 780 | 33 | AcHBChr15 | 748 | 789 | 41 |
| AcHAChr16 | 880 | 925 | 45 | AcHBChr16 | 886 | 923 | 37 |
| AcHAChr17 | 743 | 768 | 25 | AcHBChr17 | 743 | 773 | 30 |
| AcHAChr18 | 689 | 722 | 33 | AcHBChr18 | 690 | 715 | 25 |
| AcHAChr19 | 584 | 628 | 44 | AcHBChr19 | 582 | 634 | 52 |
| AcHAChr20 | 619 | 653 | 34 | AcHBChr20 | 621 | 658 | 37 |
| AcHAChr21 | 612 | 648 | 36 | AcHBChr21 | 612 | 646 | 34 |
| AcHAChr22 | 536 | 567 | 31 | AcHBChr22 | 535 | 585 | 50 |
| AcHAChr23 | 571 | 601 | 30 | AcHBChr23 | 567 | 592 | 25 |
| AcHAChr24 | 580 | 621 | 41 | AcHBChr24 | 574 | 616 | 42 |
| AcHAChr25 | 554 | 589 | 35 | AcHBChr25 | 556 | 577 | 21 |
| Unanchored | 58 | 130 | 72 | Unanchored | 35 | 93 | 58 |
| **In total** | **19,084** | **20,280** | **1,196** | **In total** | **19,065** | **20,248** | **1,183** |
| **Total allele gene pairs** | **19,058** |  |  |  |  |  |  |

**Supplementary Table S17. Number and positions of inversion breakpoint-related genes.**
The table lists HA-specific genes, HB-specific genes, and HA–HB allele gene pairs located across inversion breakpoints. Gene IDs, inversion IDs, genomic positions in HA and HB, and annotated functions are shown. Genes spanning breakpoints in one haplotype but not the other are also included. ***Table too big see table S17 in Supplementary Tables file 2.***

**Supplementary Table S18. log₂ fold-change of allele expression across tissues.**
The table lists allele gene pairs (columns HA_GeneID and HB_GeneID) and their expression differences summarized as log₂FoldChange values for five tissues (T01–T05; see Methods for tissue definitions). log₂FoldChange is computed for HA relative to HB for each tissue; positive values indicate higher expression of the HA allele, negative values indicate higher expression of the HB allele, and 0 indicates no difference. Values are shown for one-to-one allele pairs only. ***Table too big see table S18 in Supplementary Tables file 2.***

**Supplementary Table S19.** **log₂ fold-change of allele expression for genes showing a consistent direction of allelic bias across tissues.** For each one-to-one allele pair (HA_GeneID, HB_GeneID), columns T01_log2FoldChange–T05_log2FoldChange give HA relative to HB (positive = higher HA expression; negative = higher HB; 0 = no difference). Only pairs with the same sign of log₂ fold-change across all five tissues (T01–T05; tissues defined in Materials and Methods) are listed. ***Table too big see table S19 in Supplementary Tables file 2.***

**Supplementary Table S20. log₂-fold change (HA relative to HB) for allele pairs that show expression switching across tissues.** Each row lists a one-to-one allele pair (HA_GeneID, HB_GeneID). Columns T01_log2FoldChange–T05_log2FoldChange give the log₂(HA/HB) value for tissues T01–T05 (defined in Methods). Positive values indicate higher expression of the HA allele; negative values indicate higher expression of the HB allele; 0 indicates no difference. Only allele pairs with at least one tissue favoring each haplotype (i.e., sign changes across tissues) are included. ***Table too big see table S20 in Supplementary Tables file 2.***

**Supplementary Table S21. Allele-specific expressed genes (ASEGs) with transposable element (TE) insertions.** The table lists paired HA_GeneID and HB_GeneID with the presence or absence of TEs in the upstream 2 kb region. The column TE insertion position notes whether the TE occurs in the up_2k region (upstream 2 kb) or is absent ("/").

| **No.** | **HA_GeneID** | **TE insertion position** | **HB_GeneID** | **TE insertion position** |
| --- | --- | --- | --- | --- |
| 1 | HA000126 | up_2k | HB005225 | up_2k |
| 2 | HA000133 | / | HB000134 | up_2k |
| 3 | HA000154 | / | HB000153 | up_2k |
| 4 | HA000319 | / | HB000311 | up_2k |
| 5 | HA000363 | up_2k | HB000362 | up_2k |
| 6 | HA000366 | / | HB000361 | up_2k |
| 7 | HA000397 | up_2k | HB001478 | / |
| 8 | HA000611 | up_2k | HB000616 | up_2k |
| 9 | HA001122 | / | HB001129 | up_2k |
| 10 | HA001123 | up_2k | HB001128 | / |
| 11 | HA001579 | up_2k | HB001563 | / |
| 12 | HA002250 | up_2k | HB002240 | / |
| 13 | HA002331 | / | HB011389 | up_2k |
| 14 | HA002352 | up_2k | HB006602 | / |
| 15 | HA002497 | up_2k | HB002488 | up_2k |
| 16 | HA002520 | / | HB002513 | up_2k |
| 17 | HA003136 | up_2k | HB015140 | / |
| 18 | HA003504 | up_2k | HB003493 | / |
| 19 | HA003507 | / | HB003495 | up_2k |
| 20 | HA003924 | up_2k | HB003898 | / |
| 21 | HA004275 | / | HB004246 | up_2k |
| 22 | HA004565 | / | HB004544 | up_2k |
| 23 | HA004740 | up_2k | HB004717 | / |
| 24 | HA005061 | up_2k | HB005043 | up_2k |
| 25 | HA005150 | / | HB005134 | up_2k |
| 26 | HA005184 | up_2k | HB005170 | / |
| 27 | HA005222 | / | HB005213 | up_2k |
| 28 | HA005282 | up_2k | HB005275 | / |
| 29 | HA005323 | / | HB015196 | up_2k |
| 30 | HA005726 | / | HB005721 | up_2k |
| 31 | HA005981 | / | HB005965 | up_2k |
| 32 | HA006000 | / | HB005984 | up_2k |
| 33 | HA006159 | up_2k | HB009634 | / |
| 34 | HA006424 | up_2k | HB006410 | / |
| 35 | HA006884 | up_2k | HB006864 | up_2k |
| 36 | HA007216 | up_2k | HB007208 | / |
| 37 | HA007453 | / | HB007447 | up_2k |
| 38 | HA007856 | / | HB007845 | up_2k |
| 39 | HA008149 | / | HB008134 | up_2k |
| 40 | HA008161 | up_2k | HB008153 | / |
| 41 | HA008261 | / | HB008245 | up_2k |
| 42 | HA009342 | / | HB009320 | up_2k |
| 43 | HA009386 | / | HB009366 | up_2k |
| 44 | HA009500 | / | HB009473 | up_2k |
| 45 | HA009870 | up_2k | HB009832 | up_2k |
| 46 | HA011256 | up_2k | HB011230 | / |
| 47 | HA011283 | up_2k | HB006745 | / |
| 48 | HA011287 | / | HB011262 | up_2k |
| 49 | HA011453 | / | HB011433 | up_2k |
| 50 | HA012025 | up_2k | HB012005 | up_2k |
| 51 | HA012194 | up_2k | HB012186 | / |
| 52 | HA012291 | up_2k | HB012286 | / |
| 53 | HA012706 | / | HB012706 | up_2k |
| 54 | HA012732 | / | HB012733 | up_2k |
| 55 | HA012891 | up_2k | HB011451 | up_2k |
| 56 | HA012914 | up_2k | HB012918 | up_2k |
| 57 | HA012915 | up_2k | HB012919 | / |
| 58 | HA013455 | up_2k | HB013460 | / |
| 59 | HA013587 | / | HB013589 | up_2k |
| 60 | HA013843 | / | HB013855 | up_2k |
| 61 | HA015127 | up_2k | HB015141 | up_2k |
| 62 | HA015147 | / | HB015153 | up_2k |
| 63 | HA015220 | up_2k | HB015228 | / |
| 64 | HA015557 | / | HB015567 | up_2k |
| 65 | HA016004 | up_2k | HB020200 | / |
| 66 | HA016035 | up_2k | HB016049 | / |
| 67 | HA016110 | / | HB016121 | up_2k |
| 68 | HA016117 | up_2k | HB015224 | / |
| 69 | HA016178 | up_2k | HB016193 | / |
| 70 | HA016179 | up_2k | HB016192 | / |
| 71 | HA016245 | / | HB016258 | up_2k |
| 72 | HA016246 | up_2k | HB016257 | / |
| 73 | HA017207 | up_2k | HB017228 | / |
| 74 | HA017316 | up_2k | HB017328 | / |
| 75 | HA017671 | / | HB017687 | up_2k |
| 76 | HA017720 | up_2k | HB011439 | / |
| 77 | HA017829 | / | HB017844 | up_2k |
| 78 | HA017929 | up_2k | HB002382 | / |
| 79 | HA017937 | / | HB017952 | up_2k |
| 80 | HA018003 | up_2k | HB018042 | / |
| 81 | HA018290 | / | HB018323 | up_2k |
| 82 | HA018737 | / | HB007254 | up_2k |
| 83 | HA019025 | / | HB019011 | up_2k |
| 84 | HA019071 | up_2k | HB009260 | up_2k |
| 85 | HA019153 | / | HB018688 | up_2k |
| 86 | HA020248 | / | HB009767 | up_2k |

**Supplementary Table S22. Proportion of markers aligning to unique and multiple positions within pineapple reference genomes.**

|  | **Number of marker alignments** | | | | | |
| --- | --- | --- | --- | --- | --- | --- |
| **Variety** | **0** | **1** | **2** | **3** | **4** | **5+** |
| ***F153(a)*** | 0.128 | 0.745 | 0.091 | 0.018 | 0.006 | 0.012 |
| ***MD2 P0*** | 0.111 | 0.545 | 0.281 | 0.025 | 0.015 | 0.022 |
| ***MD2 P1*** | 0.110 | 0.565 | 0.266 | 0.023 | 0.014 | 0.021 |
| ***SC F180 HA*** | 0.130 | 0.789 | 0.048 | 0.013 | 0.007 | 0.014 |
| ***SC F180 HB*** | 0.131 | 0.791 | 0.046 | 0.012 | 0.006 | 0.015 |
|  |  |  |  |  |  |  |
| ***Ratio F153(a):SC F180*** | 0.981 | 0.943 | 1.936 | 1.440 | 0.923 | 0.828 |
| ***Ratio MD2:SC F180*** | 0.847 | 0.703 | 5.819 | 1.920 | 2.231 | 1.483 |

**Supplementary Table S23 Single Crossover (SCO) Counts and Frequencies in Inverted and Flanking Regions, with Bonferroni-Adjusted P-Values for Pairwise Comparisons.** P values were Bonferroni-adjusted. Within, upstream and downstream refer to the inverted region and its upstream and downstream flanking regions, respectively.

| **Inversion** | | **Haplotype** | | **Region** | **SCO count** | **Marker count** | **SCO / Marker** | **P-value (Flanking vs. Inversion)** | **P-value (Upstream vs. Downstream)** |
| --- | --- | --- | --- | --- | --- | --- | --- | --- | --- |
|  | Inv553 | HA | | Upstream | 12 | 31 | 0.387 | 0.012 | 0.432 |
|  |  |  | | Within | 1 | 27 | 0.037 | — | — |
|  |  |  | | Downstream | 22 | 37 | 0.595 | 4.5 × 10⁻⁵ | — |
|  | Inv553 | HB | | Upstream | 10 | 23 | 0.435 | 0.013 | 1 |
|  |  |  | | Within | 1 | 24 | 0.042 | — | — |
|  |  |  | | Downstream | 15 | 30 | 0.500 | 0.0023 | — |
|  |  |  | |  |  |  |  |  | — |
|  | Inv560 | HA | | Within | 12 | 40 | 0.300 | — | — |
|  |  |  | | Flanking | 40 | 91 | 0.440 | 0.19 | — |
|  | Inv560 | HB | | Within | 3 | 30 | 0.100 | — | — |
|  |  | |  | Flanking | 34 | 86 | 0.395 | 0.0058 | — |

**Supplementary Table S24. Full list of nucleotide-binding leucine-rich repeat (NLR) genes identified in the *F180* haplotype A (HA) assembly.** The table provides the chromosome location (Chromosome, Start, End), transcriptional strand orientation (Strand), gene identifiers (GeneID), and subgroup classification. Subgroups include CNL (CC-NBS-LRR), NL (NBS-LRR without CC/TIR), NBS (NBS domain only), CN (CC-NBS), RNL (RPW8-NBS-LRR), TN (TIR-NBS), and TX (TIR-domain containing).

| NO. | Chromosome | Start | End | Strand | GeneID | Subgroup |
| --- | --- | --- | --- | --- | --- | --- |
| 1 | AcHAChr01 | 267,323 | 268,759 | + | HA000030 | NBS |
| 2 | AcHAChr01 | 889,601 | 892,443 | - | HA000098 | CNL |
| 3 | AcHAChr01 | 1,550,634 | 1,554,050 | - | HA000147 | NL |
| 4 | AcHAChr01 | 1,560,770 | 1,572,787 | - | HA000148 | CNL |
| 5 | AcHAChr01 | 1,585,236 | 1,588,685 | + | HA000151 | CNL |
| 6 | AcHAChr01 | 1,594,265 | 1,597,795 | - | HA000152 | CNL |
| 7 | AcHAChr01 | 1,700,398 | 1,704,345 | - | HA000162 | NL |
| 8 | AcHAChr01 | 1,799,560 | 1,802,828 | - | HA000173 | CNL |
| 9 | AcHAChr01 | 1,811,470 | 1,814,796 | - | HA000174 | CNL |
| 10 | AcHAChr01 | 1,816,259 | 1,819,513 | - | HA000175 | CNL |
| 11 | AcHAChr01 | 20,226,814 | 20,230,453 | + | HA000927 | RNL |
| 12 | AcHAChr02 | 18,302,164 | 18,305,828 | - | HA001845 | CNL |
| 13 | AcHAChr02 | 19,701,499 | 19,704,465 | + | HA002014 | TN |
| 14 | AcHAChr02 | 19,762,661 | 19,766,416 | + | HA002020 | CNL |
| 15 | AcHAChr03 | 842,954 | 846,199 | + | HA002239 | CNL |
| 16 | AcHAChr03 | 859,447 | 862,638 | + | HA002240 | CNL |
| 17 | AcHAChr03 | 867,074 | 867,877 | + | HA002241 | CN |
| 18 | AcHAChr03 | 884,544 | 887,831 | + | HA002242 | CNL |
| 19 | AcHAChr03 | 905,566 | 908,853 | + | HA002243 | CNL |
| 20 | AcHAChr03 | 916,244 | 928,947 | + | HA002244 | CNL |
| 21 | AcHAChr03 | 933,952 | 937,149 | + | HA002245 | CNL |
| 22 | AcHAChr03 | 1,435,134 | 1,437,668 | + | HA002285 | CNL |
| 23 | AcHAChr03 | 1,440,116 | 1,442,878 | - | HA002286 | CNL |
| 24 | AcHAChr03 | 2,345,293 | 2,348,913 | + | HA002352 | NL |
| 25 | AcHAChr03 | 2,588,063 | 2,590,753 | - | HA002367 | CNL |
| 26 | AcHAChr03 | 3,069,048 | 3,072,080 | - | HA002388 | TN |
| 27 | AcHAChr03 | 19,197,085 | 19,199,608 | + | HA003045 | TX |
| 28 | AcHAChr04 | 2,442,115 | 2,445,384 | - | HA003504 | NL |
| 29 | AcHAChr04 | 2,455,493 | 2,464,135 | - | HA003505 | NL |
| 30 | AcHAChr04 | 2,485,001 | 2,513,596 | - | HA003506 | NL |
| 31 | AcHAChr04 | 2,528,662 | 2,531,256 | - | HA003508 | CNL |
| 32 | AcHAChr04 | 2,666,053 | 2,672,425 | + | HA003521 | CNL |
| 33 | AcHAChr04 | 2,695,927 | 2,699,931 | + | HA003522 | CNL |
| 34 | AcHAChr04 | 2,713,622 | 2,717,213 | + | HA003523 | CNL |
| 35 | AcHAChr04 | 2,733,045 | 2,737,826 | + | HA003525 | NL |
| 36 | AcHAChr04 | 2,741,633 | 2,742,418 | + | HA003526 | NBS |
| 37 | AcHAChr04 | 2,799,552 | 2,804,397 | + | HA003527 | NL |
| 38 | AcHAChr04 | 3,149,679 | 3,152,906 | + | HA003549 | CNL |
| 39 | AcHAChr04 | 3,174,356 | 3,177,583 | + | HA003551 | CNL |
| 40 | AcHAChr04 | 3,235,585 | 3,238,809 | + | HA003552 | CNL |
| 41 | AcHAChr04 | 3,240,289 | 3,241,557 | + | HA003553 | CN |
| 42 | AcHAChr04 | 3,328,067 | 3,331,358 | + | HA003558 | NBS |
| 43 | AcHAChr04 | 3,541,314 | 3,545,116 | + | HA003575 | CNL |
| 44 | AcHAChr04 | 3,622,965 | 3,626,363 | + | HA003582 | CNL |
| 45 | AcHAChr04 | 3,896,999 | 3,901,100 | - | HA003598 | NL |
| 46 | AcHAChr04 | 10,473,011 | 10,473,957 | - | HA003696 | TX |
| 47 | AcHAChr06 | 17,343,295 | 17,346,735 | + | HA005679 | NL |
| 48 | AcHAChr06 | 17,355,575 | 17,358,544 | + | HA005680 | NL |
| 49 | AcHAChr06 | 17,605,248 | 17,607,598 | - | HA005713 | CN |
| 50 | AcHAChr06 | 17,616,199 | 17,630,445 | - | HA005714 | CNL |
| 51 | AcHAChr08 | 281,538 | 285,266 | - | HA006613 | CNL |
| 52 | AcHAChr08 | 319,410 | 323,453 | - | HA006617 | NL |
| 53 | AcHAChr08 | 516,957 | 520,307 | + | HA006631 | CNL |
| 54 | AcHAChr08 | 530,035 | 533,694 | + | HA006632 | CNL |
| 55 | AcHAChr08 | 632,200 | 633,714 | - | HA006633 | CN |
| 56 | AcHAChr08 | 663,070 | 666,726 | - | HA006634 | CNL |
| 57 | AcHAChr08 | 14,087,042 | 14,088,517 | - | HA006898 | CN |
| 58 | AcHAChr09 | 17,535,573 | 17,539,214 | + | HA008352 | CN |
| 59 | AcHAChr10 | 16,287,368 | 16,291,171 | + | HA009170 | CNL |
| 60 | AcHAChr10 | 16,346,963 | 16,350,796 | + | HA009182 | CNL |
| 61 | AcHAChr11 | 1,673,197 | 1,678,345 | - | HA009505 | NL |
| 62 | AcHAChr11 | 1,680,079 | 1,683,831 | - | HA009506 | NL |
| 63 | AcHAChr11 | 1,714,700 | 1,721,011 | - | HA009508 | CNL |
| 64 | AcHAChr11 | 1,917,639 | 1,920,551 | + | HA009527 | TN |
| 65 | AcHAChr12 | 2,114,673 | 2,118,683 | - | HA010498 | CNL |
| 66 | AcHAChr12 | 10,360,520 | 10,363,957 | - | HA010691 | CNL |
| 67 | AcHAChr12 | 10,361,911 | 10,363,957 | - | HA010692 | NL |
| 68 | AcHAChr12 | 10,506,654 | 10,510,098 | - | HA010694 | NL |
| 69 | AcHAChr12 | 15,374,541 | 15,377,096 | + | HA011080 | TX |
| 70 | AcHAChr13 | 282,776 | 289,786 | - | HA011228 | NL |
| 71 | AcHAChr13 | 297,661 | 301,764 | + | HA011229 | NL |
| 72 | AcHAChr13 | 311,292 | 316,747 | + | HA011230 | CNL |
| 73 | AcHAChr13 | 326,196 | 349,518 | + | HA011231 | CNL |
| 74 | AcHAChr13 | 355,597 | 389,477 | + | HA011232 | CNL |
| 75 | AcHAChr13 | 396,395 | 400,492 | + | HA011233 | CNL |
| 76 | AcHAChr13 | 648,821 | 652,822 | - | HA011255 | NL |
| 77 | AcHAChr13 | 661,103 | 665,085 | - | HA011256 | CNL |
| 78 | AcHAChr13 | 1,202,643 | 1,205,462 | - | HA011273 | NL |
| 79 | AcHAChr13 | 11,374,283 | 11,376,175 | + | HA011427 | CNL |
| 80 | AcHAChr14 | 915,103 | 915,927 | + | HA012014 | CN |
| 81 | AcHAChr14 | 925,448 | 928,252 | - | HA012016 | NL |
| 82 | AcHAChr15 | 818,466 | 821,186 | + | HA012720 | NL |
| 83 | AcHAChr15 | 907,072 | 910,008 | - | HA012728 | CNL |
| 84 | AcHAChr15 | 916,688 | 919,624 | - | HA012729 | CNL |
| 85 | AcHAChr16 | 1,893,948 | 1,897,013 | + | HA013575 | NL |
| 86 | AcHAChr16 | 1,909,529 | 1,911,112 | + | HA013576 | NBS |
| 87 | AcHAChr16 | 12,420,619 | 12,423,078 | - | HA013901 | TX |
| 88 | AcHAChr18 | 12,816,624 | 12,819,542 | + | HA015546 | CNL |
| 89 | AcHAChr19 | 2,061,804 | 2,137,331 | + | HA016036 | NL |
| 90 | AcHAChr19 | 2,133,630 | 2,198,420 | + | HA016041 | CNL |
| 91 | AcHAChr19 | 2,257,179 | 2,260,889 | + | HA016049 | CNL |
| 92 | AcHAChr19 | 2,577,672 | 2,581,394 | - | HA016061 | CNL |
| 93 | AcHAChr20 | 12,527,565 | 12,530,807 | - | HA016875 | NL |
| 94 | AcHAChr20 | 14,018,118 | 14,042,254 | + | HA017092 | CNL |
| 95 | AcHAChr21 | 1,383,478 | 1,386,333 | - | HA017203 | NL |
| 96 | AcHAChr21 | 1,391,985 | 1,392,806 | - | HA017204 | NL |
| 97 | AcHAChr21 | 1,393,007 | 1,393,702 | - | HA017205 | CN |
| 98 | AcHAChr22 | 1,433,623 | 1,436,397 | + | HA017866 | NL |
| 99 | AcHAChr25 | 9,992,876 | 9,996,226 | + | HA019841 | CNL |

**Supplementary Table S25. Full list of nucleotide-binding leucine-rich repeat (NLR) genes identified in the *F180* haplotype B (HB) assembly.** The table provides the chromosome location (Chromosome, Start, End), transcriptional strand orientation (Strand), gene identifiers (GeneID), and subgroup classification. Subgroups include CNL (CC-NBS-LRR), NL (NBS-LRR without CC/TIR), NBS (NBS domain only), CN (CC-NBS), RNL (RPW8-NBS-LRR), TN (TIR-NBS), and TX (TIR-domain containing).

| **NO.** | **Chromosome** | **Start** | **End** | **Strand** | **GeneID** | **Subgroup** |
| --- | --- | --- | --- | --- | --- | --- |
| 1 | AcHBChr01 | 256,510 | 257,946 | + | HB000031 | NBS |
| 2 | AcHBChr01 | 910,833 | 913,675 | - | HB000102 | CNL |
| 3 | AcHBChr01 | 1,280,120 | 1,280,773 | - | HB000145 | NBS |
| 4 | AcHBChr01 | 1,293,229 | 1,296,678 | + | HB000148 | CNL |
| 5 | AcHBChr01 | 1,302,331 | 1,305,861 | - | HB000149 | CNL |
| 6 | AcHBChr01 | 1,321,158 | 1,324,109 | + | HB000150 | NL |
| 7 | AcHBChr01 | 1,440,467 | 1,472,117 | - | HB000161 | NL |
| 8 | AcHBChr01 | 1,569,372 | 1,588,933 | - | HB000174 | CNL |
| 9 | AcHBChr01 | 15,517,070 | 15,519,820 | - | HB000515 | NL |
| 10 | AcHBChr01 | 15,641,987 | 15,644,746 | - | HB000516 | NL |
| 11 | AcHBChr01 | 15,685,612 | 15,688,356 | - | HB000517 | NL |
| 12 | AcHBChr01 | 19,646,255 | 19,649,890 | + | HB000932 | RNL |
| 13 | AcHBChr02 | 17,272,459 | 17,276,118 | - | HB001836 | CNL |
| 14 | AcHBChr02 | 17,279,201 | 17,282,503 | - | HB001837 | NL |
| 15 | AcHBChr02 | 18,145,721 | 18,149,922 | - | HB001949 | CNL |
| 16 | AcHBChr02 | 18,615,507 | 18,618,482 | + | HB002005 | TN |
| 17 | AcHBChr02 | 18,676,772 | 18,680,527 | + | HB002011 | CNL |
| 18 | AcHBChr02 | 18,682,540 | 18,683,298 | + | HB002012 | NBS |
| 19 | AcHBChr03 | 902,450 | 905,410 | + | HB002225 | CNL |
| 20 | AcHBChr03 | 910,007 | 913,225 | + | HB002227 | CNL |
| 21 | AcHBChr03 | 917,551 | 920,745 | + | HB002228 | CNL |
| 22 | AcHBChr03 | 925,065 | 925,985 | + | HB002229 | CN |
| 23 | AcHBChr03 | 929,857 | 930,282 | + | HB002230 | NBS |
| 24 | AcHBChr03 | 942,648 | 945,935 | + | HB002232 | CNL |
| 25 | AcHBChr03 | 959,273 | 962,464 | + | HB002233 | CNL |
| 26 | AcHBChr03 | 967,859 | 971,074 | + | HB002234 | CNL |
| 27 | AcHBChr03 | 980,678 | 983,875 | + | HB002235 | CNL |
| 28 | AcHBChr03 | 1,428,510 | 1,431,029 | + | HB002273 | CNL |
| 29 | AcHBChr03 | 1,433,482 | 1,436,244 | - | HB002274 | CNL |
| 30 | AcHBChr03 | 2,566,322 | 2,568,619 | - | HB002354 | NL |
| 31 | AcHBChr03 | 3,093,564 | 3,096,596 | - | HB002377 | TN |
| 32 | AcHBChr03 | 19,047,936 | 19,050,458 | + | HB003031 | TX |
| 33 | AcHBChr04 | 2,484,587 | 2,488,207 | - | HB003493 | NL |
| 34 | AcHBChr04 | 2,530,332 | 2,587,087 | - | HB003494 | NL |
| 35 | AcHBChr04 | 2,615,001 | 2,620,029 | - | HB003496 | CNL |
| 36 | AcHBChr04 | 2,757,323 | 2,761,885 | + | HB003507 | CNL |
| 37 | AcHBChr04 | 2,770,573 | 2,775,933 | + | HB003508 | CNL |
| 38 | AcHBChr04 | 2,790,087 | 2,794,859 | + | HB003510 | NL |
| 39 | AcHBChr04 | 2,798,666 | 2,799,451 | + | HB003511 | NBS |
| 40 | AcHBChr04 | 2,824,163 | 2,829,062 | + | HB003512 | NL |
| 41 | AcHBChr04 | 3,176,436 | 3,179,663 | + | HB003532 | CNL |
| 42 | AcHBChr04 | 3,227,211 | 3,230,444 | + | HB003533 | NL |
| 43 | AcHBChr04 | 3,255,310 | 3,256,500 | + | HB003534 | CN |
| 44 | AcHBChr04 | 3,259,867 | 3,263,091 | + | HB003535 | CNL |
| 45 | AcHBChr04 | 3,352,113 | 3,354,029 | + | HB003540 | NBS |
| 46 | AcHBChr04 | 3,561,475 | 3,565,278 | + | HB003557 | CNL |
| 47 | AcHBChr04 | 3,640,748 | 3,644,151 | + | HB003564 | CNL |
| 48 | AcHBChr04 | 3,874,978 | 3,877,680 | + | HB003581 | CNL |
| 49 | AcHBChr05 | 13,511,486 | 13,514,122 | + | HB004573 | TN |
| 50 | AcHBChr06 | 15,864,024 | 15,867,464 | + | HB005675 | NL |
| 51 | AcHBChr06 | 15,876,307 | 15,879,276 | + | HB005677 | NL |
| 52 | AcHBChr06 | 16,115,839 | 16,119,697 | - | HB005709 | CNL |
| 53 | AcHBChr08 | 240,652 | 244,410 | - | HB006598 | CNL |
| 54 | AcHBChr08 | 259,403 | 292,508 | - | HB006599 | NL |
| 55 | AcHBChr08 | 303,845 | 307,900 | - | HB006600 | NL |
| 56 | AcHBChr08 | 326,466 | 330,056 | - | HB006601 | NL |
| 57 | AcHBChr08 | 355,866 | 359,504 | - | HB006602 | CNL |
| 58 | AcHBChr08 | 565,345 | 568,992 | + | HB006616 | CNL |
| 59 | AcHBChr08 | 586,381 | 629,626 | + | HB006617 | CNL |
| 60 | AcHBChr08 | 688,705 | 692,361 | - | HB006619 | CNL |
| 61 | AcHBChr08 | 13,913,975 | 13,915,429 | - | HB006888 | CN |
| 62 | AcHBChr09 | 17,470,421 | 17,472,970 | + | HB008133 | TX |
| 63 | AcHBChr09 | 17,475,142 | 17,477,787 | + | HB008134 | TX |
| 64 | AcHBChr09 | 19,124,266 | 19,127,907 | + | HB008337 | CN |
| 65 | AcHBChr11 | 1,594,091 | 1,600,429 | - | HB009477 | CNL |
| 66 | AcHBChr11 | 1,782,988 | 1,785,912 | + | HB009495 | TN |
| 67 | AcHBChr12 | 2,147,428 | 2,151,412 | - | HB010469 | CNL |
| 68 | AcHBChr12 | 11,774,806 | 11,778,243 | - | HB010666 | CNL |
| 69 | AcHBChr12 | 11,920,942 | 11,924,386 | - | HB010669 | NL |
| 70 | AcHBChr12 | 16,832,359 | 16,834,914 | + | HB011040 | TX |
| 71 | AcHBChr13 | 229,076 | 232,450 | - | HB011192 | NL |
| 72 | AcHBChr13 | 249,078 | 272,710 | + | HB011193 | NL |
| 73 | AcHBChr13 | 280,971 | 284,776 | + | HB011194 | CNL |
| 74 | AcHBChr13 | 289,994 | 353,949 | + | HB011195 | CNL |
| 75 | AcHBChr13 | 336,773 | 340,848 | + | HB011197 | CNL |
| 76 | AcHBChr13 | 591,580 | 595,810 | - | HB011217 | CNL |
| 77 | AcHBChr13 | 610,916 | 615,117 | - | HB011218 | CNL |
| 78 | AcHBChr13 | 627,576 | 631,165 | - | HB011219 | CNL |
| 79 | AcHBChr13 | 636,281 | 640,317 | - | HB011220 | NL |
| 80 | AcHBChr13 | 667,615 | 668,364 | - | HB011221 | CN |
| 81 | AcHBChr13 | 680,493 | 684,477 | - | HB011222 | CNL |
| 82 | AcHBChr13 | 698,096 | 698,803 | - | HB011223 | NBS |
| 83 | AcHBChr13 | 721,277 | 724,385 | - | HB011224 | CNL |
| 84 | AcHBChr13 | 729,495 | 733,531 | - | HB011225 | NL |
| 85 | AcHBChr13 | 760,817 | 763,915 | - | HB011226 | CNL |
| 86 | AcHBChr13 | 776,684 | 779,407 | - | HB011227 | CNL |
| 87 | AcHBChr13 | 790,562 | 791,269 | - | HB011228 | NBS |
| 88 | AcHBChr13 | 815,537 | 818,287 | - | HB011229 | CNL |
| 89 | AcHBChr13 | 837,328 | 841,302 | - | HB011230 | CNL |
| 90 | AcHBChr13 | 896,956 | 899,640 | + | HB011233 | CNL |
| 91 | AcHBChr13 | 1,348,341 | 1,351,148 | - | HB011251 | CNL |
| 92 | AcHBChr13 | 11,947,934 | 11,951,398 | + | HB011407 | CNL |
| 93 | AcHBChr14 | 856,362 | 859,217 | - | HB011993 | NL |
| 94 | AcHBChr14 | 862,533 | 865,322 | + | HB011994 | NL |
| 95 | AcHBChr14 | 877,635 | 880,439 | - | HB011995 | NL |
| 96 | AcHBChr15 | 795,947 | 798,727 | + | HB012718 | NL |
| 97 | AcHBChr15 | 812,442 | 815,162 | + | HB012719 | NL |
| 98 | AcHBChr15 | 893,498 | 896,434 | - | HB012727 | CNL |
| 99 | AcHBChr15 | 893,498 | 902,743 | - | HB012728 | CNL |
| 100 | AcHBChr15 | 914,720 | 916,150 | - | HB012730 | CN |
| 101 | AcHBChr16 | 1,861,288 | 1,864,353 | + | HB013579 | NL |
| 102 | AcHBChr16 | 12,124,209 | 12,126,674 | - | HB013909 | TX |
| 103 | AcHBChr18 | 12,884,518 | 12,887,436 | + | HB015556 | CNL |
| 104 | AcHBChr19 | 2,156,345 | 2,162,702 | + | HB016055 | CNL |
| 105 | AcHBChr19 | 2,474,923 | 2,478,612 | - | HB016067 | CNL |
| 106 | AcHBChr20 | 12,075,155 | 12,078,406 | + | HB016920 | NL |
| 107 | AcHBChr20 | 13,446,706 | 13,460,996 | + | HB017106 | CNL |
| 108 | AcHBChr21 | 1,204,514 | 1,208,731 | + | HB017216 | NL |
| 109 | AcHBChr22 | 830,411 | 833,923 | + | HB017850 | NL |
| 110 | AcHBChr22 | 840,332 | 844,195 | + | HB017851 | NL |
| 111 | AcHBChr22 | 1,517,945 | 1,520,719 | + | HB017893 | NL |
| 112 | AcHBChr25 | 9,731,360 | 9,734,716 | + | HB019846 | CNL |
| 113 | AcHBChr25 | 9,737,179 | 9,740,529 | + | HB019847 | CNL |

**Supplementary Table S26.** The identification and classification of NLR genes between pineapple haplotype genomes.

| **Genome** | **Coding genes** | **NBS** | **CNL** | **CN** | **TN** | **NL** | **TX** | **RNL** | **Total** |
| --- | --- | --- | --- | --- | --- | --- | --- | --- | --- |
| HA | 20,280 | 4 | 51 | 8 | 3 | 28 | 4 | 1 | 99 |
| Percentage | / | 4.04% | 51.52% | 8.08% | 3.03% | 28.28% | 1.01% | 4.04% | 100% |
|  |  |  |  |  |  |  |  |  |  |
| HB | 20,248 | 8 | 56 | 6 | 4 | 33 | 5 | 1 | 113 |
| Percentage | / | 7.08% | 49.56% | 5.31% | 3.54% | 29.20% | 0.88% | 4.42% | 100% |

**Supplementary Table S27. List of NLR allelic gene pairs between *F180* haplotype A (HA) and haplotype B (HB) assemblies.** Each row shows either one-to-one allelic relationships or cases where a gene in one haplotype corresponds to multiple allelic counterparts in the other haplotype. Columns include the table index (NO.), the HA gene identifier (HA_GeneID), and the HB gene identifier (HB_GeneID). Multiple allelic relationships are separated by commas within a cell.

| **NO.** | **HA_GeneID** | **HB_GeneID** | **NO.** | **HA_GeneID** | **HB_GeneID** |
| --- | --- | --- | --- | --- | --- |
| 1 | HA000030 | HB000031 | 40 | HA005714 | HB005709 |
| 2 | HA000098 | HB000102 | 41 | HA006617 | HB006599 |
| 3 | HA000151 | HB000148 | 42 | HA002352 | HB006602 |
| 4 | HA000152 | HB000149 | 43 | HA006632 | HB006616 |
| 5 | HA000162 | HB000161 | 44 | HA006631 | HB006617 |
| 6 | HA000174 | HB000174 | 45 | HA006634 | HB006619 |
| 7 | HA000927 | HB000932 | 46 | HA006898 | HB006888 |
| 8 | HA001845 | HB001836 | 47 | HA008352 | HB008337 |
| 9 | HA002014 | HB002005 | 48 | HA009508 | HB009477 |
| 10 | HA002020 | HB002011 | 49 | HA009527 | HB009495 |
| 11 | HA002241 | HB002229 | 50 | HA010498 | HB010469 |
| 12 | HA002239 | HB002232 | 51 | HA010691 | HB010666 |
| 13 | HA002240 | HB002233 | 52 | HA010692 | HB010668 |
| 14 | HA002245 | HB002235 | 53 | HA010694 | HB010669 |
| 15 | HA002285 | HB002273 | 54 | HA011080 | HB011040 |
| 16 | HA002286 | HB002274 | 55 | HA011228 | HB011192 |
| 17 | HA002367 | HB002354 | 56 | HA011229 | HB011193 |
| 18 | HA002388 | HB002377 | 57 | HA011233 | HB011195 |
| 19 | HA003045 | HB003031 | 58 | HA011232,HA011255 | HB011197 |
| 20 | HA003504 | HB003493 | 59 | HA011232,HA011255 | HB011217 |
| 21 | HA003505,HA003521 | HB003507 | 60 | HA011232 | HB011197,HB011217 |
| 22 | HA003505,HA003521 | HB003494 | 61 | HA011255 | HB011197,HB011217 |
| 23 | HA003505 | HB003494,HB003507 | 62 | HA011256 | HB011230 |
| 24 | HA003521 | HB003494,HB003507 | 63 | HA011273 | HB011251 |
| 25 | HA003506,HA003523 | HB003496 | 64 | HA011427 | HB011407 |
| 26 | HA003506,HA003523 | HB003512 | 65 | HA012016 | HB011995 |
| 27 | HA003506 | HB003496,HB003512 | 66 | HA012720 | HB012718 |
| 28 | HA003523 | HB003496,HB003512 | 67 | HA012729 | HB012727 |
| 29 | HA003522 | HB003508 | 68 | HA012728 | HB012728 |
| 30 | HA003525 | HB003510 | 69 | HA013575 | HB013579 |
| 31 | HA003526 | HB003511 | 70 | HA013901 | HB013909 |
| 32 | HA003549 | HB003532 | 71 | HA015546 | HB015556 |
| 33 | HA003553 | HB003533 | 72 | HA016041 | HB016055 |
| 34 | HA003552 | HB003535 | 73 | HA016036 | HB016067 |
| 35 | HA003558 | HB003540 | 74 | HA016875 | HB016920 |
| 36 | HA003575 | HB003557 | 75 | HA017092 | HB017106 |
| 37 | HA003582 | HB003564 | 76 | HA011231 | HB017216 |
| 38 | HA005679 | HB005675 | 77 | HA017866 | HB017893 |
| 39 | HA005680 | HB005677 | 78 | HA019841 | HB019847 |

**Supplementary Table S28. Haplotype-specific NLR genes identified in the *F180* genome.** Genes listed in the HA_GeneID column are specific to haplotype A, while those in the HB_GeneID column are specific to haplotype B. Where no counterpart is present in the opposite haplotype, the entry is left blank.

| **HA_GeneID** | **HB_GeneID** |
| --- | --- |
| HA000147 | HB000145 |
| HA000148 | HB000150 |
| HA000173 | HB000515 |
| HA000175 | HB000516 |
| HA002242 | HB000517 |
| HA002243 | HB001837 |
| HA002244 | HB001949 |
| HA003508 | HB002012 |
| HA003527 | HB002225 |
| HA003551 | HB002227 |
| HA003598 | HB002228 |
| HA003696 | HB002230 |
| HA005713 | HB002234 |
| HA006613 | HB003534 |
| HA006633 | HB003581 |
| HA009170 | HB004573 |
| HA009182 | HB006598 |
| HA009505 | HB006600 |
| HA009506 | HB006601 |
| HA011230 | HB008133 |
| HA012014 | HB008134 |
| HA013576 | HB011194 |
| HA016049 | HB011218 |
| HA016061 | HB011219 |
| HA017203 | HB011220 |
| HA017204 | HB011221 |
| HA017205 | HB011222 |
|  | HB011223 |
|  | HB011224 |
|  | HB011225 |
|  | HB011226 |
|  | HB011227 |
|  | HB011228 |
|  | HB011229 |
|  | HB011233 |
|  | HB011993 |
|  | HB011994 |
|  | HB012719 |
|  | HB012730 |
|  | HB017850 |
|  | HB017851 |
|  | HB019846 |

**References**

1.	Ming, R., VanBuren, R., Wai, C.M. *et al.* The pineapple genome and the evolution of CAM photosynthesis. *Nature Genetics*. 2015;**47**: 1435-42

2.	Feng, J., Zhang, W., Chen, C. *et al.* The pineapple reference genome: Telomere-to-telomere assembly, manually curated annotation, and comparative analysis. *Journal of Integrative Plant Biology*. 2024;**66**: 2208-25

3.	Chen, L., VanBuren, R., Paris, M. *et al.* The bracteatus pineapple genome and domestication of clonally propagated crops. *Nature Genetics*. 2019;**51**: 1549-58

4.	Feng, L., Wang, J., Mao, M. *et al.* The highly continuous reference genome of a leaf-chimeric red pineapple (*Ananas comosus* var. *bracteatus* f. *tricolor*) provides insights into elaboration of leaf color. *G3*. 2022;**12**: jkab452

5.	Yow, A.G., Bostan, H., Castanera, R. *et al.* Improved high-quality genome assembly and annotation of pineapple (*Ananas comosus*) cultivar MD2 revealed extensive haplotype diversity and diversified FRS/FRF gene family. *Genes*. 2021;**13**: 52

6.	Nashima, K., Shirasawa, K., Isobe, S. *et al.* Gene prediction for leaf margin phenotype and fruit flesh color in pineapple (Ananas comosus) using haplotype‐resolved genome sequencing. *The Plant Journal*. 2022;**110**: 720-34
